# Characterization of the Antibacterial Activity of Quinone-Based Compounds Originating from the Alnumycin Biosynthetic Gene Cluster of a *Streptomyces* Isolate

**DOI:** 10.3390/antibiotics12071116

**Published:** 2023-06-28

**Authors:** Leonie Sagurna, Sascha Heinrich, Lara-Sophie Kaufmann, Christian Rückert-Reed, Tobias Busche, Alexander Wolf, Jan Eickhoff, Bert Klebl, Jörn Kalinowski, Julia E. Bandow

**Affiliations:** 1 Applied Microbiology, Faculty of Biology and Biotechnology, Ruhr University Bochum, 44780 Bochum, Germany; leonie.sagurna@rub.de (L.S.); sascha.heinrich@rub.de (S.H.); lara-sophie.kaufmann@rub.de (L.-S.K.); 2 Technology Platform Genomics, Center for Biotechnology, Bielefeld University, 33594 Bielefeld, Germany; christian.rueckert@cebitec.uni-bielefeld.de (C.R.-R.); tbusche@cebitec.uni-bielefeld.de (T.B.); joern@cebitec.uni-bielefeld.de (J.K.); 3 Lead Discovery Center GmbH, 44227 Dortmund, Germany; wolf@lead-discovery.de (A.W.); eickhoff@lead-discovery.de (J.E.); klebl@lead-discovery.de (B.K.)

**Keywords:** secondary metabolites, alnumycin, biosynthetic gene clusters, anthraquinones, *Streptomyces*

## Abstract

Bacteria of the genus *Streptomyces* produce various specialized metabolites. Single biosynthetic gene clusters (BGCs) can give rise to different products that can vary in terms of their biological activities. For example, for alnumycin and the shunt product K115, antimicrobial activity was described, while no antimicrobial activity was detected for the shunt product 1,6-dihydro 8-propylanthraquinone. To investigate the antibacterial activity of 1,6-dihydro 8-propylanthraquinone, we produced alnumycin and 1,6-dihydro 8-propylanthraquinone from a *Streptomyces* isolate containing the alnumycin BGC. The strain was cultivated in liquid glycerol–nitrate–casein medium (GN), and both compounds were isolated using an activity and mass spectrometry-guided purification. The structures were validated via nuclear magnetic resonance (NMR) spectroscopy. A minimal inhibitory concentration (MIC) test revealed that 1,6-dihydro 8-propylanthraquinone exhibits antimicrobial activity against *E. coli* Δ*tolC*, *B. subtilis*, an *S. aureus* type strain, and a vancomycin intermediate-resistance *S. aureus* strain (VISA). Activity of 1,6-dihydro 8-propylanthraquinone against *E. coli* Δ*tolC* was approximately 10-fold higher than that of alnumycin. We were unable to confirm gyrase inhibition for either compound and believe that the modes of action of both compounds are worth reinvestigating.

## 1. Introduction

Over the last few decades, natural products served as inspiration for the development of modern therapeutics. Today, half of all drugs and more than 75% of the antibiotics in use originate from natural products [1,2]. The discovery of novel drugs is plagued by the rediscovery of already known compounds. Arguably, the bioactivity of many compounds—even known ones—has not been fully exploited. Bacteria of the genus *Streptomyces* are known for producing a great variety of bioactive compounds [3,4,5,6,7]. This chemical diversity originates from biosynthetic pathways encoded in biosynthetic gene clusters (BGCs). The major classes of natural products encompass non-ribosomal peptides (NRP), ribosomally synthesized and post-translationally modified peptides (RiPPs), terpenes, alkaloids, saccharides, and polyketides [8,9]. Notably, polyketides recently gained much attention, since they exhibit structural diversity and have a broad range of bioactivities [10]. Several clinically important antibiotics, such as tetracyclines or erythromycin, are of polyketide origin.

**Figure 1:**
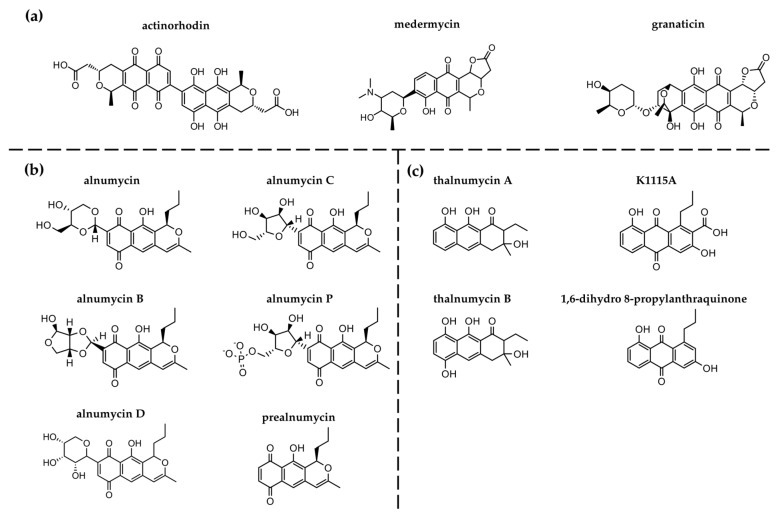
Compounds related to alnumycin. (**a**) Benzoisochromanequinones structurally related to alnumycin. (**b**) Alnumycin derivatives and biosynthetic intermediates. (**c**) Predicted shunt products of alnumycin biosynthetic pathway.

The isochromanequinone alnumycin A (alnumycin) was first isolated in 1998 from *Streptomyces sp.* DSM 11575 and *Streptomyces griseorubiginosus* Mer-K1115, known as K1115 B_1_, and investigated for its interesting structural features [11,12]. Although it is structurally related to benzoisochromanequinone polyketides actinorhodin, medermycin, and granaticin (Figure 1a), alnumycin shows unusual features [13,14,15,16,17,18]. In addition to lacking both the first carbon component of the polyketide chain and a substitution of the chiral carbon at position two with a double bond between carbons two and three, alnumycin possesses a sugar-like 4-hydroxymethyl 5-hydroxy 1,3-dioxan moiety at position nine, which is highly uncommon in natural products. The biosynthesis of the latter component is still not fully understood [19,20]. However, alnumycin is not only a substance of interest due to its structural properties, but also because it exhibited various biological activities, ranging from antibacterial to cytostatic traits. In a German patent application by Bieber and Nueske, alnumycin was shown to inhibit the DNA gyrase subunit B (GyrB) of *E. coli* in vitro. An IC_50_ in the range of 48–120 µM was reported [21]. Studies of the biosynthetic pathway of alnumycin in *Streptomyces* sp. CM020 indicated that analogues of alnumycin can be produced during its biosynthesis (Figure 1b). Alnumycin and the analogues B and D were shown to inhibit biofilm formation of *Staphylococcus aureus* [22]. The inactivation of two genes in the alnumycin BGC, *alnT* and *alnH*, which encode for mono-oxygenase and flavin reductase, respectively, caused the accumulation of a novel non-quinoid metabolite: thalnumycin A (Figure 1b) [23]. In vitro studies that use mono-oxygenase and flavin reductase with thalnumycin A as a substrate resulted in the formation of a unique compound known as thalnumycin B, which has the respective *p*-hydroquinone structure found in alnumycin (Figure 1c) [23]. No antibacterial activity was reported for these compounds. Moreover, the production of the anthraquinones K1115 and 1,6-dihydro 8-propylanthraquinone was found to be associated with the alnumycin biosynthetic pathway in *Streptomyces* sp. CM020. Deletion of either *aln4* and *aln5* or *alnT* and *alnH* in the alnumycin BGC led to the synthesis of two already known anthraquinones: K1115 and 1,6-dihydro 8-propylanthraquinone (Figure 1c) [19,23]. Both compounds were previously isolated from the marine-derived *Streptomyces* sp. FX-58, *Streptomyces* sp. B8000, and *Micromonospora rhodorangea*, for which genome sequences are not available in NCBI [24,25,26]. Antimicrobial activity against the methicillin resistant strain *S. aureus* ATCC43300 was described for K1115, though it was not described for 1,6-dihydro 8-propylanthraquinone [25,27]. However, 1,6-dihydro 8-propylanthraquinone appears to be especially promising from a structural perspective. Multiple quinolones, including nalidixic acid and the fluoroquinolones ciprofloxacin and levofloxacin, inhibit gyrase-mediated supercoiling reactions during replication by interacting with the enzyme [28,29,30,31,32,33]. Other quinolones of the class of anthraquinones exhibit antibacterial activity against gram-negative and gram-positive bacteria [27,34,35,36,37,38,39,40,41,42,43]. Emodin, for example, is a natural anthraquinone extracted from the roots of *Rheum palmatum* L and shows strong antibacterial activity against a methicillin-resistant *Staphylococcus aureus* strain [44]. Prior comprehensive proteomic analysis revealed that emodin treatment caused an imbalance in the pyruvate metabolism, protein biosynthesis inhibition, and DNA synthesis suppression [45]. In another study, emodin and other natural anthraquinones extracted from rhubarb, like rehin and aloe-emodin, were found to exhibit antimicrobial activity against the gram-negative bacterium *Aeromonas hydrophila* [37]. Mode of action studies regarding emodin show that it can increase the membrane permeability in vivo [37]. In vitro tests also show that emodin can bind DNA [37]. Substituents at the C-2 position of the anthraquinone backbone were discussed to determine antibacterial activity. Therefore, we were interested in investigating quinolone-derived compounds, such as 1,6-dihydro 8-propylanthraquinone, for their biological activities. By screening environmental isolates for the presence of an alnumycin BGC in the genome, we identified isolate CS 39 as a potential producer. We then tested different cultivation conditions, and we purified alnumycin and 1,6-dihydro 8-propylanthraquinone in preparation for investigations into their antibacterial activities and ability to inhibit gyrase.

## 2. Materials and Methods

### 2.1. Isolation of Strains

The soil sample was collected in Germany at 51°15′52.9″ N 7°09′39.6″ E. The soil was stamped eight times using an enrichment agar (GN agar: 1.8% (*w*/*v*) agar, which contained 108.6 mM glycerol, 0.3 g/L casein (Difco, vitamin free), 19.8 mM KNO_3_, 34.2 mM NaCl, 11.5 mM K_2_HPO_4_, 0.2 mM MgSO_4_, 0.2 mM CaCO_3_, 0.07 mM FeSO_4_, and pH 7.0–7.2) [46]. The plates were incubated for 5 days at 30 °C. Single colonies that showed typical streptomycete morphology were transferred to soy flour–mannitol liquid medium (SFM: 20 g/L soy flour (Hensel), 109.8 mM D-mannitol) and incubated for 7 days at 30 °C and 180 rpm. Mycelia were plated on 2% (*w*/*v*) SFM agar and incubated at 30 °C until spore development was observed. Spores were removed from the plates with 20% glycerol and preserved as spore suspension for further analysis.

### 2.2. Genomic Analysis of Strains

Isolate CS 39 was cultivated for 3 days at 30 °C and 180 rpm in DNA-isolation medium that was mixed 3:1 with yeast extract malt extract complex medium (YEME: 3 g/L yeast extract (Thermo Fisher, Waltham, MA, USA), 5 g/L peptone (Thermo Fisher), 3 g/L malt extract (Thermo Fisher), 55.5 mM glucose, and 73 mM saccharose) to which 5 mM MgCl_2_ × 6 H_2_O and 0.5% glycerol were added, as well as in tryptic soy broth yeast extract complex medium (TSBY: 30 g/L tryptic soy broth (Sigma-Aldrich, St. Louis, MO, USA), 3 g/L yeast extract (Thermo Fisher)) [47]. Genomic DNA was extracted using the NucleoSpin Microbial DNA Kit (Macherey-Nagel, Düren, Germany). Extracted genomic DNA was used to create an SQK-LSK109 library for ONT nanopore sequencing and a TSPf library for Illumina. The ONT library was sequenced on a R9.4.1 flowcell using a GridION sequencer and basecalled with guppy v4.0.11 in high accuracy mode, and the TSPf library was run using a MiSeq sequencer in a 2 × 300 nt run. Based on the genome length, 123.1-fold coverage ONT data and 97.2-fold coverage Illumina data were assembled using the software package canu v1.8 [48] for ONT data and newbler v2.8 [49] for Illumina data, as well as unicycler v0.4.6 [50] for both data sets. For phylogenetic analysis, the 16s rDNA was extracted using RNAmmer 1.2. Evolutionary distances were calculated using the single-gene tree calculator provided by the DSMZ [51,52]. For the prediction of BGCs, the genome was analyzed using antiSMASH version 6.0 [53,54]. The detection parameters were adjusted to relaxed settings, and all extra features were used. While the full genome sequence will be published elsewhere, the sequence of the alnumycin BGC was submitted to NCBI (accession number OQ633075) and used for BLAST analysis by searching for streptomycete genomes using the BLAST server (version 2.13.0) [55].

### 2.3. Screening for Compound Production

To investigate the production of alnumycin and 1,6-dihydro 8-propylanthraquinone, isolate CS 39 was cultivated under seven different cultivation conditions: GN liquid medium, GN liquid medium with added heat-killed *E. coli* EP1581 cells, International *Streptomyces* Project 2 solid medium (ISP2: 22.2 mM glucose, 4 g/L yeast extract, 10 g/L malt extract, 2% (*w*/*v*) agar), PG3 liquid medium (PG3: 7.3 mM KH_2_PO_4_, 27.7 mM glucose, 108.6 mM glycerol, 90 g/L dextrin (Carl Roth), 10 g/L soy flour, 10 g/L peptone, 10 mM CaCO_3_, 1 g/L PEG 1000 (Sigma-Aldrich); pH 6.5), and SFM liquid medium. Next, 5 × 10^5^ spores were used to inoculate 10 mL liquid or solid medium. Liquid and solid cultures were cultivated for 14 days at 30 °C, and liquid cultures were kept under constant agitation (180 rpm). Supplemented liquid media with heat-killed *E. coli* EP1581 cells were prepared as follows: 5 × 10^5^ spores were added to 10 mL of no-citrate Belitzky minimal medium (NCBMM: 21 mM NaCl, 15 mM (NH_4_)_2_SO_4_, 8 mM MgSO_4_, 27 mM KCl, 50 mM Tris, 0.6 mM KH_2_PO_4_, 2 mM CaCl_2_, 0.01 mM MnSO_4_, 0.001 mM FeSO_4_, 4.5 mM L-glutamate, 0.78 mM L-tryptophan, 11 mM D-glucose, pH 7.5) and incubated for 7 days at 30 °C and 180 rpm. *E. coli* EP1581 was then inoculated in 10 mL Luria–Bertani medium (LB: 10 g/L tryptone (Gibco, Bacto), 171 mM NaCl, 5 g/L yeast extract) [56] and grown at 37°C and 200 rpm to an OD600 of 0.5–1.0. Next, cultures were centrifuged at 4 °C and 11,000 rpm for 10 min, and the supernatant was removed. Cells were washed with an equal volume to the previous culture volume with 0.9% NaCl solution and diluted to 1 × 10^7^ cells/mL. Cells were then boiled for 30 min. In total, 10 mL of GN liquid medium were supplemented with 250 µL of heat-killed cells and inoculated with 500 μL of the pre-cultures of CS 39. Finally, the cultures were incubated at 30 °C and 180 rpm for 14 days.

Mycelia were separated from liquid culture supernatant via filtration and, subsequently, extracted using methanol and ethyl acetate. Organic extraction of the supernatant/filtrate was performed using ethyl acetate. Produced metabolites were extracted from the agar by cutting the agar into small slices and, subsequently, stirring overnight at 500 rpm and room temperature in ethyl acetate. The organic fractions were dried in vacuo, and the residuals were redissolved in methanol to 500 ng/µL. Mass spectrometry was performed using a Vion IMS QTOF with an attached ESI source. The setup was coupled to an Acquity UHPLC I-class, and compound separation was achieved via a HSST3 C18 column (150 mm × 2.1 mm, 1.8 µm) using a H_2_O/CH_3_CN gradient, each with 0.1% formic acid (FA). Mass spectra were recorded in positive and negative ionization modes. The experiments were performed in biological triplicates (*n* = 3). Standard deviations were calculated based on measured intensities, and the significance of differences was calculated compared to extracts of the CS 39 GN culture supernatants using Student’s *t*-test.

### 2.4. Activity and Mass Spectrometry-Guided Purification of Alnumycin and 1,6-Dihydro 8-Propylanthraquinone

The purification of both compounds was optimized using an extract of a 1 L culture of isolate CS 39. To this end, the cultivation was performed in GN liquid medium for 14 days at 30 °C and 180 rpm. Supernatant and mycelia were separated, and the supernatant was extracted with an equal volume of ethyl acetate and, subsequently, evaporated. Mycelia were first mixed with one volume of methanol and extracted at 160 rpm and 40 °C. After filtration, the solvent was evaporated, and the residual was redissolved in one volume ethyl acetate and water (1:1 *v*/*v*). The organic phase was separated, dried over Na_2_SO_4_, and evaporated in vacuo. The crude extract was loaded onto silica gel and further separated on a C18 flash cartridge (26 g) using a H_2_O/CH_3_CN gradient, each with 0.1% FA (Combi*Flash* RF (Teledyne Technologies, Thousand Oaks, CA, USA) and a flow rate of 35 mL/min). Fractions were collected based on the UV trace at 210 nm. After evaporation, stock solutions (10 mg/mL) in methanol were used to screen for antibacterial activity against *E. coli* Δ*tolC*, *B. subtilis* 168, *S. aureus* DSM 20231, and *S. aureus* Mu50. The test strains were grown in Mueller–Hinton broth to an OD_600_ of 0.5–1.0. Next, 5 × 10^5^ cells/mL were exposed to a 100 µg/mL final concentration of the extracts in microtiter plates (200 µL final volume) and incubated overnight for 16–18 h at 37 °C. The growth of the cells was determined by reading OD_600_ using a plate reader. In addition, all fractions were analyzed using mass spectrometry, as described in the above section, and screened for alnumycin and 1,6-dihydro 8-propylanthraquinone. Fractions showing the strongest inhibitory effects, while giving the highest intensities of either compound in mass spectrometry, were used for further separation via preparative liquid chromatography with a Nucleodur C18 Isis column (5 µm, 150 mm × 21 mm, Macherey–Nagel), collecting fractions of 18 mL with a flow rate of 12 mL/min using a H_2_O/CH_3_CN gradient, each with 0.1% FA (Combi*Flash* RF). The fractions were again tested for antibacterial activity, and the purity of active fractions was analyzed by mass spectrometry. To purify the compounds from an extract of an 8 L culture volume, the method was transferred and scaled-up to a preparative system (Agilent Technologies, Ratingen, Germany; Nucleodur C18 Isis column, 5 µm, 150 mm × 21 mm, Macherey–Nagel; flow rate 21 mL/min, H_2_O/CH_3_CN gradient, each with 0.1% FA).

### 2.5. Minimal Inhibitory Concentration

The minimal inhibitory concentrations of the compounds against *E. coli* DSM 30083, *E. coli* Δ*tolC*, *B. subtilis* 168, *S. aureus* DSM 20231, *S. aureus* Mu50, and *Acinetobacter baumannii* DSM 30007 were determined in biological triplicates (*n* = 3) using a microdilution assay in microtiter plates with Mueller–Hinton broth, following the recommendations of the Clinical and Laboratory Standards Institute [57].

### 2.6. Structure Elucidation via Nuclear Magnetic Resonance Spectroscopy (NMR)

The NMR spectra of the isolated compounds were recorded using an AV III 300 (300 MHz) and a DRX-600 (600 MHz) instrument (Bruker Daltonik, Billerica, MA, USA). The solvents used were chloroform-d_1_ and acetone-d_6_ from Eurisotop (Saint-Aubin, France). The spectra obtained were referenced using the residual signals (CDCl_3_: δ (^1^H) = 7.26 ppm and δ(^13^C) = 77.16 ppm; acetone-d_6_: δ (^1^H) = 2.05 ppm and δ(^13^C) = 29.84 ppm). MestreNova 14.2.1 purchased from Mestrelab Research S.L. (Santiago de Compostela, Spain) was used to process the spectra.

### 2.7. Gyrase Inhibition Assay

Gyrase inhibition was tested by employing two different assays—a gel-based assay and a luciferase-based assay. In both assays, the isolated gyrase from the overproducing *E. coli* strains JMtacA and JMtacB, which was applied as a A_2_B_2_ complex, was used (Inspiralis, Norwich, UK) [58]. For the gel-based assay, reactions were performed using 2.23 nM gyrase and 0.5 µg of relaxed pBR322 as a substrate per reaction. Stock solutions of the tested compounds were prepared in DMSO (50 mM). Inhibitors found at different concentrations were added to the reaction at a volume of 0.3 µL. The final reaction volume was 30 µL, and the final DMSO concentration was 1%. Reactions were incubated at 37 °C and 300 rpm for 30 min. Subsequently, 30 µL of STEB and 30 µL of chloroform/isoamyl alcohol (*v*:*v*, 24:1) were added to stop the reaction. After mixing, centrifugation was used to separate the aqueous and organic layers (1 min, 6000× *g*, RT). Next, 20 µL of the aqueous phase were loaded onto a 1% (*w*/*v*) agarose gel and run at 85 V. Gels were stained for 20 min with GelRed (Merck Millipore, Burlington, VT, USA) (1:3300 *v*/*v* in 0.1 M NaCl solution) and detected at the ChemiDoc cell station (Bio-Rad, Hercules, CA, USA). For the luminescence-based assay, 10 nM gyrase, 0.025 µg/µL relaxed pNO1, 265 µM ATP, and the respective test compounds (serial dilution starting from 10 µM) were mixed in 384-well plates and incubated at 37 °C for 30 min. After incubation, 5 µL of ADP-Glo reagent (Promega Cat.V9101) was added, and the reactions were incubated for 40 min at RT. Next, 10 µL of the kinase detection reagent (Promega, Madison, WI, USA) was added, and the solution was incubated for a further 40 min at RT. The luminescence was detected using an EnVision plate reader (PerkinElmer, Waltham, MA, USA) with the emission filter luminescence 700 nm and a measurement time of 0.25 s. Both assays were performed in technical triplicates (*n* = 3).

## 3. Results

### 3.1. Genetic Analysis of the Alnumycin BGC

**Figure 2:**
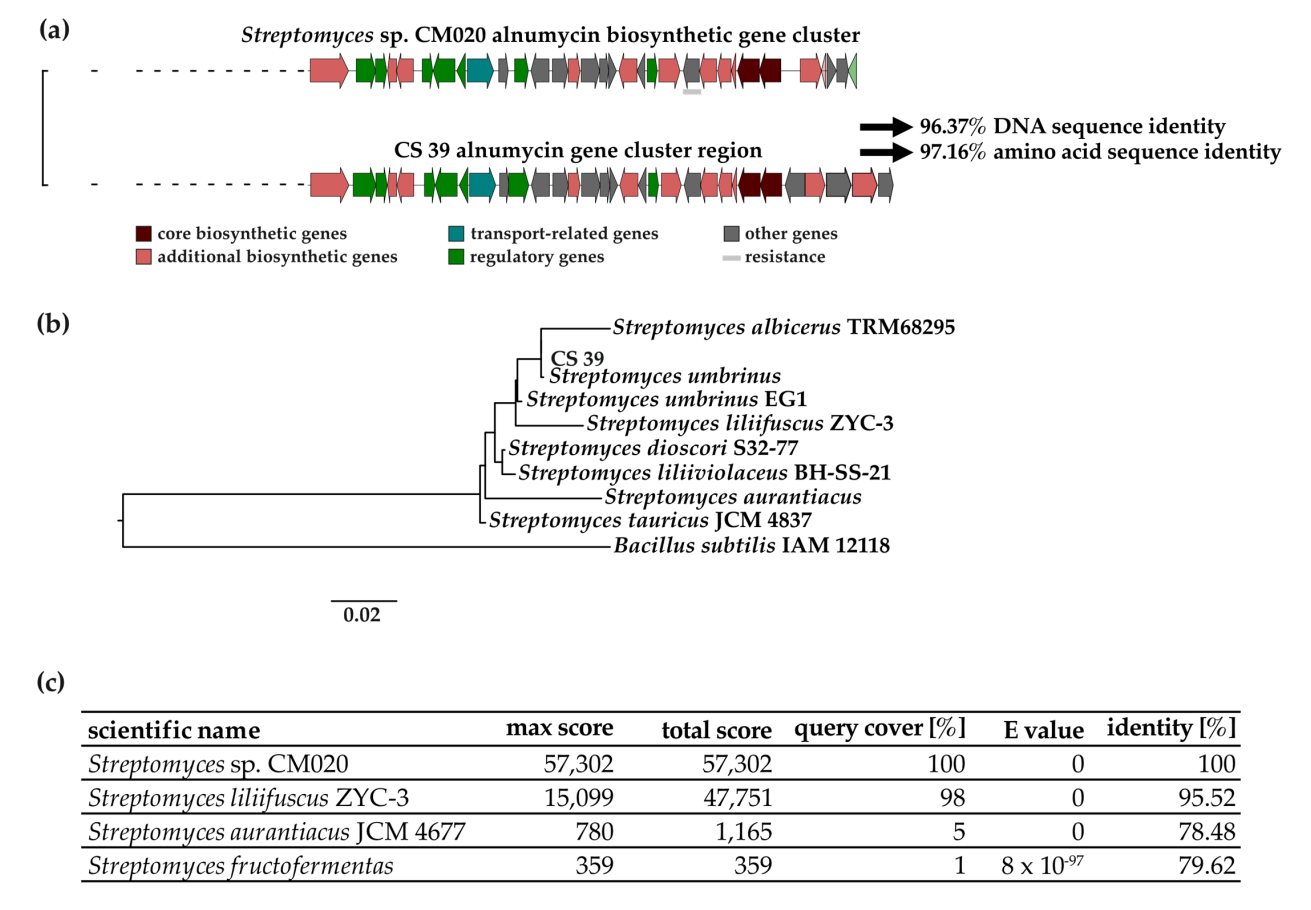
Genetic analysis of alnumycin BGC. (**a**) The alnumycin BGC of *Streptomyces* sp. CM020 was compared to predicted alnumycin BGC of isolate CS 39 (NCBI accession number OQ633075). It showed a 96% gene similarity (antiSMASH) and 97.65% DNA and 97.16% amino acid sequence similarity in BLASTN and BLASTX analysis (Figure S1). (**b**) Phylogenetic tree based on 16s rDNA sequences from seven related type strains and *B. subtilis* NR_112116 as outgroup (*Streptomyces umbrinus* and *Streptomyces aurantiacus* were first described as *Streptomyces ederensis* NRRL B-8146 and *Streptomyces glomeroaurantiacus* MD12-408-1-A, respectively). Evolutionary distances were calculated using single-gene trees calculator of DSMZ. Distance formula d5 was used to calculate branch lengths between strains. *Streptomyces* strains displayed in the phylogenetic tree show average similarities of 97.53%. The 16s rDNA sequence of isolate CS 39 can be downloaded from NCBI (accession number OQ632519) [52,59]. Respective similarity values of formula d5 can be found in Table S1. (**c**) First three database hits of sequence similarities of the alnumycin BGC from *Streptomyces* sp. CM020 (NCBI accession number EU852062) based on BLASTN analyses.

A nucleotide sequence comparison performed with the Basic Local Alignment Search Tool (BLAST) using the BGC of *Streptomyces* sp. CM020 as a query input showed that the alnumycin BGC is present in 3% of streptomycetal genomes accessible through the NCBI platform. In our study, environmental *Streptomyces* isolate CS 39 was identified as containing the alnumycin BGC. The antiSMASH analysis highlighted a 96% similarity between genes in the alnumycin gene cluster, which was predicted for the genome of isolate CS 39, and the alnumycin BGC from *Streptomyces* sp. CM020 recorded in the MiBiG library (Figure 2a). BLASTN and BLASTX (comparison of translated nucleotide with protein sequence) analysis of the CS 39 alnumycin BGC calculated a 97.65% DNA sequence identity and an average of 97.16% amino acid identity with the published alnumycin BGC of *Streptomyces* sp. CM020 (Figure 2a, Figures S1 and S2). An investigation into the phylogeny of isolate CS 39 based on 16s rDNA analysis showed that the isolate belongs to the genus *Streptomyces* (Figure 2b). The highest similarity of 99.93% was calculated for *Streptomyces umbrinus* (first described as *Streptomyces ederensis* NRRL B-8146), which indicates that the isolate CS 39 belongs to this species (Figure 2b, Table S1). No alnumycin BGC was found in known *S. umbrinus* strains, though it was found in three other strains belonging to other *Streptomyces* species (Figure 2c). The closest relative of CS 39, for which an alnumycin BGC was identified with 95.52% similarity to that of *Streptomyces* sp. CM020, was *S. liliifuscus* ZYC-3 (Figure 2c, Table S1), which shares a 99.22% similarity in 16s rDNA. For the other strains that are closely related to isolate CS 39 based on 16s rDNA, similarities of less than 80% were calculated on the DNA level for the alnumycin BGC (Figure 1c).

### 3.2. Screening for Compound Production

**Figure 3:**
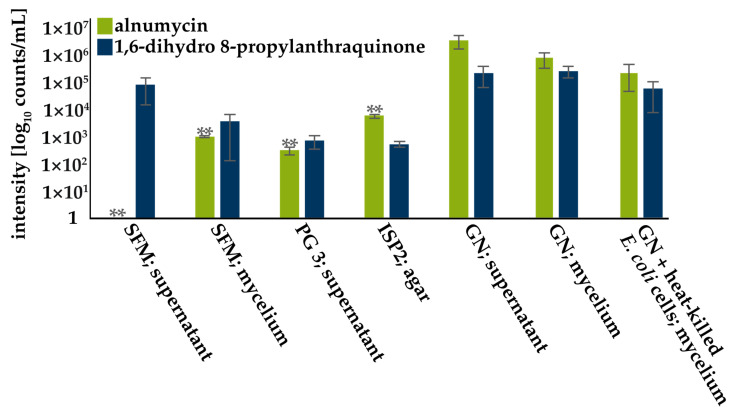
Screening for alnumycin and 1,6-dihydro 8-propylanthraquinone production. Isolate CS 39 was grown under seven different cultivation conditions. Supernatant and mycelial extracts were screened for production of both compounds using mass spectrometry. Green bars indicate intensities for alnumycin normalized to cultivation volume in log_10_ counts/mL culture volume, while blue bars show intensities for 1,6-dihydro 8-propylanthraquinone. Normalized intensities [counts/mL] can be found in Table S2. Standard deviations represent biological triplicates (*n* = 3), while significance of differences were calculated in comparison to data of extracted GN culture supernatants using Student’s *t*-test. (**; *p* < 0.05).

To identify suitable production conditions for alnumycin and 1,6-dihydro 8-propyl-anthraquinone, different cultivation conditions were compared for yields based on mass signal intensities (Figure 3, Table S2). In five of the eight tested cultivation conditions, at least one of the two compounds could be detected (Figure 3, Table S2). Low intensities, and, thus, presumable low amounts of alnumycin or 1,6-dihydro 8-propylanthraquinone, were detected in supernatants and mycelia of the cultivation in SFM medium, as well as on ISP2 agar (Figure 3, Table S2). In PG 3 medium, alnumycin was only detected in the supernatant and at low intensities (Figure 3, Table S2). The highest intensities for both compounds were detected in mycelia and the supernatant of CS 39 cultivated in GN mini-mal medium (Figure 3, Table S2). The addition of heat-killed *E. coli* EP1581 cells to GN medium did not influence production of either compound (Figure 3, Table S2). Thus, compared to the highest alnumycin production level in GN medium (supernatant), significantly lower amounts were observed for PG3 (supernatant), ISP2 (agar), and SFM (mycelium) media.

### 3.3. Purification and Structural Validation of the Compound

**Figure 4:**
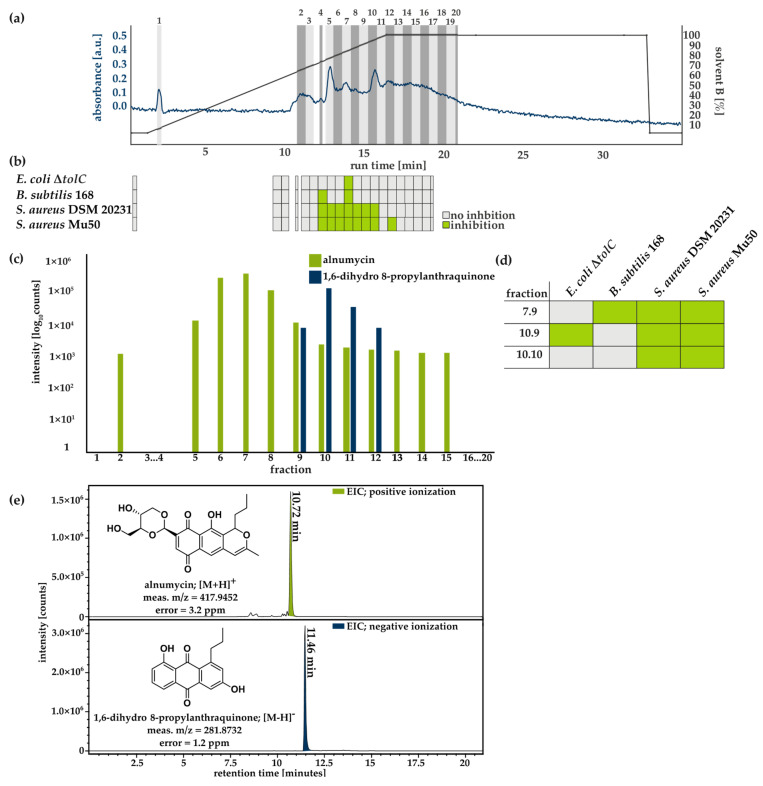
Activity and mass spectrometry-guided purification of alnumycin and 1,6-dihydro 8-pro-pylanthraquinone. To establish purification of both compounds, (**a**) crude extract of mycelial extract of 1 L-culture was separated by flash chromatography using a C18 flash cartridge (26 g stationary phase), using a H_2_O/CH_3_CN gradient with 0.1% FA and a flow rate of 35 mL/min. Fractionation was based on UV signals at 210 nm. (**b**) Resulting fractions were tested for antibacterial activity against *E. coli* Δ*tolC*, *B. subtilis* 168, *S. aureus* DSM 20231, and *S. aureus* Mu50 in biological triplicates (*n* = 3). Grey boxes represent no growth inhibition, while green color indicates inhibition of growth by at least 50% of strains compared to untreated controls. OD_600_ (mean values) and standard deviations are given in Table S3. (**c**) Fractions were screened for alnumycin and 1,6-dihydro 8-propylanthraquinone via mass spectrometry (green bars alnumycin; blue bars 1,6-dihydro 8-propylanthraquinone, *n* = 1). Intensities are displayed on a logarithmic scale. Intensities are summarized in Table S4. (**d**) Fractions of previous flash chromatography analyses were separated further via preparative liquid chromatography using Combi*Flash* RF system with a Nucleodur C18 Isis column. Fractions of 18 mL were collected with a flow rate of 12 mL/min using a H_2_O/CH_3_CN gradient with 0.1% FA. Resulting fractions were tested for antibacterial activity, with green boxes indicating inhibition by at least 50% and grey boxes indicating no inhibition (OD_600_ mean values and standard deviations are given in Tables S5 and S6). (**e**) Cultivation was scaled to 8 L cultivation volume (*n* = 1), and the purification method was transferred to a preparative liquid chromatography system created by Agilent Technologies (Nucleodur C18 Isis column, flow rate 21 mL/min, H_2_O/CH_3_CN gradient with 0.1% FA). Purity was proven using mass spectrometry. Structures of purified compounds were validated by NMR analysis. NMR spectra and structure annotation can be found in Tables S7 and S8 and Figures S3–S13.

In order to establish a purification protocol, an extract of a 1 L GN culture incubated for 14 days was separated into 20 fractions by flash-chromatography using a C18 flash cartridge (26 g), a H_2_O/CH_3_CN gradient, each with 0.1% formic acid (FA), and a flow rate of 35 mL/min in a single experiment. Fractions were collected based on the measured absorbance at 210 nm (Figure 4a). All fractions were tested for antibacterial activity against different test strains at a final concentration of 100 µg/mL and analyzed using mass spectrometry (Figure 4b,c). For 8 of the 20 fractions, antibacterial activity was observed against at least one of the strains (Figure 4b). While the growth of *E. coli* Δ*tolC* was only inhibited by fraction 10, *B. subtilis* 168 was inhibited by fractions 7 and 10 (Figure 4b). Inhibition of *S. aureus* DSM 20231 and *S. aureus* Mu50 was observed for fractions 7, 8, 9, 10, 11, 12, and 13, and for *S. aureus* Mu50, it was also observed for fraction 15 (Figure 4b, Table S3). Mass spectrometry analysis showed that the highest intensities for the masses of alnumycin and 1,6-dihydro 8-propylanthraquinone were found in the most active fractions 7 and 10 (Figure 4b,c). Fractions 7 and 10 were further separated using preparative liquid chromatography, with a C18 Isis column collecting fractions of 18 mL at a flow rate of 12 mL/min and a H_2_O/CH_3_CN gradient with 0.1% FA. Three of the resulting fractions showed antimicrobial activity (Figure 4d, Table S5). Fraction 7.9 inhibited *B. subtilis* 168 and both *S. aureus* strains, while fractions 10.9 and 10.10 inhibited both *S. aureus* strains; fraction 10.9 also inhibited *E. coli* Δ*tolC* (Figure 4d, Table S6). Mass spectrometry analysis revealed that fraction 7.9 contained alnumycin and fractions 10.9 and 10.10 contained 1,6-dihydro 8-propylanthraquinone, with each element having a purity of ~98% (Figure 4d). After scaling up production and purification, the structures of both substances were elucidated and verified by NMR spectroscopy (Figure 4e).

### 3.4. Spectrum of Activity of Alnumycin and 1,6-Dihydro 8-Propylanthraquinone

The antibacterial spectrum of the two substances was repeatedly tested at defined final concentrations against the four test strains previously reported, as well as *E. coli* DSM 30083 and *A. baumannii* DSM 30007 (Table 1). No inhibition by either compound was observed against *E. coli* DSM 30083 and *A. baumannii* DSM 30007. Alnumycin had an MIC against *E. coli* Δ*tolC* of 100 µg/mL, while a 50% reduction in final OD_600_ was observed for *B. subtilis* 168 and *S. aureus* Mu50 at a concentration of 8 µg/mL (Table 1). The MIC of alnumycin for *S. aureus* DSM 20231 was 10 µg/mL. Interestingly a 50% final OD_600_, was observed for 1,6-dihydro 8-propylanthraquinone against *E. coli* Δ*tolC* and both *S. aureus* strains at 10 µg/mL (the same results as for alnumycin) (Table 1). For *B. subtilis* 168, an MIC of 10 µg/mL was observed for 1,6-dihydro 8-propylanthraquinone (Table 1).

Since the antibacterial effect of alnumycin was previously attributed to gyrase inhibition [21], we tested both alnumycin and 1,6-dihydro 8-propylanthraquinone using in vitro gyrase inhibition assays. To this end, we used a commercially available gyrase inhibition assay (Inspiralis) and compared the activities of alnumycin and 1,6-dihydro 8-propylanthraquinone against *E. coli* gyrase using novobiocin as a comparator compound (Figure S14a). While novobiocin was active at 1 µM, no activity was observed for alnumycin or 1,6-dihydro 8-propylanthraquinone up to a concentration of 500 µM (Figure S14a). Indeed, this finding was confirmed in a second, luminescence-based assay, in which we found no evidence of gyrase inhibition up to 10 µM (Figure S14b).

## 4. Discussion

### 4.1. Alnumycin and 1,6-Dihydro 8-Propylanthraquinone Production

The alnumycin BGC was identified in 3% of genomes of streptomycetal origin accessible in the NCBI database. In the literature, alnumycin production was reported to be promoted in cultivation media containing glucose, NH_4_Cl, Na_2_HPO_4_, KH_2_PO_4_, MgSO_4_, iron EDTA, NaCl, and Frankia trace element solution containing a mixture of metal ions [21]. By screening different media for alnumycin production, we found production levels to be highly dependent on the media composition. Based on measured intensities and standard deviations of the masses of alnumycin and 1,6-dihydro 8-propylanthraquinone, the highest yields were obtained in GN medium (Figure 2). GN medium contained casein and potassium nitrate as nitrogen sources and glycerol as a carbon source; trace elements Na, Ca, Fe and Mg were supplemented to the medium, and the pH was adjusted to 7.0–7.2 [46]. Reported amino acid and elemental analyses of the used casein (vitamin-free, Difco) in the cultivation medium showed that the most abundant amino acids were histidine, proline, and glutamic acid, and the most abundant elements were Ca, Cl, K, Mg, Na, P, and S [60,61]. Unlike GN medium, ISP2 contains glucose and malt extract as carbon sources. In addition, the yeast extract serves as a nitrogen source. Composition analysis of malt extract found that it mainly (60%) consists of reduced sugars, such as maltose (Sigma-Aldrich). It also contains B vitamins, various amino acids that can also be used as nitrogen sources, and trace elements, like Mg and K [62]. The main components of the yeast extract are amino acids [63]. Multiple minor elements can be found in yeast extracts, namely mainly Fe, Ca, Mg, and Zn [64]. Lowest production was observed in SFM and PG 3 complex media. SFM contains mannitol and glucose as defined carbon sources. Soy flour consists of 36% protein, 24% fat (mainly polyunsaturated), 15% carbohydrates, 11% fiber, and 14% trace elements (Hensel). Amino acids, like valine, isoleucine, tyrosine, alanine, and glutamic acid, can be found in soy flour. Al, Ca, Cu, K, Mg, Mn, Mo, Ni, and Zn are also found in soy flour [65]. Various carbon sources are found in PG 3, like glycerol, glucose, and dextrin, which are mixtures of polymers of D-glucose. Like in SFM medium, soy flour served as a nitrogen source. As an additional nitrogen source, soy peptone is found in PG 3, consisting of 55% amino acids, especially glutamic acid and asparagine [66]. In addition to the trace elements found in soy flour, P and Ca are supplemented to the medium. The pH is adjusted to 6.5. The main differences in media composition pertain to the nitrogen source: while in GN medium, casein is used, the other media contain yeast- or soy-based nitrogen sources. In particular, comparisons of soy and casein in the literature show stark differences in terms of the amino acid composition of casein and soy-based peptone, highlighting the high proline concentrations found in casein sources [66]. In the plant *Rubia tinctorium*, enhanced anthraquinone production was observed after the addition of proline [67]. It remains unclear if proline has similar effects on anthraquinone production in bacteria, or if other triggers are responsible for the activation of the alnumycin biosynthetic gene cluster. The pH might also play a role in the activation of the alnumycin biosynthetic pathway. For *Streptomyces coelicolor* A3(2), an effect was seen related to the biosynthesis of actinorhodin, which is an antibiotic related to alnumycin. Actinorhodin was produced after a pH shift from pH7 to pH4 [68]. Systematic studies on the regulation of alnumycin production could shed light on correlations between media components or cultivation conditions and alnumycin production.

### 4.2. Antibacterial Activity of Alnumycin and 1,6-Dihydro 8-Propylanthraquinone

Alnumycin and some of its derivatives (alnumycin b, c, d, prealnumycin) were previously described as antimicrobial agents, including K1115, which is a substance associated with alnumycin production [21,25]. 1,6-dihydro 8-propylanthraquinone did not show antibacterial activity against *B. subtilis*, *S. aureus* or *Streptomyces viridochromogenes* (Tü 57) in an agar diffusion assay [25]. In this study, we found 1,6-dihydro 8-propylanthraquinone to be active against *E. coli* Δ*tolC*, *B. subtilis* 168, *S. aureus* DSM 20231, and *S. aureus* Mu50 in a microtiter plate assay with MIC values of 10 µg/mL for *E. coli* Δ*tolC*, 8 µg/mL for *S. aureus* Mu50, 10 µg/mL for *S. aureus* DSM 20231, and *B. subtilis* 168 (Figure 4). Antimicrobial activity of natural anthraquinones, like emodin, aloe-emodin and rhein, was previously reported [37,44]. We found that 1,6-dihydro 8-propylanthraquinone exhibited MICs comparable to those of alnumycin against all tested bacterial strains, except for *E. coli* Δ*tolC*, against which it was approximately 10-fold more active (Table 1). While both compounds showed antibacterial effects against the *E. coli* mutant lacking the TolC efflux pump, no inhibition could be observed against the TolC-competent *E. coli* type strain (Figure 4). Thus, resistance to both compounds likely relies on efflux. Alnumycin was reported in a German patent application to inhibit *E. coli* gyrase B subunit in vitro [21]. To identify the mechanism of action of 1,6-dihydro 8-propylanthraquinone, we assessed whether 1,6-dihydro 8-propylanthraquinone and alnumycin exhibited similar inhibitory effects on gyrase. For neither compound were we able to detect any inhibition of *E. coli* gyrase (Figure S12), leading to the conclusion that, at least in *E. coli* Δ*tolC*, another mechanism must be at play. Inhibition of topoisomerase I or IV was not tested either by Bieber and Nueske (German patent application) or in this study. For emodin, it was previously reported that the proteomic response of *Staphylococcus aureus* MRSA to treatment indicated a disturbance of metabolic processes due to an imbalance in the pyruvate metabolism, as well as inhibition of protein and DNA synthesis inhibition [44]. In addition, in vitro assays demonstrated that emodin binds to DNA of *Aeromonas hydrophila*, and it was shown to increase membrane permeability in vivo [37]. Comparative analyses of the alnumycin derivatives pre-alnumycin, as well as alnumycins b, c, and d, revealed efficacy against *S. aureus* biofilms [22]. Similar effects were described for anthraquinone 2-carboxlic acid and rhein in vivo [27,69]. Based on the broad spectrum of bioactivities of quinones, it would be interesting to further characterize the biological properties of 1,6-dihydro 8-propylanthraquinone.

### 4.3. Relevance of 1,6-Dihydro 8-Propylanthraquinone in Nature and Drug Discovery

The antibacterial activity of 1,6-dihydro 8-propylanthraquinone and its secretion into the medium raises the question of whether it truly is a shunt product of the alnumycin biosynthetic pathway. It was identified as a shunt product of alnumycin synthesis produced by deletion mutants lacking either the two-component mono-oxygenase system and flavin reductase or Aln4 and Aln5 with unknown function in *Streptomyces* sp. CM020 [19,23]. The environmental isolate CS 39 produces both compounds simultaneously. Alnumycin biosynthesis is known to be initiated with a butyryl starter unit [70,71,72]; the polyketide chain is then synthesized and later reduced, aromatized, and cyclized to form a bicyclic intermediate that is found in many benzoisochromanequinone pathways [73]. Starting from this intermediate, 1,6-dihydro 8-propylanthraquinone and K1115 were predicted to be produced as shunt products through non-enzymatic side reactions due to the lack of downstream enzymes of the biosynthetic pathway in *Streptomyces* sp. CM020 BGC [23]. In CS 39, the enzymatic step forming the *p*-quinone is a bottleneck of the alnumycin biosynthetic pathway; thus, shunt products can form non-enzymatically during this step.

For drug discovery, 1,6-dihydro 8-propylanthraquinone may be of advantage compared to alnumycin: the structure is comparably simple and, thus, more readily amenable to chemical modification and total synthesis [74]. This fact could allow compound optimization and structure–activity relationship studies, which would further enable the identification of the molecular target [75,76,77].

## 5. Conclusions

We presented an activity and mass spectrometry-guided purification strategy for alnumycin and 1,6-dihydro 8-propylanthraquinone that can be adapted to the purification of other active substances from crude extracts. After confirming the identity of both compounds using NMR analysis, we found 1,6-dihydro 8-propylanthraquinone to have antibacterial activity against *E. coli* Δ*tolC* and gram-positive bacteria. We did not find any evidence of gyrase inhibition contributing to the antibacterial mechanism of either compound, making both alnumycin and 1,6-dihydro 8-propylanthraquinone interesting subjects for further mechanistic investigations.

## Figures and Tables

**Table 1:** Minimal inhibitory concentration (MIC) of alnumycin and 1,6-dihydro 8 propyl-anthraquinone. MIC of purified compounds was tested against *E. coli* Δ*tolC*, *E. coli* DSM 30083, *A. baumannii* DSM 30007, *B. subtilis* 168, *S. aureus* DSM 20231, and *S. aureus* Mu50 in biological triplicates (*n* = 3). OD_600_ values and standard deviations are given in Tables S9 and S10.

Compound	*E. coli* Δ*tolC*	*E. coli* DSM 30083	*A. baumannii* DSM 30007	*B. subtilis* 168	*S. aureus* DSM 20231	*S. aureus* Mu50
Alnumycin	100 µg/mL	>100 µg/mL	>100 µg/mL	8 µg/mL	10 µg/mL	8 µg/mL
1,6-dihydro 8-propylanthraquinone	10 µg/mL	>100 µg/mL	>100 µg/mL	10 µg/mL	10 µg/mL	8 µg/mL

## Data Availability

All data obtained are given in this manuscript or the Supplementary Information.

## Supplementary Materials

The following supporting information can be downloaded via this webpage: https://www.mdpi.com/article/10.3390/antibiotics12071116/s1. Table S1: 16s rDNA similarity values d5 [%] for CS39 in comparison to other genomes of streptomycetal origin; Table S2: alnumycin and 1,6-dihydro 8-propylanthraquinone levels in various production media; Table S3: growth inhibition assay for flash chromatography fractions; Table S4: alnumycin and 1,6-dihydro 8-propylanthraquinone in flash chromatography fractions; Table S5: growth inhibition for preparative HPLC fractions of the flash chromatography fraction 7; Table S6: growth inhibition for preparative HPLC fractions of the flash chromatography fraction 10; Table S7: ^1^H and ^13^C shifts of alnumycin; Table S8: ^1^H and ^13^C shifts of 1,6-dihydro 8-propylanthraquinone; Table S9: MICs of alnumycin; Table S10: MICs of 1,6-dihydro 8-propylanthraquinone. Figure S1: BLASTN analysis of alnumycin BGC of CS 39 and *Streptomyces* sp. CM020.; Figure S2: BLASTX analysis of alnumycin BGC of CS 39 and *Streptomyces* sp. CM020.; Figure S3: ^1^H NMR spectrum of alnumycin in CDCl_3_ (400 MHz); Figure S4: ^13^C NMR spectrum of alnumycin in CDCl_3_ (400 MHz); Figure S5: ^13^C-DEPT NMR spectrum of alnumycin in CDCl_3_ (400 MHz); Figure S6: COSY of alnumycin in CDCl_3_ (400 MHz); Figure S7: HSQC of alnumycin in CDCl_3_ (400 MHz); Figure S8: HMBC of alnumycin in CDCl_3_ (400 MHz); Figure S9: ^1^H NMR spectrum of 1,6-dihydro 8-propylanthraquinone in acetone-d6 (600 MHz); Figure S10: ^13^C NMR spectrum of 1,6-dihydro 8-propylanthraquinone in acetone-d6 (600 MHz); Figure S11: COSY of 1,6-dihydro 8-propylanthraquinone in acetone-d6 (600 MHz); Figure S12: HSQC of 1,6-dihydro 8-propylanthraquinone in acetone-d6 (600 MHz); Figure S13: HMBC of 1,6-dihydro 8-propylanthraquinone in acetone-d6 (600 MHz); Figure S14: gyrase inhibition assays. Reference [52] is cited in the supplementary materials.
