# Supplementary material for: Characterization of the Antibacterial Activity of Quinone-Based Compounds Originating from the Alnumycin Biosynthetic Gene Cluster of a Streptomyces Isolate
Source: Antibiotics (Basel). 2023 Jun 28;12(7):1116. doi: 10.3390/antibiotics12071116 (PMC10376017; doi:10.3390/antibiotics12071116)
Supplement: Supplementary file 1 [file antibiotics-12-01116-s001.zip › antibiotics-2443552-supplementary.pdf]

Supplementary Materials

Characterization of the antibacterial activity of quinone-based compounds originating from the alnumycin biosynthetic gene cluster of a *Streptomyces* isolate

Leonie Sagurna[1], Sascha Heinrich[1], Lara-Sophie Kaufmann[1], Christian Rückert-Reed[2], Tobias Busche[2], Alexander Wolf[3], Jan Eickhoff[3], Bert Klebl[3], Jörn Kalinowski[2], Julia E. Bandow[1,*]

[1]Applied Microbiology, Faculty of Biology and Biotechnology, Ruhr University Bochum, 44780 Bochum, Germany
[2]Technology Platform Genomics, Center for Biotechnology, Bielefeld University, 33594 Bielefeld, Germany
[3]Lead Discovery Center GmbH, 44227 Dortmund, Germany
* Correspondence: julia.bandow@rub.de , Tel: +49-234-3223102

Table of Contents

Pages 3 Figure S1. BLASTN analysis of the alnumycin BGCs of CS 39 and *Streptomyces sp.* CM020.

Page 47 Figure S2. BLASTX analysis of the alnumycin BGCs of CS 39 and *Streptomyces sp.* CM020.

Page 61 Figure S3. ^{1}H NMR spectrum of alnumycin in $CDCl_3$ (400 MHz).

Page 61 Figure S4. ^{13}C NMR spectrum of alnumycin in $CDCl_3$ (400 MHz).

Page 62 Figure S5. ^{13}C-DEPT NMR spectrum of alnumycin in $CDCl_3$ (400 MHz).

Page 62 Figure S6. COSY of alnumycin in $CDCl_3$ (400 MHz).

Page 63 Figure S7. HSQC of alnumycin in $CDCl_3$ (400 MHz).

Page 63 Figure S8. HMBC of alnumycin in $CDCl_3$ (400 MHz).

Page 64 Figure S9. ^{1}H NMR spectrum of 1,6-dihydro 8-propylanthraquinone in acetone-d6 (600 MHz).

Page 64 Figure S10. ^{13}C NMR spectrum of 1,6-dihydro 8-propylanthraquinone in acetone-d6 (600 MHz).

Page 65 Figure S11. COSY of 1,6-dihydro 8-propylanthraquinone in acetone-d6 (600 MHz).

Page 65 Figure S12. HSQC of 1,6-dihydro 8-propylanthraquinone in acetone-d6 (600 MHz).

Page 66 Figure S13. HMBC of 1,6-dihydro 8-propylanthraquinone in acetone-d6 (600 MHz).

Page 67 Figure S14. Gyrase inhibition assays.

Page 68 Table S1. 16s rDNA similarity values d5 [%] for CS39 in comparison to other genomes of streptomycetal origin.

Page 68 Table S2. Alnumycin and 1,6-dihydro 8-propylanthraquinone levels in various production media.

Page 69 Table S3. Growth inhibition assay for flash chromatography fractions.

Page 71 Table S4. Alnumycin and 1,6-dihydro 8-propylanthraquinone in flash chromatography fractions.

Page 72 Table S5. Growth inhibition for preparative HPLC fractions of flash chromatography fraction 7.

Page 74 Table S6. Growth inhibition for preparative HPLC fractions of flash chromatography fraction 10.

Page 76 Table S7. ^{1}H and ^{13}C shifts of alnumycin.

Page 77 Table S8. ^{1}H and ^{13}C shifts of 1,6-dihydro 8-propylanthraquinone.

Page 78 Table S9. MICs of alnumycin.

Page 79 Table S10. MICs of 1,6-dihydro 8-propylanthraquinone.

Page 79 References

Query: CS39 Alnumycin BGC (30.779 bp), Sequence ID: OQ633075 Length: 30779

Sbjct: Streptomyces sp. CM020 alnumycin gene cluster, Sequence ID: EU852062.1 Length: 31030

Range 1: 9293 to 20038

Score:17699 bits(9584), Expect:0.0,

Identities:10419/10812(96%), Gaps:98/10812(0%), Strand: Plus/Minus

```
Query  10883  CCCCGATGCCCCGGACGCCCTCGACCGATCCTCGATCATGACGGACCACAGTGTGCGGCA  10942

Sbjct  20038  ...G..C.......G........................CG...................  19979


Query  10943  CAGGACCTTCAAGACCCTGGCGGCCTTCAGAGCCTCCAGGACCTTCAGGAAAGGAACGTG  11002

Sbjct  19978  ....................-.......................................  19920


Query  11003  CCCATGCTCTCCATACGTTCGCGGCGCCTCGTCCGGGGCGGCCTCGGGACACTCGTCGGC  11062

Sbjct  19919  ................C...........................................  19860


Query  11063  GTGCTCGTGCTCCCGCTGATCGGCTCGGCGCCCGCCGGGGCCCAGCAGGACGCGAGAGGG  11122

Sbjct  19859  ............................................................  19800


Query  11123  TCGGCCAAGGCGGTCGCCATGACGGAACTCGACTTCCTGCTCGGCGACTACACCTGCGCG  11182

Sbjct  19799  .....G.........T............................................  19740


Query  11183  TACACCGACCTCACCCTCGAGGAGCCCACCACGGTCACCCTCAACTGGGACACCAAGAAG  11242

Sbjct  19739  .................T..........................................  19680


Query  11243  ACCCTTGAGGGCAAGTTCTACGAGATGCACCTCAAGAGCCCCGCCTTCGAGGGGCGCTGG  11302

Sbjct  19679  ............................................................  19620


Query  11303  GTCTTCGGCCTGAACACGGTCGACAACCGGTACACCTCGTTCTACTGGGACACCTGGGGC  11362

Sbjct  19619  ......................................C.....................  19560


Query  11363  AACACCGGAACCGCGTCCTCCGTCGGCTGGAAGCGCGACATGCTGCGCTTCCAGGGCCCG  11422

Sbjct  19559  .....................CC..............G......................  19500


Query  11423  TACATCACGCCCGGCGGGCACGCGGACAGCAAGGACGAGTTCCGGGTGGTCAACAGCGAC  11482

Sbjct  19499  ............................................................  19440
```

```
Query  11483  CGGTTCACCGACGACGCCTTCATCCGGTTCGAGGGCCAGCCGTGGAAACAGATCAGCCAT  11542

Sbjct  19439  ...................................T........................  19380


Query  11543  GTGGACTGCCGCCGGTCCTGAACCGCACGCGGCGCCCAGGCCGGCCCCGAACCGCACCGA  11602

Sbjct  19379  ......................................C.....................  19320


Query  11603  GCGCCGCACAAAGCCCCGCACCCGACTCCCGGGCCGGGGCTTTTACATTCCGGCCAACAG  11662

Sbjct  19319  .......GG...................A...............................  19260


Query  11663  GTCGATAAATGCGCGTTACAAAAGGTTGCCCGGCCCGCCGGAGGTGTGCCAGAATTCACA  11722

Sbjct  19259  ..................................A.AT..AC..................  19200


Query  11723  TTACCTGGGTGCGATGCCCGGAC-CGGCATCGCACAAAATTGGCTGGTTCTCGCCCCACC  11781

Sbjct  19199  .C.....A.C..A.CAG..-...T...G.........T.......T...T..........  19141


Query  11782  TTTTCCTCCCCGAGCCGATCATCCGCGCCGGGTATGAGGAATTCATAGGCACAGCCATGG  11841

Sbjct  19140  ............T.......GC..CT..................................  19081


Query  11842  CGGATTCACTATGGCGATCCGAAGTCCTCTGCGAGGCTCCCAATTGGAATTTTCCGACAG  11901

Sbjct  19080  .............T..............................................  19021


Query  11902  GGGGCCTGGATATGGAAATCGAAGTTTTAGGACCGCTCGACATCCGTCTGGACGGCACGT  11961

Sbjct  19020  ..................................A.........................  18961


Query  11962  CGATCGTGCCGAGCGCGGGCAAGCCTCGGCAGATACTGGCACTCCTCGCCCTGCGAGCCG  12021

Sbjct  18960  ......................A..........................T..........  18901


Query  12022  GACGGATCGTACCCGTACCGGTTCTCATGGAGGAGATCTGGGGTGACCGAATACCCCGGA  12081

Sbjct  18900  ............................................................  18841


Query  12082  GCGCACAGACCACCTTGCAGACCTACATCCTTCAACTCCGCCGCAGGATCAGCGCAGCCC  12141

Sbjct  18840  ............................................................  18781


Query  12142  GGCCGGACGTCCGGCGACCGACCGCGAAGGACGTACTGTCCACCCGGTTCGGCGGATACC  12201

Sbjct  18780  .T...........A..............................................  18721
```

```
Query  12202  TGTTGTCCGAACCGGTGCTCAGCAGTGATGTCGGAACGTTCCAACGGCTCACGGCGGAAG  12261

Sbjct  18720  .........................C.................G..A.............  18661


Query  12262  GGAGCGCCGCACTGGAGAGGGGCGAGGCCGGCCTGGCCGCCGACGTGCTCGGCAGGGCCC  12321

Sbjct  18660  ...........................................T................  18601


Query  12322  TGAGCCTGTGGCACGGCTCGGCCCTCATCGACGTACCGACGGGGCATGTCCTGGACACCG  12381

Sbjct  18600  ..G...................G.....................................  18541


Query  12382  AGATCCTCGGCATCGAGGAGGCCAGGGCGCGGGCGCTCGAACTGCGGATCGAAGCCGATC  12441

Sbjct  18540  ....T...............................................G.......  18481


Query  12442  TGCGGCTCGGCAGGCACGCCGAACTCCTCGGCGAGCTGCGGATGCTGGTCGCCCAGCATC  12501

Sbjct  18480  ....A............................................A..........  18421


Query  12502  CGATGCACGAGAGCTTCCATGCTCAGCTGATGATCGCCCTGTGCCGTTCCGGGCACACCT  12561

Sbjct  18420  ...................C..G..........................G..........  18361


Query  12562  GGCGGGCCCTCGACGTCTATCAGCAACTGCGCTCGGCGCTCGTCGGGGAACTGGGCGTCG  12621

Sbjct  18360  ............................................................  18301


Query  12622  AACCGTCCGACCGGATACAGCGGCTGCACCAGCAGGTCCTGGGCGGAGGCCTGGACAAAC  12681

Sbjct  18300  ................C...........................................  18241


Query  12682  CGCGAAGCACCTACGTGGAGCGCGATCTCGCCCTCGAACTGCGGTAACTCGCGGTTCGAA  12741

Sbjct  18240  .............T..................................G......C....  18181


Query  12742  GCTCGGTGTTGAACCCTGCGCGCCGGTGAGAACCGGTCGCA-----------C----G-C  12785

Sbjct  18180  ..A......................................AGCACGACGAG.GGGG.T.  18121


Query  12786  ---A-CA-GA--AGCGGGGCCGTCGTAGTACTG-ACGGCCCCGCTCCCTCATGTCCCGTG  12837

Sbjct  18120  GTA.G..C..CG............CCG..T...G............GG..G.........  18061


Query  12838  ATCCGCTCCCCCGGTCAGGGCCTGCGGATCACCTTCGTCCAGTCCGCCCTGATCTCGGAG  12897

Sbjct  18060  ............................................................  18001
```

```
Query  12898  CCCTGGCCGAAGAGCCGGTTCTCGACCTTGCTCTCGGGGTCGAAGAACTTCTGTTCCACC  12957

Sbjct  18000  ......................................................G.....  17941


Query  12958  GGCTCACCGCGCCGCGGATCGAAGTAGAAGCCCTCCTTGTGCTTGAGCCGCACCAGCGCC  13017

Sbjct  17940  ................................G...........................  17881


Query  13018  CCGCCGTCGGCCGCGCCCGGCTTGGCCCCGTCCAGCAGGCTGCCCAGCCCCATGCCGCAG  13077

Sbjct  17880  ......................................A.....................  17821


Query  13078  GTGATCTGGTCGAGCTCGACCCGCTCCTCCGTGCCGCCGTGGTCGAGCGTCATGTACTTG  13137

Sbjct  17820  ..............T..........................A..................  17761


Query  13138  CGGTCGAAGGCGCGCGTGCCGATCTTGTCGGTCTGGAGCTTCTGCCGGCCGTCCACGAAC  13197

Sbjct  17760  ............................................................  17701


Query  13198  CAGGTCACGCGCTTGCCGCTCTGGTCCAGCCGGACCTCCAGTTCGTGCATCGCCCCCGGC  13257

Sbjct  17700  ............................................................  17641


Query  13258  GCCGTCTTCGCCAGCGGGATCGCGTAACCGTAGGAGGCGTAGTCCTCCGTGTCGCTCGGC  13317

Sbjct  17640  ........G.................G.....C...........................  17581


Query  13318  AGACGCTCGTAGATGGCGTAGATGGTGTCGTTCGTGACCGAGAAGTCGGCGATCGCCCGG  13377

Sbjct  17580  ..G.........................................................  17521


Query  13378  GACTCGAAGTCGGCGGTGATCAGCGCGATCGAGGCGAGCCGGATGTCGGACGCCGGGTCC  13437

Sbjct  17520  ............................................................  17461


Query  13438  TTGACGGTGCTCCCGAACGGGTTCTCGGTGCCGAAGGTCCGGCCCGCCACCTTGTGCACA  13497

Sbjct  17460  .......................T....................................  17401


Query  13498  CAGCTGATGGAGCCGGTGTCCGGCACCTCGAAGCCCGGGACGTTCTCGGCGGTGAACCGG  13557

Sbjct  17400  ............................................................  17341


Query  13558  TTGGGGAAGGCGATCCACTTGATGTGGTCGTCCGAGTCCGCGCCGCCGTCAGGTCCGGTG  13617

Sbjct  17340  ..................................................C.........  17281
```

```
Query  13618  CTGCGGGCGTAGGCCGGGTCGCCCGTCTTCGGGTTGACGCCGGTGGGCACCACGTTGAGC  13677

Sbjct  17280  ............................................................  17221

Query  13678  ACGCCGCCCGGCGTGGTGACGACGGCGTCCCCGGTCGGCCACTCCGCATCGCCCTGGAGC  13737

Sbjct  17220  ..C.........................................................  17161

Query  13738  AGCCAGTTGCCGTTCTCACCGGTGGTGAAGCCGTTGCCGTACGTGTCCTGGAACAGGACG  13797

Sbjct  17160  .................................G..........................  17101

Query  13798  GTcccgccgcccgcccccgcccgcgacgccgtcggcgcggactgcgcggtcgccggcagc  13857

Sbjct  17100  .............A.......T......................................  17041

Query  13858  agcgccACCACGGCCGCCGCGGCCACCACGGCGTACGCAACCTTGTGTCTCATCGTTCCC  13917

Sbjct  17040  ............................................................  16981

Query  13918  CTTGCCACAACTACCCCCTGTTTCAGCGGTTTCCGGTTCCGCGACCGTGCCGGAAGGCAC  13977

Sbjct  16980  ............................................................  16921

Query  13978  CGGAGTTTCTCTTGAGGACCGCTGCGGCACGGGCTTCGACAGCTCCTCGACCGGGGCTCC  14037

Sbjct  16920  ............................................................  16861

Query  14038  AGCGGGGGCGGGGAGGGTGACCAGGCGAGTCAGTTCCCGCACCTCAGGAAGGACACCCCG  14097

Sbjct  16860  ...................G..C.....................G...............  16801

Query  14098  GACATGGAACGACAGCCCGACCAGCTGCTAGAGGTCAGCGACGAGATCGCCACGGCGCTC  14157

Sbjct  16800  .....................................................C......  16741

Query  14158  GCGGAACGGCGTCCGGTGGTGGCGCTGGAGTCCTCGCTCATCACCACCGACCCCTCGTCC  14217

Sbjct  16740  ............................................................  16681

Query  14218  GAGACCGCCTCACTGATCGAGAAGGCGGTACGGGGCGCCGGGGCCGTACCCGCCACCATC  14277

Sbjct  16680  ............................................................  16621

Query  14278  GGGATCGCCGGCGGGAAACTCGTCGTCGGTCTGACCGACTCCCTCATCGAACGCTTCGCC  14337

Sbjct  16620  ............................................................  16561
```

```
Query  14338  TCCACCAAGGGCATTCCGAAGATCAgcgcgcgggacatcggcggcgcgctggccggcggc  14397

Sbjct  16560  ............................................................  16501


Query  14398  ggcctcggggcgacgacggtggccggcACCATCGTGATCGCCGAACGGGCCGGCATCCAG  14457

Sbjct  16500  ............................................G...............  16441


Query  14458  GTCTTCACCACGGCGGGCATCGGCGGTGTGCACCGCAGGGGCGAGGACACCCTCGACATC  14517

Sbjct  16440  ............................................................  16381


Query  14518  TCCCCCGACCTGCTCCAGTTCCGCAAAACGAAGATGACCGTGGTCTCCGGCGGCGCGAAG  14577

Sbjct  16380  ......................................G.....................  16321


Query  14578  AGCATCCTGGACCACCGGCTGACCGCCGAGTACCTGGAGACGGCCGGTGTCCCCGTGTAC  14637

Sbjct  16320  ............................................................  16261


Query  14638  GGGTACCGCACCGACAAACTCGCGGCCTTCGTGGTGCGCGAGGCCGATGTGCCCGTGACC  14697

Sbjct  16260  .........................................A..................  16201


Query  14698  CGCATGGACGATCTGCACACCGCCGCGCGCGCCGCCGAGGCCCACTGGCAGGTCAACGGA  14757

Sbjct  16200  .........................................G.................C  16141


Query  14758  CCCGGCACGGTGCTGCTCACCAGTCCCATCGACGAGCAGGACGCGGTGGACGAGGCGATC  14817

Sbjct  16140  ............................................................  16081


Query  14818  GTCGAGGCCGCCATCGCCGAGGCCCTCGCCCAGTGCGACCAGGAGGGCATCGTGGGCAAC  14877

Sbjct  16080  ............................................................  16021


Query  14878  GCGGTCAGCCCGTACCTGATGAAGGCGCTCGCCAGGGCCTCCGGCGGCATGCTGCCCAAG  14937

Sbjct  16020  ............................................................  15961


Query  14938  GCCGGCCGTTCCCTGCTGCTCAGCACCGCCCGGGTCGCGGGAGAGTTCTCCGCCGCCCTG  14997

Sbjct  15960  .....G..C.......................C...........................  15901


Query  14998  AGCGCCGTACAGGCCGAGCGGTGACCGGCGCCCCCGCCGCCGCGGACCGTGGTGTGATCC  15057

Sbjct  15900  ........................G...............G..A................  15841
```

```
Query  15058  TCGACCTCGACGGCACACTCGCGGACACCCCCGCCGCCATCGCCACCATCACCGCCGAGG  15117
Sbjct  15840  ............................................................  15781

Query  15118  TCCTGGCCGCGATGGGCACGGCGGTGTCCCGAGGGGCGATCCTCTCCACGGTCGGCCGCC  15177
Sbjct  15780  .......G............................................G.  15721

Query  15178  CGCTGCCGGCCTCTCTCGCCGGGCTGCTGGGCGTTCCCGTGGAGGACCCGCGGGTGGCCG  15237
Sbjct  15720  ......................T.....................................  15661

Query  15238  AGGCGACCGAGGAGTACGGGCGGCGGTTCGGCGCCCATGTGCGGGCGGCCGGGCCGCGGT  15297
Sbjct  15660  ............................................................  15601

Query  15298  TGCTGTATCCCGGGGTACTCGAAGGGCTGGACCGGCTGTCCGCCGCCGGTTTCCGGCTGG  15357
Sbjct  15600  ................C...........................................  15541

Query  15358  CCATGGCGACCTCGAAGGTCGAGAAGGCCGCGCGCGCCATCGCCGAACTCACCGGTCTGG  15417
Sbjct  15540  ............................................................  15481

Query  15418  ACACCCGGCTGACGGTCATCGCGGGCGACGACAGCGTGGAGCGCGGCAAGCCGCACCCCG  15477
Sbjct  15480  ............................................................  15421

Query  15478  ACATGGCCCTGCATGTCGCCAAGGGGCTCGGGCTCGCTCCGGAGCGGTGCGTGGTGATCG  15537
Sbjct  15420  ...................CG..........A..C........................  15361

Query  15538  GAGACGGGGTGCCCGACGCCGAGATGGGGCGGGCCGCCGGCATGACGGTCATCGGGGTCT  15597
Sbjct  15360  .T.....................................T..............T....  15301

Query  15598  CGTACGGCGTGTCCGGCCCCGACGAACTGATGCGGGCGGGAGCGGACACGGTCGTGGACT  15657
Sbjct  15300  ............................................................  15241

Query  15658  CCTTCCCCGCGGCGGTCACCGCCGTACTGGACGGACACCTGTGAACGCGCCCCGCACGCC  15717
Sbjct  15240  ......................................C...............C....  15181

Query  15718  CGGCACTTCGCGCACGGACGCACCGCTGGACACCCCGAACACCCCGAACACCCCTAGGAA  15777
Sbjct  15180  .C....CC...................................---------....  15130
```

```
Query  15778  AGGAGCGGTTCCGATGCCGCGCAGAACGTTCGTCTCCCGGCACGGCCTGCTGCTCGCCGC  15837

Sbjct  15129  ........C.T.................................................  15070


Query  15838  CGCCGCACTGGGCACCGTCATGACGACGCCGGGGACCTCGACCGCCAAACCCGGCAGCCG  15897

Sbjct  15069  ......G....................A...........C...........G........  15010


Query  15898  TGGCTACCGGATGGTCTGGGACGACTTCGCCGAGGGGTTCCGCACCGAGGGAGAGGGCGC  15957

Sbjct  15009  .................................C.................C........  14950


Query  15958  CCGGTGGTTCCACGTGGCGGGCGGCCCCTACCGGGCCGACGACGGCATCGTCACCACCCG  16017

Sbjct  14949  ........................T........T..................G.......  14890


Query  16018  CCCGGGCGAGCTGTCCGTGCGGGCACGGGGCAGCCACCCCGCGACCGGCGAGCCTGCGTT  16077

Sbjct  14889  ..G........................T.....T....................G.....  14830


Query  16078  CACGCAGACCATCCCCGCCGAGAACCCGGTCGGCATGCCGGGCTCCGGGGACCACGCCAA  16137

Sbjct  14829  .............................................A..............  14770


Query  16138  GTTCATCGCGTACACCAGTCACACGGCCTCCAGCGGCCACCCGGGGTTCGACGCCCACGA  16197

Sbjct  14769  ............................................................  14710


Query  16198  AGGCTATGAACTCCTCTTCGAGACGCGGCTCTCCGGCCGGACCTACGGCACCGCGGACCA  16257

Sbjct  14709  G.....C..G..........................TA......................  14650


Query  16258  CCCCTTCGGGGACGCGGTGCGGGATCCGGAGGACGATCTGCGGCTGGCCTCCGCGATGAT  16317

Sbjct  14649  ............................................................  14590


Query  16318  GCTGACCACCGACCCGGAGACCTCCGTCTCCTTCGACTTCGTGGTCACCAACAAGCGGAT  16377

Sbjct  14589  ............................................................  14530


Query  16378  CTACGCCTGGTACGGGCGCCCCACCTTCCTGCGCGGCCAGTTGGGCGACTACGCCTCGTT  16437

Sbjct  14529  ............................................................  14470


Query  16438  CGCGCACACCGTGCCGCTGGTCGCGCGGCGGCCCGGCGACAGCCACGACTTCGGCATCGC  16497

Sbjct  14469  .........T...............A..AA..............................  14410
```

```
Query  16498  CTACGACCGCGCCGCCGGGGTGGTGCGCTGGCTCATCGACGGCGAGGAGCACTTCCGGGT  16557

Sbjct  14409  ..................T.........................................  14350


Query  16558  GGACCGCATCGGACACCGCCTCGACCGGTCCACCGCGACCCTCGACGAGGGCGGCGAGGA  16617

Sbjct  14349  ..................G.......................G.................  14290


Query  16618  GACGCTGGTCAGGCCGAGGCAGCTCAACGCCGGCCTTGGACTGCTGACCCTGCTGGACGC  16677

Sbjct  14289  .................................G..G.......................  14230


Query  16678  GTCCTGGCCCACCGACAAGGGCCTCGTACGGCTCTCCGCGCGCAAGCACACCACGTACTA  16737

Sbjct  14229  ............................................................  14170


Query  16738  CCGGCCCTCGGTCGGTGCGCCGCAGGAGCAGACCTTCGCCGACGAGGACAGCACCGACGC  16797

Sbjct  14169  ..................C.........................................  14110


Query  16798  CGGGCGGCTCTTCGGACAGGGCGCGGCCGTACGGCTCGGCTCCTACCGCGTGACCAGCAG  16857

Sbjct  14109  ...............G..............G.............................  14050


Query  16858  GCGCTCGCGGTGACGCCGGAGGAGGTCGTCGGAGTGTGGCGGCTGGCCTCGTACACGGAG  16917

Sbjct  14049  ............................................................  13990


Query  16918  GTGGGCGAGGACGGGGGCACGGTGGCGGGGCCGTTGGGCGAGGCGCCGACCGGGCTGCTG  16977

Sbjct  13989  ................................................G...........  13930


Query  16978  ATCTACACCGCGGACGGCCATGTCGCGGTGAGCATGATGAAGACCGGGGACGCCCCGGCC  17037

Sbjct  13929  .................G..........................................  13870


Query  17038  CTGGAGACCTACATGGGCTACTCCGGGCAGTGGCGGCTGGCCGGGGACCGGATGACGCAC  17097

Sbjct  13869  .......................G....................................  13810


Query  17098  CGGGTCCAGGTCAGCGCCCACCCGCGGATGGCGGGCACCGAACAGATACGCCGCGTCGCG  17157

Sbjct  13809  ....................T....................G..................  13750


Query  17158  CTCGACGGCGAGACGCTGTCGCTGCGCGGCACGGCCGTGACACCGGTGGGGGCCGCGCG  17217

Sbjct  13749  ...........A................................................  13690
```

```
Query  17218  CCGGAGCGCGTACTCACCTGGCGGCGCGCGAACCCGGACGGCATCGCAGCCGACGGCATC  17277

Sbjct  13689  ....-----------.-.--..-.TA.T.--...T..-...---...-.-...-A..--.  13655


Query  17278  GCGACCGACCGCATCGCAGCCGACGGCACGGCATCCGGCAGCACGGCGATCGACAGCACA  17337

Sbjct  13654  .-..---.-G.......G..............................T.....G....-  13601


Query  17338  GCTTTCGACAACACGGCTTTCGACAACACGGCTTTCGACAGCACGGCTTTCGACAGCACG  17397

Sbjct  13600  .-----.-G.T.-...----..---G......G...........A...............  13555


Query  17398  GCGTCCGACGAGACGGCGAAGCAGCAGGAGAAGGAGACGCACTGATGCCCAACTACGTGG  17457

Sbjct  13554  ..................G.A.......................................  13495


Query  17458  ACGCGCCCGAGGGTTGGTGGCGGGAGTTCATCATGGCCGATCCGCCCAGGACCGCGAAGG  17517

Sbjct  13494  ............................................................  13435


Query  17518  CCTCGGTGGTCCGCAAGGACGGCAGCCCTCATGTCGTCCCGGTCGGAGTCATCATGGACG  17577

Sbjct  13434  .........................T..C...............................  13375


Query  17578  GCGACGACATCATCTACACCTGCCAGAAGGACAGCGTGAAGGGCCGCTCCCTGCAACGGG  17637

Sbjct  13374  ...................G........................................  13315


Query  17638  ACGGCAGGATCGCGATGTTGTGGGACGACGAGCGCCCGCCGTTCTCGTTCGTGCTGGTGC  17697

Sbjct  13314  .................C..........................................  13255


Query  17698  GCGGGCGGGCGACGCTCAGCGAGGACATCGACGAACTGCGCGCCTGGACCGCCCGGATCG  17757

Sbjct  13254  ..........................C.................................  13195


Query  17758  GCGGCCGGTACCACGGCAAGCGGCGCGAGGAGGAGTTCTCCGAGCGGTTCACCATCCCCA  17817

Sbjct  13194  ............................................................  13135


Query  17818  ACGGCGTCGTGGTCCGGGTGAAGGTGGAACAGATCGTCGCCAAGGTGAACCTGTCGGAGA  17877

Sbjct  13134  .................................................T..........  13075


Query  17878  CCGTGAACGTGAAGGACTCTTAGCCCT-CCTGGGCCTCTCCCGGGGCCTGTGGGGTGACA  17936

Sbjct  13074  .................G.G.......A..G..............-..C.G.C......  13016
```

```
Query  17937  CAACAGTGCGGCGGAGCGGGCCGGTTGGCCGCTCCGCCGCACTGTTGCGTCAGGCACCGT  17996

Sbjct  13015  .................C.............................GT..........C  12956


Query  17997  ACTTCCGG---TGGAGCGCTCAGGCCGCCCCGTACCGGTCGTCGGCCGGTACCCGTCGCG  18053

Sbjct  12955  ...C....AGGA.............G.......................G..........  12896


Query  18054  CGGTCGTCCGCAGGTCGTCGACCTGCTGCCAGCGGTCGCCGTAGATGGCGTCCCGCATGG  18113

Sbjct  12895  ............................................................  12836


Query  18114  CCGGGAAGCGCAGGAAGTCGTGCGGGAAGCCCGGCGCCACACTGCTGACCCGTTCCAGGC  18173

Sbjct  12835  ...................................T....G.G............G....  12776


Query  18174  CCGCCAGCCAGTCGTCCTCCAGCACCGCTTCGACGGCCCCCAGGTTGTCGGCGAGCTGCT  18233

Sbjct  12775  ..................G......G..................................  12716


Query  18234  CGGGCTTCGTGGCGCCGAGGATCGGCACCACGTTTGCGGGCCGGGACAGCAGCCAGGCCA  18293

Sbjct  12715  .......G....................G...............................  12656


Query  18294  GCGCCACCTGGGCGGGGCTGATTCCGGCGGCCTCGGCGACCCGTACGGTCTCCTCCACGA  18353

Sbjct  12655  .....................C.....................C...........G....  12596


Query  18354  CGTCGTCGCCGGGACCGGTGCCGCCCTGCCCCCAGGCCACGTGGTCCAGCCGCCCCTCCT  18413

Sbjct  12595  .............G...................................G..........  12536


Query  18414  CGGACCGCAGATACTTGCCGGTGAGCCGCCCGTCGGCCAGCGGGCCCCAGGCGAACACCG  18473

Sbjct  12535  ..........G.................................................  12476


Query  18474  GCAGGTCGAAGGCGTGGGCCATCGGGAGGAGTTCACGCTCGACGGTGCGCTCCAGCAGGT  18533

Sbjct  12475  .A.......................C..................................  12416


Query  18534  TGTACCTGATCTGCAGTCCCGCGAAGGGCGACCAGTCGCGCAGTTCGGCCAGGGTGTTGG  18593

Sbjct  12415  .....................................A......................  12356


Query  18594  CCTGGGCCACCTCCCAGGCGGGCCAGTCGGACACACCGACGTAGAGCACCTTGCCCGCGC  18653

Sbjct  12355  ................C.............................G.............  12296
```

```
Query  18654  GCACCTGGTCGTCCAGCGCGCGCATCACCTCGGGCACCGGGGTGAGGGTGTCGCGGGCGT  18713

Sbjct  12295  ...................C.T..............................T.......  12236


Query  18714  GCACCCACAGCATGTCGATGTGGTCCGTGCCGAGCCGACGCAGGCTCGCCTCCAGGGAAC  18773

Sbjct  12235  ................C.C..................G......................  12176


Query  18774  GCACCAGGTTCTTCCGGTGGTTGCCCGCGGAGTTGAGGTCACCCGGCCGCGTCTGCATCG  18833

Sbjct  12175  .............G....................C...............C.........  12116


Query  18834  TGTACTTGGTGCTGAGGACGAACTCGTCGCGGCGGCCGGCGAGCAGCTTGCCGAGCGTCG  18893

Sbjct  12115  ..................................T.........................  12056


Query  18894  TCTCCGCCGATCCGTCGCCGTACTCGTCGGCGGTGTCGATGAAGTTGCCGCCGGCCTCGG  18953

Sbjct  12055  ............................................................  11996


Query  18954  CGTACTGGTCGAAGATCCGCGTGCGCGTCTCCTCGGGAAGCTTCCAGTCGTCGCCGAACG  19013

Sbjct  11995  .....................C...T...G.......G....................G.  11936


Query  19014  TCATGGTGCCCAGTGCCAGCCGGGAGATCCGGACTCCGGTCCTGCCGAGAAGCGTGTAGC  19073

Sbjct  11935  .........................T........C.........................  11876


Query  19074  GCACGGGTCAGTCCTTCCGGCTCAGTATGACCAGGGGGATCTTCCGTTCGGTCTGGGCCT  19133

Sbjct  11875  ....A.C.......................................C..A..........  11816


Query  19134  GGTAGGCGGCGAAGGCCTGCACCTCGGCGGCGGCCTCGGCGAAGAGGCGGTCGCGCTCCT  19193

Sbjct  11815  ............................................................  11756


Query  19194  CGCTCCCGGCCGGCACCTCGGTCGCGACGGCCGAGTAGGTCTCCTCGCCGGTCTCGACGG  19253

Sbjct  11755  ............................................................  11696


Query  19254  TCACCCGGGGATTGACCCTGATGTTGTGGAACCAGGCGGGGTGGGTGTCCGCGCCGCCGT  19313

Sbjct  11695  ............................................................  11636


Query  19314  TGCTGCCGAAGATCAGCAGGCTGCCGTCGGACTGGGCGAAGAAGAGGGTGGGCACCGTGC  19373

Sbjct  11635  ......................A.....................................  11576
```

```
Query  19374  GCACCACGCCGGACTTCGCCCCCTTGGTGGTGAGCAGGATGAGCGAACCGCCCTCGAAGG  19433

Sbjct  11575  ....................................................T.......  11516


Query  19434  GACCGCCGACCTTGCCGCCGTTCTGCCGGAACTCCTGCATGACCGGGAGGTTGTAGCTGC  19493

Sbjct  11515  ............................................................  11456


Query  19494  CCGGGTCTCCTTCGGCGGGGGTGGTGGGCGCGGGGTCGAACTGGTCAGCAGCCACGGGGA  19553

Sbjct  11455  ............................................................  11396


Query  19554  CTTCCTCTCGGCGAGACAAACGGAGAACTCTCCACATCAGTACGCGCGCCGAGCCTAAGC  19613

Sbjct  11395  ..........................TT......T...GA....................  11336


Query  19614  GGAGAGACTTCCGTATCGCAAGCAACGACCTCGTACGCCGCTGGAGCAGGGGCGAACTAT  19673

Sbjct  11335  .......................................A....................  11276


Query  19674  GCTCACGGCCATGGCCGCAGCCCCATCCGAACGCGCCGACGCGGTGCGCAACCGACAGAA  19733

Sbjct  11275  ......C.................C...................................  11216


Query  19734  GATCCTGGCCGCGGCGGCCCGTCTGGTGGCCGCCGGAGCGGCCGAGCGGCTCTCCCTCGA  19793

Sbjct  11215  ...............A..........................G.................  11156


Query  19794  CGAGGTCGCCCGGGTGGCGGATGTGGGCGTGGGCACGGTCTACCGCCGCTTCGGCGACCG  19853

Sbjct  11155  .....................C......................................  11096


Query  19854  CGCCGGACTGGTCTTCGCCCTTCTGGAGGAACAGCATCAGCTGTTCCGGGCCCGGGTGGT  19913

Sbjct  11095  ..................G..C..............C.......................  11036


Query  19914  GGAGGGCCCGCCCCCCTTGGGGCCGCGGGCCGACGCCGGTGACCGGCTGCGCGCCTTCCT  19973

Sbjct  11035  .........................G..................................  10976


Query  19974  GCACGCGCTCGTCGACCTCTCCGTCGGCCAGCGGGAACTGCTGCTGCTCGCCGAGTCCAG  20033

Sbjct  10975  ...........................A................................  10916


Query  20034  TTCGCCGCCCGCGCGTTACCTCAGCGCCTCCTACGACTTCCAGCACGCGCATGCCGGCCG  20093

Sbjct  10915  .......................................T...........C........  10856
```

```
Query  20094  GCTGATCGCCGAGCTGCGCCCGGACGCCGACGCGGACTTTCTGGCGGACGCGCTGCTGGC  20153

Sbjct  10855  ............................................................  10796


Query  20154  CCCCTTCGCGCCCAGCCTGATCGACCACCAGAGCCGGGTCAGGGGCTTCTCACCGGAGCG  20213

Sbjct  10795  .........A.....................T............................  10736


Query  20214  GATCAAGGCCGGCTTCGACCAGCTCCTGCGCAGCACGCTGGAGTCGAGGCGGTGACAGCG  20273

Sbjct  10735  .........................G............C.......GC........G...  10676


Query  20274  ACCGCTGTCGAGCCGACCTCCAGCCGCCTTCCAGCGGAGACCGGGACCGTGGCCGACGAC  20333

Sbjct  10675  G..C.................................................G......  10616


Query  20334  GGGTGCGCGAACGCACGCCGCTGACGGCATGGCCGCCTGGAGCGCGACGTCCATGCCCAC  20393

Sbjct  10615  ............................................................  10556


Query  20394  GGCTGCTTCCCCGCACGACGCGGTCCATGGGAGGCGCAAGGATGCTGGACACCGAGGAAC  20453

Sbjct  10555  ............................................................  10496


Query  20454  TGGCCCAACAGGCCGCCGAATCCGCGCGAGGGGCCGAGGAAGCCCGCAAGCTCGACCCCG  20513

Sbjct  10495  ............................................................  10436


Query  20514  ACGTGGTCAAACTGCTGGTCGAGGCCGGCTTCGCCCGGCACTTCGTACCCCGGGAGTGCG  20573

Sbjct  10435  ............................................................  10376


Query  20574  GCGGCACCGAAGGCACCTTCGCGGAGCTCACCGAGGCGGTGGCGCAGGTCGGCGCCGCCT  20633

Sbjct  10375  ..........G................................A.G..............  10316


Query  20634  GCCCCGCCACCGCCTGGTGCGCCTCGCTGGCCGCCAACCTGGGCCGGATGGCGGCGTATC  20693

Sbjct  10315  ............................................................  10256


Query  20694  TGCCCGCCGAGGGCTACCGGGAGGTGTGGGCGCAGGGGCCCGACGCGCTCGTCGTCGGAT  20753

Sbjct  10255  .......A..................................................C.  10196


Query  20754  CCCTCTCCCCCTTCGGGAAGGCCGTCCCGGCGACCGGCGGCTGGACACTGTCCGGGCGCT  20813

Sbjct  10195  ........................C...C...G..............G............  10136
```

```
Query  20814  GGCCCTACATCAGCGCCGTGGCGTACGCGGACTGGATGCTGCTGTGCGGCACGCTGCCCG  20873

Sbjct  10135  ...................................C............C...C...T...  10076


Query  20874  ACGGGGCCGGGCCCCGGGTCTTCGCGGTTCCCCGGGACGAGGTGGAGGTCGTGGAGAGCT  20933

Sbjct  10075  ............................................................  10016


Query  20934  GGGAGAGCGTCGGGATGCGGGCCACCGGCAGCCACACCGTGGTCGCCTCCGACGTGTTCG  20993

Sbjct  10015  .............T..............................................  9956


Query  20994  TTCCCGAGCGGCGCGCCTTCGCCCGCGCCGACCTGCTGACGGGCCGCCCCACGGCGTCCA  21053

Sbjct  9955   .G..........................................................  9896


Query  21054  CGGCGGCCTGTCACACCGTGCCGCTGGAAGCGGCCAACGGTCTCTCCTTCGCCGGGCCGC  21113

Sbjct  9895   .....................T......................................  9836


Query  21114  TCCTTGGCGCCGCCGAGGGCGCGCTCGCCCAGTGGTCGGCGTACGCGGTGGTCAAGGCGC  21173

Sbjct  9835   ............................................................  9776


Query  21174  GCTCCGTACTCCTGCGGCCCCAGGCGCCGGGCCCCAGCCGCGAGGCGCTCGCCGGCGTGC  21233

Sbjct  9775   ..............................................A........T....  9716


Query  21234  TGGCCCACTCGGCCGGGGAGACGGACGCCGCGCGGCTGCTGCTGGAGCGGTGCGCGCAGG  21293

Sbjct  9715   ...............................A...........C..A.............  9656


Query  21294  TCGCCGACCTGGGCGCGGAGGTGACACCCGAGCAGACCCGGCGCAATCTGCGCGACACCG  21353

Sbjct  9655   ...........................T................................  9596


Query  21354  CCCTGTCCGTACGGATGCTGGCCTCCGCGGTCAACCGCCTCGCGGCGAACGGCGGCACCA  21413

Sbjct  9595   .......G....................................................  9536


Query  21414  CGGGCTACGGCGAGAGCGCCCCGCTGCAGCGCTACTGGCGGGACGTCAACACCTCGGCCA  21473

Sbjct  9535   ............................................................  9476


Query  21474  CCCACGTGGCCCTCCAGTTCGAATCGGCGGCACTCGCCTACGCCGAGGGACGACTGTGGA  21533

Sbjct  9475   ...............................G.................C..G.......  9416
```

```
Query  21534  CCACGGAGACCCCGGAGACCACGCAGACCAGCAGCCCGGCAGcggcccggtagcggccac  21593

Sbjct  9415   ...........---------..............T.......................C.  9365


Query  21594  gcggcacggacagagccggccggccccgggagacggggccggccggcT-CTGT-CACGGC  21651

Sbjct  9364   ..AA.G..T.............................T.........T....T......  9305


Query  21652  AG-TTCGCGCG  21662

Sbjct  9304   ..C....G....  9293
```

Range 2: 2842 to 9188

Score:10691 bits(5789), Expect:0.0,

Identities:6164/6348(97%), Gaps:13/6348(0%), Strand: Plus/Minus

```
Query  21748  GACGGCTCTCAGGCGTGGGAGACGGGAAGTACTCCCCCGTTGTACTCGACCATCTCGCCG  21807

Sbjct  9188   ......................G............G........................  9129


Query  21808  AAGACCGACACGACGTCGAGGGCATGGCCGAGCTCTTCGCCGGCCAGTTCCGAGTAGGCC  21867

Sbjct  9128   ...........................................................G  9069


Query  21868  CGGTGCAGGTTGGCCGGCAGCCGCTCGGGGTCGCGGAGGTCGCCGTACGGGCCGGGCCCC  21927

Sbjct  9068   .............................A.....C........................  9009


Query  21928  CTCTCGCGCGCGAGCTGGAGGGGGGTGAGCCCCGCAGCCCTGCCCTCGGCGGCAAGCTCC  21987

Sbjct  9008   G.......G.....T....................C..........T......G......  8949


Query  21988  GTCAGCCAGTGGAAGTACTCCTCGGTCCGGGTCAGTGCCTCGGGGCCGCTGACGGGTCCG  22047

Sbjct  8948   ...................................C....................G...  8889


Query  22048  TGACCGCCGACGACGGTCAACGGGTCCAGCTTCCGCAGGAGTTCCAGGGTGGCGAGGGAC  22107

Sbjct  8888   ..G................G.............................C..........  8829


Query  22108  CCGGCGACGGAGCCCATCAGCACGAACGGCGTACAGCCGGGAAGCACGACGTCGCCGGCG  22167

Sbjct  8828   ...........C........T.......................................  8769


Query  22168  AACAGCACGCGCTCCTCGGGCAGCCACACCAGCGTGTCGTTGGTGGTGTGCGCGGGGCCC  22227

Sbjct  8768   ............................................................  8709
```

Query 22228 GGATGGATCAGCTCGATCCGGCGCTCCCCCTGGTGGAGGGTGAGCCCCTGGCGGTACGTC 22287

Sbjct 8708   8649

Query 22288 AGGTCGGGCAGCATCAACTCGATGCCGCCCCACTCGACGTCGGGCCACAGCCCGGTCAGA 22347

Sbjct 8648   ................G..................................GT....... 8589

Query 22348 CCGAGTCCGGCGGCTGCCATCTCCGTACGGGTGCGCTCGTGCGCGACCACGACGGTGCCC 22407

Sbjct 8588   ...........T..C................................G............ 8529

Query 22408 GGGCCCGCCAGTACGGCGTTGCCGAAGGTGTGGTCGCCGTGGAAATGGGTGTTGACGATC 22467

Sbjct 8528   8469

Query 22468 AGCCGCGGGGACTCGGGCGTGAGCCCGCCGAGGGCGGTCCGCATGGCCCGCGCCCGCTCC 22527

Sbjct 8468   ....C...................... 8409

Query 22528 TGGGTGGCGGCCGTGTCCACCAGCACGGGCCCACCGCCCCGCCCCGGTTCCAGCAGGATG 22587

Sbjct 8408   .............................T..G..A........................ 8349

Query 22588 CCCGCGTTGCTGACACACCAGCCGCCGTCGGGCTGGATGTACGCGAACACGCCGTCCGCC 22647

Sbjct 8348   ..............G...................................A......... 8289

Query 22648 AGCTCTTCCAGCCTTCCCGGGGCGGCGGTCCGGGCCCCGTTCACGGTGCGGCCACTCACT 22707

Sbjct 8288   .................................T.....G....A.....C...G..... 8229

Query 22708 CGGCAGCCCCCTTCTCGAGGTGGCCCCGGGCCGTCCGAAGCGTGCCCCGGCTGTTGGCGC 22767

Sbjct 8228   ............G...........T...............G................... 8169

Query 22768 CGATGATGTCCCGGGCCTTGGCGAGGGCCTCGGCGATGGTGGTGCCCGGTCCGAAGACGC 22827

Sbjct 8168   .................C..AG........G............................. 8109

Query 22828 TCTCCACGGCGGCGGGATCGAGGGCCACCGTGTGCCGGGCGGTGACCCGGGTGCCCTCAG 22887

Sbjct 8108   .......C.......................................A............ 8049

Query 22888 GACCGCTGCTGAAGAGCCACTCGCCGGAGTGCGCCAGCAGTCCGCGCGGCGGGGTGGTCT 22947

Sbjct 8048   .G..A.................T.................C................... 7989

```
Query  22948  GCTTGTAGACGATTCGCTCGCCCTCGAAGCACAGCCGCACCGACTGGGTGGTGTGCTTGC  23007

Sbjct  7988   .T...........C..........................................G.A.  7929


Query  23008  TGCCGTCCGCGGCGGAGGTCTCCATGTCCATCGTCTGCACCTCGGCGCCCGCGGTGGCGG  23067

Sbjct  7928   .C.....G....................................................  7869


Query  23068  CCTCCGCCGGGTTGGTCTCCAGGTCGAGCCCGGCCACGTGCGGCAGCCGCTTAGGCCACA  23127

Sbjct  7868   G.............C....................................CG.......  7809


Query  23128  GATCGGCGCGGTACAGGAAGTCGTAGACCTCCGCGGCCGGCGCGGCTATGAGCTCCTCGT  23187

Sbjct  7808   ............................................................  7749


Query  23188  CGCTGAAGCTCAGGATCAACTCGTGCGGCTCCTCGGGGCGTTCCGCCCAGGCCCGGAGCG  23247

Sbjct  7748   ........................C.................................T.  7689


Query  23248  ACCCGATCTCGGCGGTGCTGTTGGAGTCCAGCGCGGCGGCGATGCGGTCGGCCGCGCCCG  23307

Sbjct  7688   C..........A................................................  7629


Query  23308  GCTCGCCGTCGGTGGTCCACCGGTGCCCGAGTTCCAGCCGCCCGGGGGTGCCGGGCGAGG  23367

Sbjct  7628   .T.......................T.....................C............  7569


Query  23368  CGTCCTGGAACCGCCAGTGACCCGACATCTCCGTGATCGGTGCCGCCGGGGTCTCCTGGG  23427

Sbjct  7568   ........................................C..G................  7509


Query  23428  CGAAGTCGATCCGGTTGCCTGCCGGGTCGAGCACCCGCCGCGAGGTCCAGCTGCGCACCC  23487

Sbjct  7508   ...................C................T.......................  7449


Query  23488  CGTTGCCGACGACCGCCCAGAGCCGGATGCGCTGGACGCCGCCGGTGCGTTCCAGTTCCT  23547

Sbjct  7448   ....................................T.......................  7389


Query  23548  GGGACCAGATGCAGGGCGCGAAGAGCAGCGGCCAGCGGCCGACGTCCTCGATGAGCGCGT  23607

Sbjct  7388   ............................................................  7329


Query  23608  AGGCGCGGCGGGCCGACGCGGCCAGCTCGGTCACGTGCTCGGCGCTGCGGGTGGGGCTGT  23667

Sbjct  7328   ............................................GAA.............  7269
```

```
Query  23668  CCGCGGTCCTCTCGTGCGTCTCCGTGTCCACGCGGTGGTTCTCCTCAGCCTCGGTGATGT  23727

Sbjct  7268   ...............................................A....A.......  7209


Query  23728  GCGAGATCTCAGTGATGTGTGGGGCGGCTCAGTAGTTTCCGAGGCCTCCGCAGACGTTGA  23787

Sbjct  7208   ...................C.A......................................  7149


Query  23788  TCGCCTGCGCGGTGACGGCCGCCGCCGCGTCCGAGACCAGGTACGCGACCATGGCGGCGA  23847

Sbjct  7148   .....................................G..A.....C.............  7089


Query  23848  CCTCGTCGGGGTCGGTGTAGCGGCCGAGGGGGATCTTGGCGTTGAACCGCTCCAGGACCT  23907

Sbjct  7088   ............................................................  7029


Query  23908  CGTCCTCGGTGGTGTTCCAGATACGGGCGTAGTGCTGGCGCACGTTGCCCGCCATGGGCG  23967

Sbjct  7028   ............................................................  6969


Query  23968  TCTCCACATAGCCGGGGCAGACCGCGTTGACGGTGATTCCGCTCTTGGCCAGTTCGAGGC  24027

Sbjct  6968   ............................................................  6909


Query  24028  CGAGGGCCTTGGTGAACCCGACCACGCCGTGCTTGGAGGCCGAGTAGGGCGCCCCGAACA  24087

Sbjct  6908   ............................................................  6849


Query  24088  CGACGCCCTGCTTGCCGCCCGTGGAGGCGATGCTGACGATCCTGGCGCGCTTCAGCTCAC  24147

Sbjct  6848   ............................................................  6789


Query  24148  GCATCCGGCCGGTGGTCAGCACCTGCTTGGTGACCAGGAAGACGCTGTTGAGGTTGGTGT  24207

Sbjct  6788   ............................................................  6729


Query  24208  TGATGACGTCGAACCAGAGCTCGTCGGGGATCTCGGCGGTGACGCCGCCACCGCTGCGCC  24267

Sbjct  6728   ............................................................  6669


Query  24268  CGGCGTTGTTCACCAGGACCTCCACCGGACCGAACCGGTCGACGGCGGACTGGACGAACC  24327

Sbjct  6668   ......................A..G..................................  6609


Query  24328  GCTGCACGCTCTCGGCTGAGGTCACGTCGCACACGGTTCCGTCGGCCGTCACACCGCTCT  24387

Sbjct  6608   ................C..........................T................  6549
```

```
Query  24388  CCTGGAGCTCCTTGACGGTCTGGGTGAGGGCTTCCTCGTCCCGGGCGCAGAGGTAGACGG  24447

Sbjct  6548   ............................................................  6489


Query  24448  GATGTCCGAGCGCGCCCAGGGAGGCCGCGACGGACCGTCCGATGCCCCGGGTCGCTCCGG  24507

Sbjct  6488   .G..........................................................  6429


Query  24508  TGACGACGGCGGTGCCCCGGGGGGCCTGTACCTGCTCCATGGTGTCGGGCCCGTTCTCTC  24567

Sbjct  6428   ............................................................  6369


Query  24568  AGGCGGCCTGGGCCGCGGCGTGGTTGACCCGGTCGAGGAACTCCCGGGGAGTCTCGATCC  24627

Sbjct  6368   .................................................G.....T....  6309


Query  24628  CGGTGACCTCCTCGTCGGTGAGGTCGACCGCGAACCGCTGCTTCATCAGGGCCGCCGTCT  24687

Sbjct  6308   ............................................................  6249


Query  24688  CCAGCAGCGCCAGCGAGTCGTAGCCGAGGTCGGTGAAGGGCACGTCCAGGTTCTGGCCCG  24747

Sbjct  6248   ..........................................................G.  6189


Query  24748  TGAGCGTGACGTCGTCGTCGGCGCCCGCGCACTCGGTGAGCACGAGGACCAGGTCGTCGA  24807

Sbjct  6188   ............................................................  6129


Query  24808  GGGTGATGGTCATGATGCGAACTCCTTGCGTACGGAATCGGAtgggggtgggggtggggt  24867

Sbjct  6128   ..................................A............-............  6070


Query  24868  g-----cgggt--ga----gatgcgggtggaaatggg-ggtCAGGCGGCCTCGGGCGCAC  24915

Sbjct  6069   .AGGTG.....CC.GTCCG............G..CC.A..............T.....G.  6010


Query  24916  GGACCACCACGGCGGCGTTGAAGCCGCCGTGGCCGCGTGCCAGCACCAGCGCGGTGCGCA  24975

Sbjct  6009   ............................A..A..C..C...........G.....A....  5950


Query  24976  GCGGCGCCTGGCGGGGTGTGCCGCGCACCAGGTCGAGCCGGTGCTCGGGAACGGGGCGGT  25035

Sbjct  5949   ....G.TG....................................................  5890


Query  25036  CGATGCCCGCGGTGGGCGGGATGACTCCGTCGCGCAGGGCGAGCAGGGCCGCCGCCACGT  25095

Sbjct  5889   .........................A..........................G.......  5830
```

```
Query  25096  CGAGCGGGGCTCCACCGCCGAAGAGCCGGCCCGTGAGGGTCTTCGGGGCGGTGACCGGCA  25155

Sbjct  5829   ..........C................................G................  5770


Query  25156  CACCGTAGGGGCCGAAGAGGGCGCGCAGTGCCGCGGCTTCTTCGTCGTCGGCCGTGGGCA  25215

Sbjct  5769   .G...................................C..C...................  5710


Query  25216  GTCCGGCTGCGTCGGCGAAGACCACGTCCACGTCCTCGACGCCCAGCCCGCTGTCGGCGA  25275

Sbjct  5709   ....C..G................................A...................  5650


Query  25276  GGGCGAGCCGTGCGGCGCGTTCGAGACCGGGCGGGCGTCCGGAGCCCGGCCGCGGCGAGA  25335

Sbjct  5649   ............................................................  5590


Query  25336  AGGTGGCCGCGTATCCGGCGATCTCGCCGTACACCCGGGCGCCGCGCGCACGGGCCGCGG  25395

Sbjct  5589   ...........................................C..............C.  5530


Query  25396  TCTCCTCTTCGAGTACGAGCATCGCGCCCCCCTCGCCCGGCACTTGCCCGGCGGCGCGGG  25455

Sbjct  5529   ............................T.................A.............  5470


Query  25456  TGTCGAAGGGCAGGTAGGCGGTGGCGGGGTCGTCGGAGCGGGAGAGCCGGCCGCTCGCGA  25515

Sbjct  5469   ............................................................  5410


Query  25516  GGTGGGAGACCAGCCCCCAGGGGTCGAGGGAGGAGTCCATGCCGCCGGTCAGCGAGAGCT  25575

Sbjct  5409   ..........G.................................................  5350


Query  25576  TGACGCCCTGCCCCAGCGTGCGGCGGGACTGGCCGATGGCGTCGAGGCCGCCCGCCTGCT  25635

Sbjct  5349   ............G...........................A.................T.  5290


Query  25636  CGGAGACGAGGACGCCACTGGGTCCGCGCAGCTTGTGGCGGATGGAGAGCTGGCCGGTGT  25695

Sbjct  5289   .......................................................T....  5230


Query  25696  TGACCGCGTAGAACCAGGCGAAGGACTCGTAGACGCTGACCTGTTGGGGGCCCTGGGTCC  25755

Sbjct  5229   ...........................................C..C.............  5170


Query  25756  ACAGTTTGCGGATCTCCCGGTGGGTGAACTCGAATCCGCCGGTGGCATTCGAGGTGACCA  25815

Sbjct  5169   .....................................A........G.............  5110
```

```
Query  25816  CGCCGCAGCCGTACTCGCCGTATCCGTCCTGTTCCGCCAGTTCGGCGGGGTCGGCGTCGG  25875

Sbjct  5109   ...................................................T........  5050

Query  25876  CGTCGGCCAGCGCCTCGGCGCCCGCGACGAGGGCCAGCCGGGTCACCCGGTCGGTCTGCG  25935

Sbjct  5049   ..........T.................................................  4990

Query  25936  GCAGCAGCCGTCCCGGGATGTGCTCGGACGCCTCGAACCCCGCGATCCGCCCGACCAGCC  25995

Sbjct  4989   ...........................C............GT..................  4930

Query  25996  GGGACGGGTAGCGGGAGGCGTCGTAGTCGACGACCGGCCCGATACCGCTCGTGCCCTGCA  26055

Sbjct  4929   ............................................................  4870

Query  26056  GCGTGGACTGCCACCACTGCTCGGTGCCGAGGCCGTTGGGGGCGGCGACGCCGATGCCGG  26115

Sbjct  4869   .................................................A..........  4810

Query  26116  TGATGACGGCGGTCGGCCGCCCGGTGCGCTCAGGGGTCTCCTGGGCCGCGGGAGTGGCCA  26175

Sbjct  4809   ............................................................  4750

Query  26176  GTGTCATCGCGTGGTTTCCTCTCGGGTGATGACCATGGCGGACTGGAAGCCGCCGAAGCC  26235

Sbjct  4749   ............................................................  4690

Query  26236  GCTGCCCACACTGAGGACGGCGTCCACGGCGTGCTCCCGGGCCTCGTTCGGCACGTAGTC  26295

Sbjct  4689   ............................C......................T........  4630

Query  26296  CAGGTCGCATTCGGGATCGGGCTCGTGAAGGTTGGCCGTGGGGGGAACGGCACCGTTCTC  26355

Sbjct  4629   ............................................................  4570

Query  26356  CATGGCCAGCGCGCAGGCGGCGATCTCGATGGAGCCGATGGCGCCGAGCGAGTGCCCGAC  26415

Sbjct  4569   .............................................C..G...........  4510

Query  26416  CATGGATTTGATGGAGCTGATCGGCACCTCGTAGGCGTGCTGCCCGAGGGTCTCCTTGAA  26475

Sbjct  4509   ......C.....................................................  4450

Query  26476  GGCGGCCGTCTCGTGCCGGTCGTTCTGCTTGGTGCCCGACCCGTGCGCGTTGACGTAGTC  26535

Sbjct  4449   .......................................G....................  4390
```

```
Query  26536  GACGAGGTCGGGGTTGAGCCGCGCCTGGTCCAGCGAGTGCCGGATGGCCTCGGCCATCTC  26595

Sbjct  4389   ............................................................  4330


Query  26596  GTGGCCGTCCGGCCGCAGACCCGTCATGTGGTACGCGTTGCAGCGGGTGGCGTAGCCGCC  26655

Sbjct  4329   ............................................................  4270


Query  26656  GATCACCCCGTAGACATGGGCGCCCCGGGCCCGCGCGTGCTCCAGCTCCTCCAGGACGAA  26715

Sbjct  4269   ...G.........................................T..............  4210


Query  26716  CATCGCGGCGCCCTCGGCGAGTACGAAGCCGTTGCGGGTGCGGTCGAAGGGCCGGGAGGC  26775

Sbjct  4209   ............................................................  4150


Query  26776  GTGCTCGGGGTCGTCGTTGCGGGGCGTGGTGGCCTTGATCGCGTCGAAGCAGGAGACGGC  26835

Sbjct  4149   .......................................G....................  4090


Query  26836  GATCGGCGAGATCGGGGTGTCGGCCGCCCCGGCGATCATGACGTCGACGGTGCCCTCGGC  26895

Sbjct  4089   ......G.....................................................  4030


Query  26896  GATGAGGTCACGCGCGTAGCCCACGGAGTCCAGCCCGGAGGTGCAGCCGGTGGAGACGAC  26955

Sbjct  4029   ....................................C..T....................  3970


Query  26956  CGTGGCGGGGCCTTCGGCGCCCACGGTCCATGCCACCTCGGCCGCCAGGGAACTGGGCAC  27015

Sbjct  3969   .........C....................G.............................  3910


Query  27016  GAAGGCGTCGAAGAGGTGGGGCGAGACGTAGTCGCTGTCCACCTCCCACAGCCGGCCGCT  27075

Sbjct  3909   ............................................................  3850


Query  27076  GTCGCTGAGGACGAGATATTCGCGTTCCAGTCCGGTGGTGCCGCCGACCGCGCTGCCGAT  27135

Sbjct  3849   ...............G...........................................C  3790


Query  27136  GCTCACCCCGGTGCGGTGCGGGTCGAGTTCCACGAATTCGAGGCCGCTGTCCGCCATGCA  27195

Sbjct  3789   .....................A......................................  3730


Query  27196  CTCCCTGGCGCTGACGACGGCGAACTGTGCGGCCCGGTCCAGCCGCCTCGCCTCCCGCGG  27255

Sbjct  3729   ............................................................  3670
```

```
Query  27256  GCTGAGCCCGGACCGCTGCGGATCGAAGTCGACTTCGGCGGCGACCTGTGAGCGGAAGCG  27315

Sbjct  3669   .................................C..........................  3610


Query  27316  GGAGGGGTCGAAGAAGGAGATGGTGCGGGTGGCGGTGCGGCCCGAGACCAGGAGATCCCA  27375

Sbjct  3609   ............................................................  3550


Query  27376  GAACGCCTTCTTGCCCACCCCACCCGGCGCGACGACACCTATCCCGGTGATTGCGACACT  27435

Sbjct  3549   .................................A..G.......................  3490


Query  27436  TCGGTTCACTGTCCTGCCCCTCGTTCGGCTGTCTTCGACTGTCGGCGCAACCCCTTGACC  27495

Sbjct  3489   ............................................................  3430


Query  27496  ACGGCTCGAATTCGACTGGCGGCCCCGTCTCGCCGGGTCCAGCGGTGGTCCACTCCAGGT  27555

Sbjct  3429   ............................................................  3370


Query  27556  CGAGACGGGTCGGACAGGCTCGGCCCGCCCGCCGGAGGTGCCGGGGGCCGAGGCGAGGGA  27615

Sbjct  3369   ............................................................  3310


Query  27616  GGTCCCGTGGAGCGCCGACGAAGTGTGCTGCTGCTGCCCGGACAGGGTGCGCAGCGGGAA  27675

Sbjct  3309   .................T....CA....................................  3250


Query  27676  CGTATGGCCGCCGGACTGTACGGCGCGCGGGAGGAGTTCACCGCGCCCATGGACGAGTTC  27735

Sbjct  3249   ......................AA....................................  3190


Query  27736  TTCGGCCGGCTCGGCACGACCGGCGCACGGCTGCGGTCGGCGTGGCTGCGCCCCGCGCCC  27795

Sbjct  3189   ....................A....................A..................  3130


Query  27796  AACCCGGAGCTGGACGAGTCATCGGTGGCGCAGCCGCTGCTGCTCGCCGTGGGCCACGCG  27855

Sbjct  3129   ....................GG.............A........................  3070


Query  27856  CTGGGACGTGCGGTGGGCACGGCGGCGGAACCTCCCGCGCTGCTGCTCGGGCACAGCGTG  27915

Sbjct  3069   ..................................................A.........  3010


Query  27916  GGCGAGCTGGCGGCGGCCTGCCTGGTGGGCGTCTTCGATCCGGCGGACATCGGCGCGCTG  27975

Sbjct  3009   .........................C..................................  2950
```

```
Query  27976  GCCGCCGCCCGCTCCCGGGCGCTGGAGGGTACGGGGCACGGCGGGATGCTCGCGGTGGCG  28035

Sbjct  2949   ..................T..........C......T.......................  2890


Query  28036  GCGCCGGAGGGGCAACTGGCCGAAGAGCTTGGGGGGTTCGGCGACGGG  28083

Sbjct  2889   ...A.........................C..............A...  2842
```

Range 3: 24465 to 30893

Score:9910 bits(5366), Expect:0.0,

Identities:6095/6451(94%), Gaps:34/6451(0%), Strand: Plus/Minus

```
Query  1      CGTGGCCACCTTCCTCTACCGGCTCGGCCGGTTCTCGTTCAGGCGACGACGGCTCGTCCT  60

Sbjct  30893  ............................................................  30834


Query  61     GATGCTGTGGATCGCGGTCCTCGCCGCCGTCGGCATAGGCGCGGCCAGTGTGTCCTCCGG  120

Sbjct  30833  .....................G........................G...........A.  30774


Query  121    TACGTCGGACACCTTCTCCATCCCCGGCACCCAGTCGCAGAAGGCGCTGGACCTGCTGGA  180

Sbjct  30773  ................G...................C.................C.....  30714


Query  181    GAAGGAGTTCCCGCAGGCGTCGGCCGACGGGGCCACGGCACGCGTGGTGTTCGAGGCGCC  240

Sbjct  30713  ............................................................  30654


Query  241    CGACGGGCAGAAGCTGACGTCAGCGGCGCACAAGGCCGAGGTCGAATCCCTGGTCGACGA  300

Sbjct  30653  ..C..................C.......................G..G.......G...  30594


Query  301    CCTGAAGTCGGCGTCCCAGGTGGCGAGCGTCGCCGACCCCTACACCGGAGGCACCGTCAG  360

Sbjct  30593  ...C......................A.....T...............C...........  30534


Query  361    CAAGGACGGCTCCGTCGCCTACGCCCAGGTGACCTACAAGGTCGTCCCGGACGAGATCAC  420

Sbjct  30533  .....................................G......C..A..C...TG....  30474


Query  421    CGACGCCACCCGCGCCGGCCTGGAGCACGTCGCCGAACAGGGCGAGCGGGCCGGTCTCGC  480

Sbjct  30473  .......G.........A..........................................  30414


Query  481    GGTCAGCATGGGCGGATCGGCCGTCGCCGAGGAGGCCCACCAGAGCACGGCCGAGCTGAT  540

Sbjct  30413  ............T..CA........AA.......T...........G.............  30354
```

```
Query  541    CGGCATCGTGATCGCCGCCCTGGTCATGGTCATCACCTTCGGCTCGCTGGTCGCGGCCGG  600

Sbjct  30353  ............................................................  30294


Query  601    GCTGCCGCTGCTGACCGCGCTCTTCGGCGTGGTCGCGGCGATCTGCGGGATCACCGTGGC  660

Sbjct  30293  A.............................CC............................  30234


Query  661    GAGCTCGGTCATCGGCCTGAGCTCCAGCACGAGCACCCTGGCGCTGATGCTGGGCCTGGC  720

Sbjct  30233  ..C....T......A...C...........C.....G....................C..  30174


Query  721    GGTGGCCATCGACTACGCCCTGTTCATCGTCTCCCGCTACCGCGGCGAGCTGAAGGAGGG  780

Sbjct  30173  ................................................A...........  30114


Query  781    CCACGATCCGGAGGAGGCCGCGGGACGCGCTCTGGGCACCGCCGGATCGGCCGTGGTGTT  840

Sbjct  30113  ........................G.....G...........G........G........  30054


Query  841    CGCCGGGCTGACCGTGGTCATCGCACTGGCCGGACTCAGCGTCATCGGCATCAAGATCCT  900

Sbjct  30053  ............................................................  29994


Query  901    CACCGACATCGGTCTCGGCGCCGCGTTCGCCGTCGTCATCGCCGTGGTCATCGCGCTCAC  960

Sbjct  29993  .T............................G......G......................  29934


Query  961    CCTGCTGCCCGCGATGCTCGGTTTCGCCGGTACGCGGATCTCGGCGGGCAAGCTCAAGAC  1020

Sbjct  29933  ...............................GG............C..G...........  29874


Query  1021   CCGCCGTATGCGGGCGGTCGAGCGCGGCGAGCGCGAGCCGATGGGTGTGCGCTGGTCGCA  1080

Sbjct  29873  ...T............A......AG...........A..T.....C.........G...G  29814


Query  1081   GTTCGTCCTGCGCAACCCGGTGAAGGTCCTCGGAGCCTCGGTGGCCGGCCTGCTCCTGCT  1140

Sbjct  29813  ...........................G.....C.T............T...........  29754


Query  1141   CGCCGTCCCGGCCCTGTCGCTGCAACTCGGCATGGCCGGCGACGAGATGGCCGCCCCCGG  1200

Sbjct  29753  ....A.............................C........TC..C....A.......  29694


Query  1201   CAGCACCCAGCGCATCGCCTACGACACCGTCACCGACGGCTTCGGGGCCGGCTACAACGG  1260

Sbjct  29693  ............................................................  29634
```

```
Query  1261   GCCGCTGACCGTGGTCGTCGACGCCCGCGGCAGCGACGACCCCAAGGCCGCCGCCCAGGA  1320
Sbjct  29633  ............................................................  29574

Query  1321   CGCGGTCACCCTGCTCGAAGACCTGCCCGACGTCGCCTCGGTGAGCCCCGCGTCGTTCAA  1380
Sbjct  29573  ..................C..T.................C..C.......T.........  29514

Query  1381   CGGGACCGGTGACGTGGCCCTGATCCGCGCCGTCCCCGGCTCCTCCCCCACCAGCGAGGA  1440
Sbjct  29513  ..A........................T...........GAG..................  29454

Query  1441   CACGGTCGCCCTGGTGTCCGACATCCGCGACCGGGGCCCCGCCCTGCACGACGACACCGG  1500
Sbjct  29453  ........G...C........................G....G.................  29394

Query  1501   CGCCGAACTGATGGTCACCGGCACCACCGCCCTCAACATCGACATCTCCGGCAAGCTGAA  1560
Sbjct  29393  ......G.....................................................  29334

Query  1561   CGACGCCCTGATCCCGTACCTGTGCGTGGTCGTCGGCCTCGCGCTGATCCTGCTGATGCT  1620
Sbjct  29333  ............................................................  29274

Query  1621   GGTGTTCCGCTCGATCCTCGTACCGCTCAAGGCCGCCGCGGGCTTCCTGCTCAGCGTTCT  1680
Sbjct  29273  .....................C...................................C..  29214

Query  1681   CGCCACGCTCGGCGTCGTCGTCGCCGTCTTCCAGTGGGGCTGGTTCGCGGACGTCTTCGG  1740
Sbjct  29213  ...........................................C................  29154

Query  1741   CGTCGACCAGACCGGCCCGATCGTGAGCGTGCTGCCGATCTTCATGGTCGGCGTGGTGTT  1800
Sbjct  29153  ............................................................  29094

Query  1801   CGGCCTCGCCATGGACTACCAGGTCTTCCTCGTCACCCGGATGCGGGAGGAGTACGTCCA  1860
Sbjct  29093  .........G..................................................  29034

Query  1861   CGGGGCCGAGCCCAAGGAAGCGGTGATCGCCGGGTTCCGGCACGGGGCCCGTGTCGTCAC  1920
Sbjct  29033  ............G...............................................  28974

Query  1921   GGCCGCCGCGGTCATCATGATCTCCGTGTTCGCCGGGTTCCTCTTCAGCGACACCATGCT  1980
Sbjct  28973  .......................................................GC...  28914
```

```
Query  1981   GATCAAGTCGATCGGCCTGGGCCTGGCCGCCGTGTTCCTCGACGCCTTCGTCGTACG  2040

Sbjct  28913  ........................C...............T...................  28854


Query  2041   GATGACCATCGTCCCCGCGGTCATGGCCCTGCTCGGCCGCCGCGCCTGGGCACTGCCCGG  2100

Sbjct  28853  ...............G............................................  28794


Query  2101   CCGACTGGACCGGATACTGCCCAACGTGGACGTCGAGGGCGAGAAGCTGCGGCACCTGCT  2160

Sbjct  28793  ............................................................  28734


Query  2161   GGAGGAGGACACCGAGAGGACCGAGGGCGAGGGGATCCCCGAGCCGGTCCATACCGGCAA  2220

Sbjct  28733  ..............................C..A.................C........  28674


Query  2221   GGGCTGAACCGCCCCCGCAGGCAGGGGCCGGGGCCGAACCGCCTCCCGTCGGCGAGggcc  2280

Sbjct  28673  .......G.......G..C...-.T..G.T.A....-GT..-..-A..--...CC..CT.  28620


Query  2281   gagcccctcgccaccggccacgactcggccg-ccgccaccggccagggcccggccgccg  2339

Sbjct  28619  .-.--.TG............G..G......T.T...-........G..C...AT.....C  28564


Query  2340   gccgTACGATCCCCAGCAGGGCGTAcggccggcaccggcaccc-g--ca-ccggccctgc  2395

Sbjct  28563  .............-...............GC.....-.-....T.CA..C.......CAA  28507


Query  2396   ccacccggccccgcccgccggtcccactccccccggggaccggcgggcggcccccgCACA  2455

Sbjct  28506  ..-..T....................................................--  28450


Query  2456   ACGAGAGGCCGTCAGGACGTGACCGCACCAACCGCT-CCCGGGCGCGGGCCATCGGCTCC  2514

Sbjct  28449  ....................................T............A..........  28390


Query  2515   CTCCGTCGCGCCCGAGACCCTGCTGGACGGGACGAACCGGACCACGGACGTCGCGGTCAT  2574

Sbjct  28389  T...AC.A..................G..A....C........T..............C.  28330


Query  2575   GAAGTGGCTCATC-GGGATCAGGCAGGCGGACCT-GACTCATCCGGGGTTCAGACGCTGG  2632

Sbjct  28329  .C...........A...CG.-.......A.....C...-..C..CTC.G....G......  28272


Query  2633   GTGGTGCCGCTGCTCTGCGCCGGTCTCGGCATGCCGGCCGTCCAGAACTCGGCGAAAGGC  2692

Sbjct  28271  .C.......G.CA..........A.....T..C...AG...G.G.G..G.......C...  28212
```

```
Query  2693   ATCGGAGCCCCTGTACCGACGTTGCTGGCCCTGATCGTCGCGTTCTGTGTCCCACTGCTG  2752

Sbjct  28211  .....TC....G..G.A....C.......G..C.....G..............G......  28152


Query  2753   TGGCGGCAGCAGCGCCCCGTCCTGGTCTTCGCCCTCACCTCCGCCGTCTCCGCGGTGGCC  2812

Sbjct  28151  ...........................................T.......T........  28092


Query  2813   CTCGCCGCGGACGCCGACACGGGCGCGGAGGCCGCGCGGATCGTGGCACTGCTCAACGTG  2872

Sbjct  28091  ......CT..........T.....................................T...  28032


Query  2873   GGCCGCAGCGTCAGGCCCGCCCAGCTGGCGGTCTGTCTGGGGATCGCCATCGCGCAGACG  2932

Sbjct  28031  ..G..G......................................................  27972


Query  2933   ACCGTGGGGGTCGTCGTCCGCGGCGCCGAACAGCCCAACGAGCAGTTCCTGCAGACGCCG  2992

Sbjct  27971  .......C....................GC.....G........................  27912


Query  2993   GTGCTGGCGATCGTGCAGTCGGCGCTCATCGCGGCGGTCGCCGCGGCGGGCCTGGTCGGC  3052

Sbjct  27911  .......................C..........................T.........  27852


Query  3053   CGGGTCATGAACGCCTACATCCGGGCCCTGCACGAACGCGCCGTCCGCCTGGAGGTGGAG  3112

Sbjct  27851  ......G.......G......A....G....................T............  27792


Query  3113   CGCGACCAGCGGgcccgcctcgccgccgccgccgaacgcgcccgcgtcgcccgGGAGATG  3172

Sbjct  27791  .................G..G.......................G...............  27732


Query  3173   CACGACATCCTCGGCCACACCCTCGCCGTGATCGTCGGCCTCGCGGGCGGCGCCGCCGGA  3232

Sbjct  27731  ...........................................................G  27672


Query  3233   CTCACCGAGGCGAAACCGAAGCGGGGCGCCGAGACCCTGCGCATCATCGCCGACAGCGGT  3292

Sbjct  27671  .........A.............A.....G.............................C  27612


Query  3293   CGCGGCGCCCTGGCCGAACTGCGCCGCCTCCTGGCCGTCATCGGCGAGGAACGCGACACC  3352

Sbjct  27611  ..G..........................G..............................  27552


Query  3353   GAGGACGGCCGCCCCCTCGCCCCCCAGCCGGGCCTCGCCGACCTCGACCCCCTCCTGGAA  3412

Sbjct  27551  .........A.......G.....G.................T..............C...  27492
```

```
Query  3413   CGCGTCCGCGGCGCCGGACCCACCGTCACCCTGCACACACGGGGTGCCCTCACCGGTCTC  3472

Sbjct  27491  ...........A..G......G................C.....C..........A....  27432


Query  3473   GCCCCCGGCCTCCAACTGGCCGTCTACCGCGTGGTCCAGGAGTCCCTGACGAACACCCTC  3532

Sbjct  27431  ............................................................  27372


Query  3533   AAGCACGCGGCGTCCGATACGAGGATCCACGTCGCCCTCACCACGGACGACACGTCCGTA  3592

Sbjct  27371  ..A...................C.G...G....A.......G..C..A............  27312


Query  3593   CACGCCACGGTCGAGGACGCCGGTCCCTCCC-GTACCCCACGCTCCTCCGCCCCGCGGGA  3651

Sbjct  27311  .....G.G............-..GA..A...A.C.....G.A....C.........A..G  27253


Query  3652   AGAGGGCCGCGGCCTGGTGGGCATGCGCGAACGGGCGGCGCTGTACGGAGGAAGCGTCAC  3711

Sbjct  27252  C...........................................................  27193


Query  3712   CGCGGGCCCGAACGCCCAGGGCGGCTGGACGGTGGAGGCCCACTTCCAGACCGCCACCAC  3771

Sbjct  27192  .............T....A.................C..........G....A.......  27133


Query  3772   GCCACCCCCGCCACACACCGCACCCACGGAGAAGCGTCCCGCATGACGACTGTCCTCATC  3831

Sbjct  27132  A..G........................................................  27073


Query  3832   GTCGACGACCAGGCCCTGCAACGCCTCGGCTTCAGCATGCTCCTGGAGCAGCACTCCGAC  3891

Sbjct  27072  ......................................................C.....  27013


Query  3892   CTGACGGTGGTCGGCGAGGCCACCCACGGCGCCGAGGCGGTCCGCCTGACGGCCGAACTG  3951

Sbjct  27012  .....C.............................A..............C.........  26953


Query  3952   CGCCCCGACGTCGTCCTGATGGACGTCCGCATGCCCGGCATGGACGGCATCGAGGCCACC  4011

Sbjct  26952  ............................................................  26893


Query  4012   CGCCGCATCGTGGAGTCCGGCGGCCGCTCACGAGTCCTGGTCCTGACCACCTTCGACCTG  4071

Sbjct  26892  .................G..T.......................................  26833


Query  4072   GACGAGTACGCGTACGCGGCCCTGCGCGCCGGAGCCAGCGGCTTCCTCCTCAAGGACGCC  4131

Sbjct  26832  .....A......................................................  26773
```

```
Query  4132   CTCCCCGACGAACTCACGGCGGGCATCCGGGCGGTGGCCTCCGGCGACGCGGTCATCGCC  4191

Sbjct  26772  .................A..A.......................................  26713


Query  4192   CCGGGCCTGACCCGCAAACTGATCGACGCCTTCTCCGCCCACCTCCCGGGCACCACCCCC  4251

Sbjct  26712  ..C............................................C............  26653


Query  4252   GCCCAGGACCGCCAGCTCACCGCCCTCACCACCCGCGAACGCGAGGTGCTGACGGCCATC  4311

Sbjct  26652  .............................A..G...............T...........  26593


Query  4312   GCCACGGGCTGGTCCAACGCCGAGATCGCCACCCGCTTCTCCCTGGCCGAGTCCACGGTC  4371

Sbjct  26592  .....A............A............A............................  26533


Query  4372   AAGTCCCACGTCAGCCACATCCTCGCGAAGATCGGGGCCCGGGACCGCGTCCAGGCGGTG  4431

Sbjct  26532  ...............................................G..T..A......  26473


Query  4432   ATCTTCGCCTACGACATGGGACTGGTGCGGCCGGCCTGAGC-G--CGCCCCGGCCGGTCA  4488

Sbjct  26472  ....................GT.................A.A.AC........-......  26414


Query  4489   GGGACGCGGCAGGCGGGGGTGTTCCGGGCGGCGCAGCTGCTGGAAGGCCCGGTCGTGGTA  4548

Sbjct  26413  ............................T...............................  26354


Query  4549   GACGAGCGGTTCGCCCCGGCCAGGAAGGACATGGCGTACGGCACCCACCAGGATCATGTG  4608

Sbjct  26353  ...............G.A...G..G.................G...........GG....  26294


Query  4609   ATCGCCCGCGGGGTGGCGGGCGTGTACGTCACAGTCCAGGACCGCGAGCGCGCCCTCCAC  4668

Sbjct  26293  .......................AC................................G..  26234


Query  4669   GGCCGGGAGCAGGCGGGGGGTGGTCACGGTGTCCTCGGCACGGAACTTGTCCGCGCCGGT  4728

Sbjct  26233  .........G.............................G....................  26174


Query  4729   GGTGGCGAAACGGGTGGCGAGGGCCCGCTGCTCCTGGCCCAGCAGACTGACCGCGAAACC  4788

Sbjct  26173  ............................................................  26114


Query  4789   CCGGCAGTCCATGAAGGCCTCGTAGGAACTGGAGGTCTCGGCGAGACAGACCAGGACCAG  4848

Sbjct  26113  .............................................G..............  26054
```

```
Query  4849   TGCCGGATCCATGGAGACGGAGCAGAACGAGGTGGCGGTGAATCCGTACGGACGGCCGTC  4908

Sbjct  26053  ............................................................  25994


Query  4909   GGTGTCCTCGGTGGTGACGACGACGACTCCGCCGGCGAGCTGGGCCATGGCGGCCCGGAA  4968

Sbjct  25993  ..C.........................................................  25934


Query  4969   CCTCTCACGGACCTGAGAGGGCTCGACGTCGGCGACGGCCCGCCGGTCGGTGCGGTTCAC  5028

Sbjct  25933  ..C..........C.......T......................................  25874


Query  5029   GGCAGAACCACCACCTGGGAGGCGAAGTTGAGGCCGGCTCCGAAGCCCACGAGGAGCGCG  5088

Sbjct  25873  ...............................................G............  25814


Query  5089   GTGTCCCCGGTGGACGCCTCCTGGGAGGCGAGCAGCGCCTCCAGGGCGAGCGGTATGGAC  5148

Sbjct  25813  ............................................................  25754


Query  5149   GCCGCCGACGTGTTCCCGGAGCGCACCACGTCACGGGAGACGGCGGTGGCGCCGGTGAGT  5208

Sbjct  25753  ............................................................  25694


Query  5209   CCGAGCCGCTCGGTCATCAGCTCGATCATGCGGAGATTGGCCTGGTGGGGAACGAAGGCG  5268

Sbjct  25693  ............................................................  25634


Query  5269   CCGAGCTCGGCCGGCGTCAGCCCGGCCTCGCCGACCGTCCTGGCCAGGGCGGGGGCCACC  5328

Sbjct  25633  .....T.........................G........G...................  25574


Query  5329   TCGTCCATGGCCCAGCGGAAGACCCGGCGGCCGTCCATGCGCATCCACGGCCGTTCGGGG  5388

Sbjct  25573  ...............................................T............  25514


Query  5389   GCGGCCCCGTCGGCCGTGGCCCAGGGGGCGGTCATCCTCAGCGCCTCGCTGTGGGCGCCG  5448

Sbjct  25513  ....G....C.....................................A............  25454


Query  5449   TAGGCCCGCCGTACGACCGGGCCGATCCCCGGTGTGTCGGAGTCGCCGACGACGACCGCG  5508

Sbjct  25453  .....G....TG................................................  25394


Query  5509   CCCGCGCCGTCCGCGAAGAGGAAGGAGATGGTGCGGTCGGTGGGTTCGACGATGTCCGTC  5568

Sbjct  25393  ............................................................  25334
```

```
Query  5569   ATGCGTTCGGCGCCGACGACCAGCACCCGGCGGGCGCTGCCCGCGCGGACCGCGTCGGAG  5628

Sbjct  25333  .......................A....................................  25274


Query  5629   GCCATGGCCAGCGCATGGCAGAAGCCTGCGCAGGCGGCGGACAGATCGACGCCGGCGGCG  5688

Sbjct  25273  ..........................G..........................C......  25214


Query  5689   GCGCCCGCGCCCAGCTCGTGTGCCACGCGCACGGCGAGCGGAGGGGTCTGCACCAGGTTG  5748

Sbjct  25213  A...........................................................  25154


Query  5749   GACATGCTGGCCACCAGCACGAGATCGACTTCGGCGGGCGTGGTTCCGGCCTGCGCCAGG  5808

Sbjct  25153  .....................................................T......  25094


Query  5809   GCCTTCTCCGCCGCCGTGGCGGCCATCATCAGCAGGGTCTCGTCGTCGTCCGCGAAACGG  5868

Sbjct  25093  ..............................................G.............  25034


Query  5869   CGTTCGGCGATGCCGCTGCGGGTCTCGATCCACTCCTCGGTGGATTCGATGAGCCGGCAG  5928

Sbjct  25033  ......A.A...................................A...............  24974


Query  5929   ATCTCGGCGTTGCCGACCACCCGGCGCGGCCGGTAGCCGCCGATCCCGAGAAGCGCGCTG  5988

Sbjct  24973  ......................................................A.....  24914


Query  5989   TGGCGGGTCCCGGCTGTCAGGACGGTCCGTGTCATCGCCCTCTCCTCCGTATCGGCTGCG  6048

Sbjct  24913  ............................................................  24854


Query  6049   GCATCCGTGAACGGATACGTTCTTCGCGCCCAGCGTCGACGGGGACGGTGGAGAAGCCCT  6108

Sbjct  24853  .........................................C..................  24794


Query  6109   TGAGCTCAGCTCAGGGCGCGGGCGGCGGCGGTGAGCCCGGCGGGCGGACGACTTCCGCGC  6168

Sbjct  24793  .......G................A...T..-.-C.G.......................  24736


Query  6169   AACTGCCCGTGCGGTGACCTCTTTTCACCACGACCCACCCATTTGGAACTCAGGAGGAAT  6228

Sbjct  24735  ................G...........................................  24676


Query  6229   ATCCGCCATTCGCAAGAGAGTTCACACTACGATGACGGTGGCCGGATGGCTGATGACCAG  6288

Sbjct  24675  ............................................................  24616
```

```
Query  6289   ATGATCGATCCGCTTTCCGGACCGCGCGGATAGGTGAAGAGCGTCTGGAATCGATGGTGT  6348

Sbjct  24615  .........T....................C.....T................G......  24556


Query  6349   TGACACTCGTTCGACTTCGGGGGGTTCGCGTGCACGCCGAACAGCACACTCTTGTCTTCG  6408

Sbjct  24555  ........................A.......T...........G.........C.....  24496


Query  6409   CGCCGCATGCCGGACCGGATGGCGGCATCGC  6439

Sbjct  24495  ..T..T...........C.GA..........  24465
```

Range 4: 20125 to 24460

Score:7197 bits(3897), Expect:0.0,

Identities:4203/4349(97%), Gaps:27/4349(0%), Strand: Plus/Minus

```
Query  6554   GAAGAAAGCGATTGCCAGCCCTTTACCACCACATCGAGCCGCTGACGAGTGAGTGAAAGC  6613

Sbjct  24460  ................-...........................................  24402


Query  6614   TGAACAATAAATTAGGGGGAACGGCTCGTGATACGGCTGATCATCGCCGAGGATGTACCC  6673

Sbjct  24401  ........G..C..........A.....................................  24342


Query  6674   ATGCTCCGCGGGGCACTGGTGGCGTTGATGGAACTGGAACAGGACCTCAGCGTCGTGGCG  6733

Sbjct  24341  ............................................................  24282


Query  6734   GAGGTGGGCAATGGCAACGACATTCTTCCCACCGCGCTGGAACACCGGCCCGACATCGCC  6793

Sbjct  24281  ............................................................  24222


Query  6794   GTGATCGATATCGATCTGCCCGGCACCGACGGGCTCACGGCCGCCGCGAAGCTCCGTTCC  6853

Sbjct  24221  ............................................................  24162


Query  6854   TGTCTCCCCTCCTGCCGGGTCCTCATCATCACGAGCCTCGGCAATCCGGCCGCGCTCCGC  6913

Sbjct  24161  ..................................................G.........  24102


Query  6914   CGTGCGCTCGCCGCCCAGGTGGACGGATACGTGCTCAAGGACGCGCTGCCGAGCGAACTG  6973

Sbjct  24101  ..C.......................C.....A...........A.....C.........  24042


Query  6974   GCCCAGGCGATACGCAAGGTGGCGGCGGGGCAGCGGGTCATCGATCCGCAACTCGCCCTG  7033

Sbjct  24041  .............................T....................G.........  23982
```

```
Query  7034   TTGGCCTGGGACGGCCCGGCGCAGCAGCTGACCCCACGCGAGGTCGATGTACTGCGCCTG  7093
Sbjct  23981  A...........................................G...............  23922

Query  7094   GCCGCCGCGGGCGAGGACGTGCGCGTCATCGCGAAGGAACTGCACCTGAGCGTCGGCACG  7153
Sbjct  23921  ...................................................A....T...  23862

Query  7154   GTGCGCAACTACCTCACGACGATCGTCCACAAGCTCGGTGCCCGCAATCGGGTCGACGCG  7213
Sbjct  23861  ............................................T...............  23802

Query  7214   GTCCGGATCGCCAGGGACAACGGTGTCATCTGACCACCCCTCATGTCAGCG--TG-C-GT  7269
Sbjct  23801  .....................................T..G.......C..GA..T.A..  23742

Query  7270   G-G--TGA-G--GCGTGGGCTGCCGTCCGGGACGGGGCTGAGGGACGGCGGGCCGGGCCG  7323
Sbjct  23741  .T.CA..GT.GT.............C...........TG....T.......C........  23682

Query  7324   GCAGAACGGCGCGCAGATGGAACCAGCCCCACTCGGTGGTGACGTTCAGCTCTCCGCCGA  7383
Sbjct  23681  ....C...............................................G.......  23622

Query  7384   CGGTAGCCAGCCGGTCGGCGAGATTGCCGAGGCCGCCGCCGCGGCGGCCGGCCTCGGTGC  7443
Sbjct  23621  .....C......................................................  23562

Query  7444   GTGACATCCCGTCCTGGACGCCGTCGTTGGAGATCGCCAGCCACCGCTGCCCGTCGGTCT  7503
Sbjct  23561  ......C........C.........................................C..  23502

Query  7504   CGCCGAACTCGATGCGGCAGTGCCTCGCCGCGCTGTGGCGCAGCAGGTTGGTGACGGCCT  7563
Sbjct  23501  .....................................C......................  23442

Query  7564   CCCGCAGCACTATGGCCAGGATCGAGCCGGTTCCCGGGTCGAGGTCACCCGGCTCGCCCT  7623
Sbjct  23441  .....................C...........................G..........  23382

Query  7624   CGATGGTGGTCTCCACGTCCGCCGCGTCGAGCACTCCGGACACCGAGCGCAGCTCCGCCG  7683
Sbjct  23381  ....C.......................C...............................  23322

Query  7684   TGACGGACAGGTTCCGGCAGCCGCGGGCGACGGCCCGGACGTCGGCCAGTGCCTGCCGTG  7743
Sbjct  23321  .............G..............................................  23262
```

```
Query  7744   CGATGTTCAGCGTCTCGACCAGTTCCTTCCGGGCGCGGTCGTCGTCGACCCCCACCTTCC  7803

Sbjct  23261  .................................................T..........  23202


Query  7804   GCAGCGTCAGTTCACTTTTCAGCGTGATGGCGGAGAGGCTGTAGCCGAGCAGGTCGTGCA  7863

Sbjct  23201  ....G.......................................................  23142


Query  7864   GGTCGCGGGCGACGCGGAGGCGCTCTCCGGAGACCGCGGCCAGGGCCGTCCGGTGGCGCA  7923

Sbjct  23141  .......................................................A....  23082


Query  7924   GCCTGTACTGCTCGACGACGATGTCGCTCAGCCGGGTCAGCCCGTAGACCATCAGCCCGA  7983

Sbjct  23081  ............................................................  23022


Query  7984   TGACCACGGTGGCGAGCGTCAGATAGAGCGACGGGAAGAGGGCGAACGAGCCGAGGGCGA  8043

Sbjct  23021  ...........................C.......GC.............G.........  22962


Query  8044   GTCCGAACACGGCGGCGGCGTTGAGGACGAAGAGGACCCAGGCGAGCGCCGATCCCCGGA  8103

Sbjct  22961  .....C.........................................A....C.......  22902


Query  8104   GCACGACCAGCACGGCCCCCGAGAGGAATCCGCCGAAGCCGCCCCACAGCACGCCGAACA  8163

Sbjct  22901  ............................................................  22842


Query  8164   TGGGAAACGGCGCATAGGCCAGTGCGGCCTGCGGGACGAGTGTCCAGGGGCCGATCCTGG  8223

Sbjct  22841  ........................................C...................  22782


Query  8224   CCCTGAGATGAACGGCATAGGGATTGCAGTGAAATAGCTGCAGCCCGAAGAATGCGATCA  8283

Sbjct  22781  ..................................G.......A.................  22722


Query  8284   GTATCAGCACGGCGCCCACGAGACTCAGGGATTCGTGGTGCCACTTCAGCAGATTGAGTA  8343

Sbjct  22721  ......................G....T...........................C....  22662


Query  8344   TCGCCATCAGTGCGTAGCCCAACTGGACCGCGCCGGTGATGAACAGGGCCAGTCGCAGCC  8403

Sbjct  22661  ..........C......................A..................C.......  22602


Query  8404   CGGTCGCACCTAATTCCACTCCGATTCCCGATTCACTGCCATCGGAGTCGAAGTCGTAGT  8463

Sbjct  22601  ............................G.G.....C...G.........T.........  22542
```

```
Query  8464   GGTTGTCGTCGTGGTGGTCGTAGTCGTGGAGCCGATTCTCTGCGTAGACCACGTAGCTTT  8523
Sbjct  22541  ...------------...............T.............................  22494

Query  8524   CCCCCTGCGCATACATGGACCGACCCCCGCAGTCGTTCACGAGGGCGTGCGAACAACCGT  8583
Sbjct  22493  ............................................................  22434

Query  8584   CACATCACGGAGTGGCGGACCCGCCCTTCGGCGGGCCGTTCCCGCGAGAGGCACGCCACG  8643
Sbjct  22433  ................C.G.........................................  22374

Query  8644   GCGCGCGCCTGCGGACGTCGAATCAGTCTGCGGTGGCAGCGACCGACTGTCGCTCCTGCG  8703
Sbjct  22373  ...........................................T.C..............  22314

Query  8704   CGCCCATGAGCTCCCGCTCGTACACACGCAGGGTGTCGATGAAGGTCTGCCGTTCGGCCG  8763
Sbjct  22313  ...........................................C................  22254

Query  8764   GGCTCAGCGGTTCGAGCGCGACCCGCCAGGCGTTCACACCGTTCTCCAGCCAGGCGCGTA  8823
Sbjct  22253  ................T....................G...............CT...C.  22194

Query  8824   CCGCTTCCCGGTACGGCTCGGCGATGCTGACGATCTTGCGACGCCGGTCGGCCTCGTCCT  8883
Sbjct  22193  ........................................G...................  22134

Query  8884   CCTTGCGGTCCACCACCCCGTACCGGTTCAGCTCCCCGACCAACAGGCTGACCGTGGCCG  8943
Sbjct  22133  ................G.............A.........G.G.........G.......  22074

Query  8944   GGACCAACTCCAGCCGGGTGGCCAGCTCGCTGACGGCGAGCGGCCCGTCGAAGAACAGGT  9003
Sbjct  22073  ..........................................................A.  22014

Query  9004   AGGCCAGCAGGGACAGATGGCGCGGAGCGAGGTTGTACTCCTCCAACTGCTGAGGCACCG  9063
Sbjct  22013  ................G...........................................  21954

Query  9064   GCGTGCGTTTGGTGCGTGCGACGACACGCGGCATGAGGAGCAGGAGCGTCCGGACCCCGT  9123
Sbjct  21953  .............A...........G..................................  21894

Query  9124   TGTCGACGCTCAGCCCTGCCCCCTGCCGACCATCGCTTGACATGACGCAGCCCCCGCAAT  9183
Sbjct  21893  ............................................................  21834
```

```
Query  9184   AGCTTTGCTTTGAAAGCAAAACAGATTTGTCTTGCTTCCCTTGCTGCTCCCGAGCCTACC  9243

Sbjct  21833  ..........................................A.................  21774


Query  9244   GGAAGGGCCGTCCCATGACGGTGGAAGAACAAGCTGATACGGCGCCCCCCGCGCAGTACA  9303

Sbjct  21773  .................................................G..........  21714


Query  9304   GCCCGAAGCGCTGGGCGACGCTGGGCGTCACCCTGTTCGCGGTGTTCATGGACATGGTGG  9363

Sbjct  21713  ........................C...................................  21654


Query  9364   ACAACACCGTCCTCAACGTGGCCCTGCCCGCCGTCCAGCAGGACCTGGACGCCTCCTCCG  9423

Sbjct  21653  ............................................................  21594


Query  9424   CCCAGCTCGAATGGTCGGTGGCGGGATACACGCTGGCCTTCGCCGCCGCCATGATCACCG  9483

Sbjct  21593  ............................................................  21534


Query  9484   GTGCCCGTCTCGGCGACCAGCTCGGGCGCCGGCGGATCTACCTCATCGGCCTCGGCGCCT  9543

Sbjct  21533  ............................................................  21474


Query  9544   TCGTCGTCACCTCGGCACTGGCGGGCGCGGCCGTCAACCCGGAGATGCTCATCGCCTCCC  9603

Sbjct  21473  ...............................G............................  21414


Query  9604   GGATCCTCCAGGGCGCCGCGGCCGCGCTGATGGTGCCGCAGGTCCTGGCGATGCTCCAGG  9663

Sbjct  21413  .C.............G...C.................C......................  21354


Query  9664   TGGACTTCCCGAAGTCCGAGCGCCCCAAGGCGATGTCCATGTACGGCATGTCGCTGGCCG  9723

Sbjct  21353  .........................G..................................  21294


Query  9724   TCGGCGGCATCGGCGGCCCGCTGCTGGGCGGCGTCCTGCTGGAGGCCGACCTCTTCGGCC  9783

Sbjct  21293  .G..........................................................  21234


Query  9784   TGGGCTGGCGGCCGGTCTTCTACGTCAACGTTCCCGTCGGACTGGCCGCGCTGGTCGCCG  9843

Sbjct  21233  ............................................................  21174


Query  9844   CCGCCATCCTGACCCGTGAGTCGCGCGTGGAGACCCGGGAGAGCTTCGACATCCGCGGCA  9903

Sbjct  21173  ..........C.................................................  21114
```

```
Query  9904   CCCTGATCGCGACCGTCGGCCTGATCAGCCTGCTCTTCCCGCTCGTACAGGGACGCGAAC  9963

Sbjct  21113  ............................................................  21054


Query  9964   TGGACTGGCCCTGGTGGACCTTCGCCCTGATGATCGCCTGCCCGGTGATCCTCTGGCTGT  10023

Sbjct  21053  ............................................................  20994


Query  10024  TCGTCCGCTACGAGCACCGGGTGATCGCCCGCGGCGAGTCACCGATCATCGACCCGGCCC  10083

Sbjct  20993  ....................................................T.......  20934


Query  10084  TGCTGCACCACCGCAGCTCGCTCGGCGGCCTGCTCGTCGCCATCCTGTTCTTCTGCGGCA  10143

Sbjct  20933  ..........................................................G.  20874


Query  10144  TGGCCTACCAACTGGTCCTCACGGTCCACCTCCAGACGGGCGAGGGGTACTCCCCGCTGC  10203

Sbjct  20873  .......................................................CA.C.  20814


Query  10204  GCACCGCGGTCGCACTGGTCACGTTCACCGTGGGTGTGGGCATCGGCTCGGCCGTGGCCC  10263

Sbjct  20813  ............................................................  20754


Query  10264  CCCAGCTGATGCCGCTCGGCCGCCGGGTGGTGCTCCTGGGCTGCGCGGTCATGGCCGTCG  10323

Sbjct  20753  .T........................................................G.  20694


Query  10324  GCATGGGTGTCATCACCTGGACCGTCGACCACTACTCCGGCTCGCTGGAGTGGTGGCACC  10383

Sbjct  20693  .......G.........................GG.........................  20634


Query  10384  TGGCACCCGGCATGATCGTCTCCGGTATCGGCCTCGCCATGGTCGCCGGGACGCTGCTCA  10443

Sbjct  20633  ............................................................  20574


Query  10444  CCATCGTGCTGGCGCAGATGCCCAAGTCCGCCTCGGGCGCCGCGTCCTCGCTGATCAACA  10503

Sbjct  20573  .............C..............................................  20514


Query  10504  CAGCCATCCAGATCGGCGTGGCCACCGGCGTCGCGATCGTGGGCACCGTCTACTTCACGC  10563

Sbjct  20513  ............................................................  20454


Query  10564  TGCTGGAGGACCGGCACACGCCCACCGACTCGGCGGTCGTCGGCCTGCTGACCGTCGTCG  10623

Sbjct  20453  ............................................................  20394
```

Query 10624 GCCTCTACACCCTGGCCGGACTGCTCGCCTTCGTGCTGCCGCCGGGACGGGTCGACGTGA 10683

Sbjct 20393 .............................C....A...........C............. 20334

Query 10684 GCGATGTCGACGCGGACACGGACACCGATGCCGACTTCGACCACCACCACGCGACGGCCG 10743

Sbjct 20333 ...........T.C...G................T.C....................... 20274

Query 10744 CCG---GCACGGCCCTGCCCCCGGAGGCCGGGACGAAGGCGCGCGTCGCCCCCTGAGAGG 10800

Sbjct 20273 ...TCG....A.....................G........................... 20214

Query 10801 GCCCCCCGCCCTCGTCAGGAGTGCCCCGCCGCGGTCGGTCGCGGCGGGGCACCGGCATGT 10860

Sbjct 20213 20154

Query 10861 CCGC-GCCCAACGCCGCGGACACCCCCGA 10888

Sbjct 20153 ....C...GG..........TG....G.. 20125

Range 5: 36 to 1634

Score:2429 bits(1315), Expect:0.0,

Identities:1513/1609(94%), Gaps:11/1609(0%), Strand: Plus/Minus

Query 29163 CGA-GACGACGAACGCGCCCAAGCTCCCCTCCCCCGAGCTGTATGTGGAGGTGACCCAGT 29221

Sbjct 1634   ...C.....G......T.....A....................C...........A.... 1575

Query 29222 TCTACGCCCGGCAGATGCACCGGATGGACGGGGACGACTTCGGCGGTTTCGCCGCCACGT 29281

Sbjct 1574   1515

Query 29282 TCGTCGCGGGCGCGGAGTTCCGCCTCGCGGGCGGCACCGTACTGACCGGCCCCGAGGCGA 29341

Sbjct 1514   ....C................ 1455

Query 29342 TCGAggcgggcgcgcgggcggcggcgggcAGGTTCGACGGCGCGCAGCCCCGGCACTGGT 29401

Sbjct 1454   1395

Query 29402 TCGACATGATGACGGTCGAGGAGGCCGACGACGGCACGGTGTCCACCAGCTACTACGCGA 29461

Sbjct 1394   1335

Query 29462 CGGTGACGGTCACTTCCGCAGAGGGTGCCGTCCTGGTGGAGCCGACCTGCTTCGTCCGGG 29521

Sbjct 1334   ................G..GC....................................... 1275

```
Query  29522  ACACCCTGGTCCGGGTGTCCGGTGTGCTGCGCAACCGGTCCCGGGTCATCGAGCGGGACG  29581

Sbjct  1274   .................................G.A........................  1215


Query  29582  ACCTCGTGGTGCGCGCCCGGACGCAGGGCTGAGCCCGTGTGGTCGGTGGTGCTCCCGGTC  29641

Sbjct  1214   ..................................A.........................  1155


Query  29642  AAGTCGTTCAGCAGGGCCAAGAGCCGGCTCGCGGCGGGCCTGGGACCGTGGCGCCAGGAA  29701

Sbjct  1154   .............................A.....A..G.....G...............  1095


Query  29702  CTGGCCCACGCCTTCTTCCTGGACACGCTCTGGGCCGTACGGAACACCGAAGGAGTCCGT  29761

Sbjct  1094   ..........................A..............C...........G......  1035


Query  29762  ACGGTCGTGGTGGTGACGGCCGATCCACTGGCGGCCTCGCAGGCCCGCACCCTGGGCGCG  29821

Sbjct  1034   ..........................C.....C...........................  975


Query  29822  CTGGTCTGCCCGGACGCACCGGATCCCGACCTCAACGACGCCGTACGCCTGGGGGCCGCC  29881

Sbjct  974    ....................C.....................A.................  915


Query  29882  AAGTGCCGCTCGGTGGGCCCCGAGGGTCCGGTGGCGGCGCTCACCGCCGACCTGCCAGGC  29941

Sbjct  914    ..........................C..........TA.................G...  855


Query  29942  CTGCGTCCACGCGAACTGGAGCATGTCCTGCGGGAGGCGAGTCACCATCCGCGGGCCTTC  30001

Sbjct  854    .......................C.....AG.......CC.......C.AA.........  795


Query  30002  GTCGCCGACCACACCGGCGAGGGAACCACCGTCCTGACCGCACTCACCACCGCCGGCCTC  30061

Sbjct  794    ..A...........A.....A..............................T.....A..  735


Query  30062  GCACCCGCCTTCGGCACGGACTCCGCACACCGGCACGCCTCACTCGGGGCGTTCCCCATC  30121

Sbjct  734    T.G.......................C..............G...............G..  675


Query  30122  GACATGCCGAGCGACTGCGGCATCCGCCTGGACGTGGACACCCCGGAGGACCTGGCGCGA  30181

Sbjct  674    ..........................G................................G  615


Query  30182  GTGGCGCTGCGAGGAGTGGGGCCGTACACGGCGGCGCTGTTCCGCCTACGAAAGGCGAGA  30241

Sbjct  614    ..............G...................................G........G  555
```

```
Query  30242  TTCCCGGTGGCGGCGGACCCCGTTGAAGGATTTCAGGGCGTCCAGGACGTTCAGGGTGCT  30301

Sbjct  554    .AT...........C........CC.-..----.----...TG...G.............  504


Query  30302  TCGGCGACGGCTTCCTCGTAACGCTGGATGGCATGCACAAATGTCGCGCGCTCCTGCGGA  30361

Sbjct  503    ................................CC.T..G..G..................  444


Query  30362  CTGAGATCGTCGAAGACCTTGCGCCAGGCCCTGGCCCCGCTGGCCAGCCAGGCGTCGACG  30421

Sbjct  443    .....G................................A.................A.T.  384


Query  30422  GCCGCGCGGGTGGTGGGGTCCTCGGTGAGCGAGACGATGCTGCGCCTGCGGTCGTCGGGA  30481

Sbjct  383    .................A........C.................................  324


Query  30482  TCGGAGCGGCGCTCCACGACCCCTTCGCGCTGCAGGTCGCTGACCATCAGACTGACCGTG  30541

Sbjct  323    .....C...........A..A..C...........A........................  264


Query  30542  GTCGGCGCGACCTCCAGCCGAGCGGCGAGGTCCTTGACGGACGTGGGCCCGTCGAAGATC  30601

Sbjct  263    ...........................................................G  204


Query  30602  AGACAGGACAGCAGGGAGAGATGCCGGGGCGCCAGCCGCAACGAGCGCAACTGCTCGGGC  30661

Sbjct  203    ....................G.......................................  144


Query  30662  ACGGGGGTGCGCTTGGCGCGGGAGACCAACCGCGGCAACGTGACGAGCATCGCCCGAACC  30721

Sbjct  143    .................A..........G...A....GG.....C...........G...  84


Query  30722  GCCTCTTCGGCGCTGAGACGACTACCCGCCTCCGCCTCCGGCTCCGTCT  30770

Sbjct  83     ............G..........G.....................-...  36
```

Range 6: 1682 to 2677

Score:1607 bits(870), Expect:0.0,

Identities:954/996(96%), Gaps:0/996(0%), Strand: Plus/Minus

```
Query  28164  CGGGGTGGCTGTCGCGGGGGTGGCCGACAGGGCGGCCTTCGCCGGGGTGGCCGTCGCGGC  28223

Sbjct  2677   .........C.A..G.A.......T.TTG..A............................  2618


Query  28224  GCTCAACGGACCGCGTCAGACGGTGCTGGCGGGCCCCCGGGAGGCGCTGGCCGCCGTGGA  28283

Sbjct  2617   ....................................G.............A.........  2558
```

```
Query  28284  GCAACACTTGCGGGACCGCGGGATCCTGGTACGCGCGCTGCGGTCGGGGCACGCCTTCCA  28343

Sbjct  2557   .......C..............T...................T.................  2498


Query  28344  CAGCCCCGTCATGGCAGGGGCCGCGCTGCGCTTCGGTGAGGCGCTGGCGGGTTTCGGGCC  28403

Sbjct  2497   ........C.....A....................CC.....A.......CG........  2438


Query  28404  GCGTGCTCCCCGGGCGGCCGCCGGCACGGTGGTGTCGACCCGCACCGGGGTCGCGGTGAC  28463

Sbjct  2437   .T..............A...................C..............A........  2378


Query  28464  CGAGGACCAGGCGCGGGATCCGCACTTCTGGGGCGGGCAGTTGGCCGCCCCGGTGCGGTA  28523

Sbjct  2377   ..C...A.....................................................  2318


Query  28524  CTGGCCGGCCCTGCGGGAACTGCTGGACACCTTGGGGAGGCGGCCGGGGCTGCTGCTGCT  28583

Sbjct  2317   ............................................................  2258


Query  28584  CGACGGCTCCGCGGACCGGAGCCTGAGCGCCCCCGCCCGGCACCACCCCGCGGTGCGGGA  28643

Sbjct  2257   ............................................................  2198


Query  28644  CGGCGCCAGCGAGGTCGTCCCCCTGCTGGCCGTCGGCCGGGACGCCGGTGGCCCGGCCGA  28703

Sbjct  2197   ...............G............................................  2138


Query  28704  CGCGCGGGTGTTCGCCGAGGCGCTGCACCGGCTGGGGGAACAGCGGGATCACACAGGGGA  28763

Sbjct  2137   .........C..................................................  2078


Query  28764  TCACACGGTGGTGAGCTGACCTTGGACGTACTGGATGAGGGCGGCGGGAGTGGTGAGCTC  28823

Sbjct  2077   ......A..................................................T..  2018


Query  28824  GTCGAGCTGTTCGTCTGGCACGCTGACCTTGAAGTCGTCCTGGATGCGGGTCACGATCTC  28883

Sbjct  2017   ...A...........A.............................A..............  1958


Query  28884  GTACACGGTGAGCGAGTCGAAACCGAGGTCGGTGAACTCGGTGTGCAGCGTGGAATCCTT  28943

Sbjct  1957   ............................................................  1898


Query  28944  GATCTCCTCACTGGTGGCTCCGTCGAAGCAGGCGTTCACGACGCTCTGGAACTCGGTGAT  29003

Sbjct  1897   ............................................................  1838
```

```
Query  29004  GGTGAATTCAGCCATGCGGCACACCCTGTGCGGTCGCACTCGACGGCTGCTGGAGACCGG  29063

Sbjct  1837   ............................................................  1778


Query  29064  CTCGACGGCAGCGGGACTCCGTGGGCTCCGCCGCCGCTCGATCCGCCCTCCAGTAGGCGC  29123

Sbjct  1777   ..........CG.....C....C.....................TG..........A...  1718


Query  29124  CCGCAGCATCGCCACCATGACGACTGACGAGACGAC  29159

Sbjct  1717   ..A.................................  1682
```

Figure S1. BLASTN analysis of the alnumycin BGCs of CS 39 and *Streptomyces sp.* CM020. Query refers to the nucleotide sequence of the BGC predicted for CS 39 (NCBI accession number OQ633075) and subject (sbjct) to the alnumycin BGC of *Streptomyces sp.* CM020 (NCBI accession number EU852062). Dots represent identities. Only differences are shown.

```
Query: CS39 Alnumycin BGC (30.779 bp) Query ID: OQ633075 Length: 30779

>AlnR1 [Streptomyces sp. CM020]
Sequence ID: ACI88856.1 Length: 168

Range 1: 12 to 167
Score:264 bits(675), Expect:1e-83,
Method:Compositional matrix adjust.,
Identities:153/156(98%), Positives:155/156(99%), Gaps:0/156(0%)

Query  30765  EPEAEAGSRLSAEEAVRAMLVTLPRLVSRAKRTPVPEQLRSLRLAPRHLSLLSCLIFDGP  30586
Sbjct  12     ..........T.................................................  71

Query  30585  TSVKDLAARLEVAPTTVSLMVSDLQREGvverrsdpddrrrsivSLTEDPTTRAAVDAWL  30406
Sbjct  72     .......................................................I....  131

Query  30405  ASGARAWRKVFDDLSPQERATFVHAIQRYEEAVAEA  30298
Sbjct  132    .......................R............  167

Range 2: 14 to 161
Score:137 bits(346), Expect:2e-39,
Method:Compositional matrix adjust.,
Identities:76/148(51%), Positives:101/148(68%), Gaps:2/148(1%)

Query  9157  DGRQGAGLSVDNGVRTLLLLMPRVVARTKRTPVPQQLEEYNLAPRHLSLLAYLFFDGPLA  8978
Sbjct  14    EAEA.SR.TAEEA..AM.VTL..L.S.A......E..RSLR.........SC.I....TS  73

Query  8977  VSELATRLELVPATVSLLVGELNRYGVVdrkedeadrrrKIVSIAEP--YREAVRAWLEN  8804
Sbjct  74    .KD..A...VA.T....M.SD.Q.E...E.RS.PD....S...LT.DPTT.A.ID...AS  133

Query  8803  GVNAWRVALEPLSPAERQTFIDTLRVYE  8720
Sbjct  134   .AR...KVFDD...Q..A..VRAIQR..  161

>Aln1 [Streptomyces sp. CM020]
Sequence ID: ACI88857.1 Length: 231
Range 1: 1 to 231

Score:381 bits(979), Expect:1e-123,
Method:Compositional matrix adjust.,
Identities:217/234(93%), Positives:225/234(96%), Gaps:3/234(1%)

Query  29618  VWSVVLPVKSFSRAKSRLAAGLGPWRQELAHAFFLDTLWAVRNTEGVRTVVVVTADPLAA  29797
Sbjct  1      M...........................................................  60

Query  29798  SQARTLGALVCPDAPDPDLNDAVRLGAAKCRSVGPEGPVAALTADLPGLRPRELEHVLRE  29977
Sbjct  61     ......................I.................V.................G.  120


Query  29978  ASHHPRAFVADHtgegttvltalttaglapafgtDSAHRHASLGAFPIDMPSDCGIRLDV  30157
Sbjct  121    .R..Q....................S.IS..................V............  180

Query  30158  DTPEDLARVALRGVGPYTAALFRLRKARFPVAADPVEGFQGVQDVQGASATASS  30319
Sbjct  181    ............................Y.......---.A.EG..........  231

>Aln2 [Streptomyces sp. CM020]
Sequence ID: ACI88858.1 Length: 172
Range 1: 27 to 170

Score:187 bits(475), Expect:1e-56,
Method:Compositional matrix adjust.,
Identities:142/144(99%), Positives:144/144(100%), Gaps:0/144(0%)

Query  29173  NAPKLPSPELYVEVTQFYARQMHRMdgddfggfaatfvagaefRLAGGTVLTGPeaieag  29352
Sbjct  27     ............................................................  86

Query  29353  araaagrFDGAQPRHWFDMMTVEEADDGtvstsyyatvtvtsaEGAVLVEPTCFVRDTLV  29532
Sbjct  87     ...........................................Q................  146

Query  29533  RVSGVLRNRSRVIERDDLVVRART  29604
Sbjct  147    .......S................  170
```

```
>AlnJ starter unit acyl carrier protein [Streptomyces sp. CM020]
Sequence ID: ACI88859.1 Length: 84
Range 1: 1 to 83

Score:173 bits(438), Expect:6e-53,
Method:Compositional matrix adjust.,
Identities:83/83(100%), Positives:83/83(100%), Gaps:0/83(0%)

Query  29018  MAEFTITEFQSVVNACFDGATSEEIKDSTLHTEFTDLGFDSLTVYEIVTRIQDDFKVSVP  28839
Sbjct  1      ............................................................  60

Query  28838  DEQLDELTTPAALIQYVQGQLTT  28770
Sbjct  61     .......................  83

>AlnK starter unit acyl transferase [Streptomyces sp. CM020]
Sequence ID: ACI88860.1 Length: 414

Range 1: 233 to 414
Score:272 bits(695), Expect:9e-83,
Method:Compositional matrix adjust.,
Identities:172/182(95%), Positives:173/182(95%), Gaps:0/182(0%)

Query  28234  PRQTVLAGPREALAAVEQHLRDRGILVRALRSGHAFHSPVMAGAALRFGEalagfgprap  28413
Sbjct  233    .............D..........F..............A.E......A....A...C..  292

Query  28414  raaagTVVSTRTGVAVTEDQARDPHFWGGQLAAPVRYWPALRElldtlgrrpgllllldgS  28593
Sbjct  293    ..T..............AE.........................................  352

Query  28594  ADRSLSAPARHHPAVRDGASEVVPLLAVGRDAGGPADARVFAEALHRLGEQRDHTGDHTV  28773
Sbjct  353    ............................................................  412

Query  28774  VS  28779
Sbjct  413    ..  414

Range 2: 6 to 151
Score:267 bits(683), Expect:3e-81,
Method:Compositional matrix adjust.,
Identities:139/146(95%), Positives:143/146(97%), Gaps:0/146(0%)

Query  27637  SVLLLPGQGAQRERMAAGLYGAREEFTAPMDEFFGRLGTTGARLRSAWLRPAPNPELDES  27816
Sbjct  6      T...................E.......................................  65

Query  27817  SVAQPLLLAVGHALGRAVGTAAEPPALLLGHSVGELAAACLVGVFDPADIGALAAARSRA  27996
Sbjct  66     A........................................A.................S  125

Query  27997  LEGTGHGGMLAVAAPEGQLAEELGGF  28074
Sbjct  126    .....Y.......T............  151
>AlnL ketoacyl synthase alpha [Streptomyces sp. CM020]
Sequence ID: ACI88861.1 Length: 421

Range 1: 1 to 421
Score:820 bits(2117), Expect:0.0,
Method:Compositional matrix adjust.,
Identities:418/421(99%), Positives:420/421(99%), Gaps:0/421(0%)

Query  27444  VNRSvaitgigvvapggvGKKAFWDLLVSGRTATRTISFFDPSRFRSQVAAEVDFDPQRS  27265
Sbjct  1      M...........................................................  60

Query  27264  GLSPREARRLDRAAQFAVVSARECMADSGLEFVELDPHRTGVSIGSAVGGTTGLEREYLV  27085
Sbjct  61     ...........................................V................  120

Query  27084  LSDSGRLWEVDSDYVSPHLFDAFVPSSLAAEVAWTVGAEGPATVVSTGCTSGLDSVGYAR  26905
Sbjct  121    ............................................................  180

Query  26904  DLIAEGTVDVMIAGAADTPISPIAVSCFDAIKATTPRNDDPEHASRPFDRTRNGFVLAEG  26725
Sbjct  181    ............................................................  240

Query  26724  AAMFVLEELEHARARGAHVYGVIGGYATRCNAYHMTGLRPDGHEMAEAIRHSLDQARLNP  26545
Sbjct  241    ............................................................  300
```

```
Query  26544  DLVDYVNAHGSGTKQNDRHETAAFKETLGQHAYEVPISSIKSMVGHSLGAIGSIEIAACA  26365
Sbjct  301    ............................................................  360

Query  26364  LAMENGAVPPTANLHEPDPECDLDYVPNEAREHAVDAVLSVGSGFGGFQSAMVITREETT  26185
Sbjct  361    ..............................G.............................  420

Query  26184  R  26182
Sbjct  421    .  421
```

Range 2: 4 to 408
Score:92.0 bits(227), Expect:3e-21,
Method:Compositional matrix adjust.,
Identities:126/414(30%), Positives:186/414(44%), Gaps:25/414(6%)

```
Query  26128  TAVITGIGVAAPNGLGTEQWWQSTLQGTSGIGPVVDYDASRYPSRLVGRIAGFEASEHIP  25949
Sbjct  4      SVA......V..G.V.KKAF.DLLVS.RTATRTISFF.P..FR.QVAAEV------DFD.  57

Query  25948  GR--LLP-QTDRVTRlalvagaealadadadpaelaEQDGYGEYGCGVVTSNATGGFEFT  25778
Sbjct  58     Q.SG.S.REAR.LD.A.QF.VVS.RECMADSGL.FV.L.---PHRT..SVGS.V..TTGL  114

Query  25777  HREIR------KLWTQGPQQVSVYESFAWFYAVNTGQLSIRHKLRGPSGVLVSEQAGGLD  25616
Sbjct  115    E..YLVLSDSGR..EVDSDY..PHLFD.FVPSSLAAEVAWTVGAE..AT.VSTGCTS...  174

Query  25615  AIGQSRRTLGQG-VKLSLTGGMDSSLDPWGLVSHLA-SGRLSRSDDPATAYLPFDTRAAG  25442
Sbjct  175    SV.YA.DLIAE.T.DVMIA.AA.TPIS.IAVSCFD.IKATTP.N...EH.SR...RTRN.  234

Query  25441  QVPGEGGAMLVLeeetaarargaRVYGEIAGYAA---TFsprpgsgrppGLERAARLALA  25271
Sbjct  235    F.LA..A..F....LEH......H...V.G...TRCNAYHMTGLRPDGHEMAE.I.HS.D  294

Query  25270  DSGLGVEdvdvvfadaAGLPTaddeeaaalraLFG--PYGVPVTAPKTLTGRLFgggapl  25097
Sbjct  295    QAR.NPDL..Y.N.HGS.TKQN.RH.T..FKETL.QHA.E..ISSI.SMV.HSL.AIGSI  354

Query  25096  dvaaallalrdgVIPPTAGIDRPVPEHRLDLVRGTPRQAPLRTALVLARGHGGF  24935
Sbjct  355    EI..CA..MEN.AV....NLHE.D..CD..Y.PNEA.EHGVDAV.SVGS.F...  408
```

>AlnM ketoacyl synthase beta [Streptomyces sp. CM020]
Sequence ID: ACI88862.1 Length: 428

Range 1: 1 to 417
Score:620 bits(1598), Expect:0.0,
Method: Compositional matrix adjust.,
Identities:411/417(99%), Positives:413/417(99%), Gaps:0/417(0%)

```
Query  26182  MTLATPAAQETPERTGRPTAVITGIGVAAPNGLGTEQWWQSTLQGTSGIGPVVDYDASRY  26003
Sbjct  1      ............................................................  60

Query  26002  PSRLVGRIAGFEASEHIPGRLLPQTDRVTRlalvagaealadadadpaelaEQDGYGEYG  25823
Sbjct  61     .......D....A............................N.............  120
Query  25822  CGVVTSNATGGFEFTHREIRKLWTQGPQQVSVYESFAWFYAVNTGQLSIRHKLRGPSGVL  25643
Sbjct  121    ............................................................  180

Query  25642  VSEQAGGLDAIGQSRRTLGQGVKLSLTGGMDSSLDPWGLVSHLASGRLSRSDDPATAYLP  25463
Sbjct  181    ..................R.........................................  240

Query  25462  FDTRAAGQVPGEGGAMLVLeeetaarargaRVYGEIAGYAATFsprpgsgrppGLERAAR  25283
Sbjct  241    ............................................................  300

Query  25282  LALADSGLGVEdvdvvfadaAGLPTaddeeaaalraLFGPYGVPVTAPKTLTGRLFggga  25103
Sbjct  301    ............................................................  360

Query  25102  pldvaaallalrdgVIPPTAGIDRPVPEHRLDLVRGTPRQAPLRTALVLARGHGGFN  24932
Sbjct  361    ....................................HT................  417
```

Range 2: 38 to 426
Score:146 bits(368), Expect:2e-39,
Method:Compositional matrix adjust.,
Identities:123/402(31%), Positives:186/402(46%), Gaps:19/402(4%)

```
Query  27378  FWDLLVSGRTATRTISFFDPSRFRSQVAAEVDFDPQRSGLSPREARRLDRAAQFAVVSAR  27199
Sbjct  38     W.QSTLQ.TSGIGPVVDY.A..YP.RLVGRI.GFEAAEHIPG.LLPQT..VTRL.L.AGA  97
```

```
Query  27198  ECMADSGLEFVEL------DPHRTGVSIGSAVGGTTGLEREYLVLSDSGRLWEVDSDYVS  27037
Sbjct  98     .AL..ADANPA..AEQDGYGEYGC..VTSN.T..FEFTH..IR------K..TQGPQQ..  151

Query  27036  PHLFDAFVPSSLAAEVAWTVGAEGPATVVSTGCTSGLDSVGYARDLIAEGTVDVMIAGAA  26857
Sbjct  152    VYESF.WFYAVNTGQLSIRHKLR..SG.LVSEQAG...AI.QS.RTLRQ.-.KLSLT.GM  210

Query  26856  DTPISPIAVSCFDAIKATTPRNDDPEHASRPFDRTRNGFVLAEGAAMFVLEELEHARARG  26677
Sbjct  211    .SSLD.WGLVSHL.-SGRLS.S...AT.YL...TRAA.Q.PG..G..L....ETA.....  269

Query  26676  AHVYGVIGGYATRCNAYHMTGLRPDGHEMAEAIRHSLDQARLNPDLVDYVNAHGSGTKQN  26497
Sbjct  270    .R...E.A...ATFSPRPGS.-..P.L.--R.A.LA.ADSG.GVED..V.F.DAA.LPTA  326

Query  26496  DRHETAAFKETLGQHAYEVPISSIKSMVGHSLGAIGSIEIAACALAMENGAVPPTANLHE  26317
Sbjct  327    .DE.A..LRALF.P--.G..VTAP.TLT.RLF.GGAPLDV..AL..LRD.VI....GIDR  384

Query  26316  PDPECDLDYVPNEAREHAVDAVLSVGSGFGGFQSAMVITREE  26191
Sbjct  385    .V..HR..L.RGTP.HTPLRTA.VLAR.H...NA.V.VRAP.  426

>AlnN acyl carrier protein [Streptomyces sp. CM020]
Sequence ID: ACI88863.1 Length: 84

Range 1: 1 to 84
Score:147 bits(370), Expect:1e-43,
Method:Compositional matrix adjust.,
Identities:84/84(100%), Positives:84/84(100%), Gaps:0/84(0%)

Query  24820  MTItlddlvlvlTECAGADDDVTLTGQNLDVPFTDLGYDSLALLETAALMKQRFAVDLTD  24641
Sbjct  1      ............................................................  60

Query  24640  EEVTGIETPREFLDRVNHAAAQAA  24569
Sbjct  61     ........................  84

>AlnP ketoreductase [Streptomyces sp. CM020]
Sequence ID: ACI88864.1 Length: 263

Range 1: 1 to 263
Score:484 bits(1247), Expect:5e-159,
Method:Compositional matrix adjust.,
Identities:263/263(100%), Positives:263/263(100%), Gaps:0/263(0%)

Query  24547  MEQVQAPRGTAVVTGATRGIGRSVAASLGALGHPVYLCARDEEALTQTVKELQESGVTAD  24368
Sbjct  1      ............................................................  60

Query  24367  GTVCDVTSAESVQRFVQSAVDRFGPVEVLVNNAGRSGGGVTAEIPDELWFDVINTNLNSV  24188
Sbjct  61     ............................................................  120

Query  24187  FLVTKQVLTTGRMRELKRARIVSIASTGGKQGVVFGAPYSASKHGVVGFTKALGLELAKS  24008
Sbjct  121    ............................................................  180
Query  24007  GITVNAVCPGYVETPMAGNVRQHYARIWNTTEDEVLERFNAKIPLGRYTDPDEVaamvay  23828
Sbjct  181    ............................................................  240

Query  23827  lvsdaaaavtaqainVCGGLGNY  23759
Sbjct  241    .......................  263

>AlnQ aromatase [Streptomyces sp. CM020]
Sequence ID: ACI88865.1 Length: 331

Range 1: 1 to 327
Score:629 bits(1621), Expect:0.0,
Method:Compositional matrix adjust.,
Identities:311/327(95%), Positives:320/327(97%), Gaps:0/327(0%)

Query  23698  VDTETHERTADSPTRSAEHVTELAASARRAYALIEDVGRWPLLFAPCIWSQELERTGGVQ  23519
Sbjct  1      M.........................................................I.  60

Query  23518  RIRLWAVVGNGVRSWTSRRVLDPAGNRIDFAQETPAAPITEMSGHWRFQDASPGTPGRLE  23339
Sbjct  61     ......................................................S.....  120

Query  23338  LGHRWTTDGEPGAADRIAAALDSNSTAEIGSLRAWAERPEEPHELILSFSDEELIAAPAA  23159
Sbjct  121    ..........................V...A...........D.................  180
```

```
Query  23158  EVYDFLYRADLWPKRLPHVAGLDLETNPAEAATAGAEVQTMDMETSAADGSKHTTQSVRL  22979
Sbjct  181    ...........E.............S....P....................T........  240

Query  22978  CFEGERIVYKQTTPPRGLLAHSGEWLFSSGPEGTRVTARHTVALDPAAVESVFGPGTTIA  22799
Sbjct  241    ..................................L.........................  300

Query  22798  EALAKARDIIGANSRGTLRTARGHLEK  22718
Sbjct  301    Q..LR.................S...Q  327

>AlnR cyclase [Streptomyces sp. CM020]
Sequence ID: ACI88866.1 Length: 311

Range 1: 1 to 311
Score:598 bits(1542), Expect:0.0,
Method:Compositional matrix adjust.,
Identities:302/311(97%), Positives:305/311(98%), Gaps:0/311(0%)

Query  22691  VNGARTAAPGRLEELADGVFAYIQPDGGWCVSNAGILLEPGRGGGPVLVDTAATQERARA  22512
Sbjct  1      MT.D........................................................  60

Query  22511  MRTALGGLTPESPRLIVNTHFHGDHTFGNAVLAGPGTVVVAHERTRTEMAAAGLGLTGLW  22332
Sbjct  61     ..A......................................................T..  120

Query  22331  PDVEWGGIELMLPDLTYRQGLTLHQGERRIELIHPGPAHTTNDTLVWLPEERVLFAGDVV  22152
Sbjct  121    ............................................................  180

Query  22151  LPGCTPFVLMGSVAGSLATLELLRKLDPLTVVGGHGPVSGPEALTRTEEYFHWLTELAAE  21972
Sbjct  181    ..................A........................................K  240

Query  21971  GRAAGLTPLQLARERGPGPYGDLRDPERLPANLHRAYSELAGEELGHALDVVSVFGEMVE  21792
Sbjct  241    ..............T.............................................  300

Query  21791  YNGGVLPVSHA  21759
Sbjct  301    .......L...  311


>AlnT hydroxylase [Streptomyces sp. CM020]
Sequence ID: ACI88867.1 Length: 413

Range 1: 5 to 399
Score:635 bits(1638), Expect:0.0,
Method:Compositional matrix adjust.,
Identities:386/395(98%), Positives:389/395(98%), Gaps:0/395(0%)

Query  20348  TPLTAWppgarrpcprllprttrSMGGARMLDTEELaqqaaesargaeearKLDPDVVKL  20527
Sbjct  5      ............................................................  64

Query  20528  LVEAGFARHFVPRECGGTEGTFAELTEAVAQVGAACPATAWCASLAANLGRMAAYLPAEG  20707
Sbjct  65     ..............................R.............................  124

Query  20708  YREVWAQGPDALVVGSLSPFGKAVPATGGWTLSGRWPYISAVAYADWMLLCGTLPDGAGP  20887
Sbjct  125    .......................A..A....V...............L...A..S.....  184

Query  20888  RVFAVPRDevevveswesvGMRATGSHTVVASDVFVPERRAFARADLLTGRPTASTAACH  21067
Sbjct  185    ............................................................  244

Query  21068  TVPLEAANGLSFagpllgaaegalaQWSAYAVVKARSVLLRPQAPGPSREALAGVLAHSA  21247
Sbjct  245    ..L.........................................................  304

Query  21248  GETDAARLLLERCAQVADLGAEVTPEQTRRNLRDTALSVRMLASAVNRLAANGGTTGYGE  21427
Sbjct  305    ........................L...................................  364

Query  21428  SAPLQRYWRDVNTSATHVALQFESAALAYAEGRLW  21532
Sbjct  365    ...................................  399

>AlnR2 TetR family regulator [Streptomyces sp. CM020]
Sequence ID: ACI88868.1 Length: 198

Range 1: 1 to 195
Score:267 bits(682), Expect:4e-84,
```

```
Method:Compositional matrix adjust.,
Identities:191/195(98%), Positives:192/195(98%), Gaps:0/195(0%)

Query  19672  MLTAMAAAPSERADAVRNRQKILaaaarlvaagaaERLSLDEVARVADVGVGTVYRRFGD  19851
Sbjct  1      ............................................................  60

Query  19852  RAGLVFALLEEQHQLFRARVVEGPPPLGPRADAGDRLRAFLHALVDLSVGQrellllaes  20031
Sbjct  61     ...........................G................................  120

Query  20032  ssppar YLSASYDFQHAHAGRLIAELRpdadadfladallapfapSLIDHQSRVRGFSPE  20211
Sbjct  121    ...................................................C........  180

Query  20212  RIKAGFDQLLRSTLE  20256
Sbjct  181    .........V...P.  195

>Aln3 [Streptomyces sp. CM020]
Sequence ID: ACI88869.1 Length: 155

Range 1: 1 to 155
Score:273 bits(698), Expect:6e-87,
Method:Compositional matrix adjust.,
Identities:154/155(99%), Positives:155/155(100%), Gaps:0/155(0%)

Query  19548  VAADQFDPAPTTPAEGDPGSYNLPVMQEFRQNGGKVGGPFEGGSLILLTTKGAKSGVVRT  19369
Sbjct  1      M...........................................................  60

Query  19368  VPTLFFAQSDGSLLIFGSNGGADTHPAWFHNIRVNPRVTVETGEETYSAVATEVPAGSEE  19189
Sbjct  61     ............................................................  120

Query  19188  RDRLfaeaaaevqafaayqaQTERKIPLVILSRKD  19084
Sbjct  121    ...................................  155

>Aln4 ketoreductase [Streptomyces sp. CM020]
Sequence ID: ACI88870.1 Length: 354

Range 1: 1 to 354
Score:669 bits(1726), Expect:0.0,
Method:Compositional matrix adjust.,
Identities:345/354(97%), Positives:349/354(98%), Gaps:0/354(0%)

Query  19077  VRYTLLGRTGVRISRLALGTMTFGDDWKLPEETRTRIFDQYAEAGGNFIDTADEYGDGSA  18898
Sbjct  1      M.............................A..A..........................  60

Query  18897  ETTLGKLLAGRRDEFVLSTKYTMQTRPGDLNSAGNHRKNLVRSLEASLRRLGTDHIDMLW  18718
Sbjct  61     .......................R.............................V....  120

Query  18717  VHARDTLTPVPEVMRALDDQVRAGKVLYVGVSDWPAWEVAQANTLAELRDWSPFAGLQIR  18538
Sbjct  121    ............................................................  180

Query  18537  YNLLERTVERELLPMAHAFDLPVFAWGPLADGRLTGKYLRSEEGRLDHVAWGQGGTGPGD  18358
Sbjct  181    ............................................................  240

Query  18357  DvveetvrvaeaAGISPAQVALAWLLSRPANVVPILGATKPEQLADNLGAVEAVLEDDWL  18178
Sbjct  241    ..............V.......................................Q....  300

Query  18177  AGLERVSSVAPGFPHDFLRFPAMRDAIYGDRWQQVDDLRTTARRVPADDRYGAA  18016
Sbjct  301    .......R.E............................................  354

>AlnO [Streptomyces sp. CM020]
Sequence ID: ACI88871.1 Length: 152

Range 1: 1 to 152
Score:309 bits(791), Expect:2e-99,
Method:Compositional matrix adjust.,
Identities:150/152(99%), Positives:152/152(100%), Gaps:0/152(0%)

Query  17442  MPNYVDAPEGWWREFIMADPPRTAKASVVRKDGSPHVVPVGVIMDGDDIIYTCQKDSVKG  17621
Sbjct  1      ............................................................  60
```

```
Query  17622  RSLQRDGRIAMLWDDERPPFSFVLVRGRATLSEDIDELRAWTARIGGRYHGKRREEEFSE  17801
Sbjct  61     ..................................L.........................  120

Query  17802  RFTIPNGVVVRVKVEQIVAKVNLSETVNVKDS  17897
Sbjct  121    ...............................A  152
```

>Aln5 [Streptomyces sp. CM020]
Sequence ID: ACI88872.1 Length: 176

Range 1: 1 to 176
Score:315 bits(807), Expect:3e-101,
Method:Compositional matrix adjust.,
Identities:164/191(86%), Positives:168/191(87%), Gaps:15/191(7%)

```
Query  16867  VTPEEVVGVWRLASYTEVGEDGGTVAGPLGEAPTGLLIYTADGHVAVSMMKTGDAPALET  17046
Sbjct  1      M................................A..........................  60

Query  17047  YMGYSGQWRLAGDRMTHRVQVSAHPRMAGTEQIRRVALDGETLSLRGTAVTPVGGRAPER  17226
Sbjct  61     ............................................................  120

Query  17227  VLTWRRANPDGIAADGIATDRIAADGTASGSTAIDSTAFDNTAFDNTAFDSTAFDSTASD  17406
Sbjct  121    .......K.E.----------............F.G.GSGG....S.-----........  165

Query  17407  ETAKQQEKETH  17439
Sbjct  166    ...E.......  176
```

>Aln6 [Streptomyces sp. CM020]
Sequence ID: ACI88873.1 Length: 359

Range 1: 1 to 354
Score:660 bits(1704), Expect:0.0,
Method:Compositional matrix adjust.,
Identities:350/354(99%), Positives:352/354(99%), Gaps:0/354(0%)

```
Query  15791  MPRRTFVSRHGlllaaaalGTVMTTPGTSTAKPGSRGYRMVWDDFAEGFRTEGEGARWFH  15970
Sbjct  1      ..............................................D.............  60

Query  15971  VAGGPYRADDGIVTTRPGELSVRARGSHPATGEPAFTQTIPAENPVGMPGSGDHAKFIAY  16150
Sbjct  61     .............A..R...........................................  120

Query  16151  TSHTASSGHPGFDAHEGYELLFETRLSGRTYGTADHPFGDAVRDPEDDLRLASAMMLTTD  16330
Sbjct  121    ............................................................  180

Query  16331  PETSVSFDFVVTNKRIYAWYGRPTFLRGQLGDYASFAHTVPLVARRPGDSHDFGIAYDRA  16510
Sbjct  181    .............................................K..............  240

Query  16511  AGVVRWLIDGEEHFRVDRIGHRLDRSTATLDEGGEETLVRPRQlnaglglltllDASWPT  16690
Sbjct  241    ............................................................  300

Query  16691  DKGLVRLSARKHTTYYRPSVGAPQEQTFADEDSTDAGRLFGQGAAVRLGSYRVT  16852
Sbjct  301    ......................................................  354
```

Range 2: 21 to 356
Score:179 bits(454), Expect:2e-51,
Method:Compositional matrix adjust.,
Identities:122/339(36%), Positives:172/339(50%), Gaps:12/339(3%)

```
Query  13847  TAQSAPTASRAGAGG-GTVLFQDTYGNGF-TTGENGNWLLQGDAEWPTGDAVVTTPGGVL  13674
Sbjct  21     .VMTT.GT.T.KP.SR.YRMVW.DFAD..R.E..GAR.FHVAGGPYRAD.GI.A.RR.E.  80

Query  13673  NVVPTGVNPKTGDPAYARST---GPDGGADSDDHIKWIAFPNRFTAENVPGFEVPDTGSI  13503
Sbjct  81     S.RAR.SH.A..E..FTQTIPAEN.V.MPG.G..A.F..YTSHTASSGH...DAHEGYEL  140

Query  13502  SCVHKVAGRTFGT-ENPFGSTVKDPASDIRLASIALITADFESRAIADFSVTNDTIYAIY  13326
Sbjct  141    LFETRLS...Y..ADH...DA.R..ED.L....AMML.T.P.TSVSF..V...KR...W.  200

Query  13325  ER---LPSDTEDYASYGYAIPLAKTAPGAMHELEVRLDQSGKRVTWFVDGRQKLQTDKIG  13155
Sbjct  201    G.PTF.RGQLG....FAHTV..VARK..DS.DFGIAY.RAAGV.R.LI..EEHFRV.R..  260
```

```
Query  13154  TRAFDRKYMTLDHGGTEERVELDQITCGMGLGSlldgakpgaadggaLVRLKHKEGFYFD  12975
Sbjct  261    H.-L..STA...E..E.TL.RPR.LNA.L..LT...ASW.TDKGLVR.SAR..TT--.YR  317

Query  12974  PRRGEPVEQKFFDPESKVENRLFGQGSEIRADWTKVIRR  12858
Sbjct  318    .SV.A.Q..T.A.ED.TDAG......AAV.LGSYR.TS.  356

>AlnB [Streptomyces sp. CM020]
Sequence ID: ACI88874.1 Length: 227

Range 1: 1 to 225
Score:389 bits(1000), Expect:2e-126,
Method:Compositional matrix adjust.,
Identities:220/225(98%), Positives:224/225(99%), Gaps:0/225(0%)

Query  15018  VTGAPAAADRGVILDLDGtladtpaaiatitaEVLAAMGTAVSRGAILSTVGRPLPASLA  15197
Sbjct  1      MS..........................................................  60

Query  15198  GLLGVPVEDPRVAEATEEYGRRFGAHVRAAGPRLLYPGVLEGLDRLSAAGFRLAMATSKV  15377
Sbjct  61     ............................................................  120

Query  15378  EKAARAIAELTGLDTRLTVIAGDDSVERGKPHPDMALHVAKGLGLAPERCVVIGDGVPDA  15557
Sbjct  121    ........................................R...IP..............  180

Query  15558  EMGRAAGMTVIGVSYGVSGPDELMRAGADTVVDSFPAAVTAVLDG  15692
Sbjct  181    .............................................  225

>AlnA [Streptomyces sp. CM020]
Sequence ID: ACI88875.1 Length: 306

Range 1: 1 to 305
Score:510 bits(1313), Expect:3e-167,
Method:Compositional matrix adjust.,
Identities:305/305(100%), Positives:305/305(100%), Gaps:0/305(0%)

Query  14101  MERQPDQLLEVSDEIATALAERRPVVALESSLITTDPSSETASLIEKAVRGAGAVPATIG  14280
Sbjct  1      ............................................................  60

Query  14281  IAGGKLVVGLTDSLIERFASTKGIPKISARDiggalaggglgattvagtivIAERAGIQV  14460
Sbjct  61     ............................................................  120

Query  14461  FTTAGIGGVHRRGEDTLDISPDLLQFRKTKMTVVSGGAKSILDHRLTAEYLETAGVPVYG  14640
Sbjct  121    ............................................................  180

Query  14641  YRTDKLAAFVVREADVPVTRMDDLHTAARAAEAHWQVNGPGTVLLTSPIDeqdavdeaiv  14820
Sbjct  181    ............................................................  240


Query  14821  eaaiaealaqCDQEGIVGNAVSPYLMKALARASGGMLPKAGRSLLLSTARVAGEFSAALS  15000
Sbjct  241    ............................................................  300

Query  15001  AVQAE  15015
Sbjct  301    .....  305

>Aln7 [Streptomyces sp. CM020]
Sequence ID: ACI88876.1 Length: 352

Range 1: 1 to 352
Score:647 bits(1669), Expect:0.0,
Method:Compositional matrix adjust.,
Identities:347/352(99%), Positives:350/352(99%), Gaps:0/352(0%)

Query  13910  MRHKvayavvaaaavvALLPATAQSAPTASRAGAGGGTVLFQDTYGNGFTTGENGNWLLQ  13731
Sbjct  1      ..............................Q..S............T.............  60

Query  13730  GDAEWPTGDAVVTTPGGVLNVVPTGVNPKTGDPAYARSTGPDGGADSDDHIKWIAFPNRF  13551
Sbjct  61     ............................................................  120

Query  13550  TAENVPGFEVPDTGSISCVHKVAGRTFGTENPFGSTVKDPASDIRLASIALITADFESRA  13371
Sbjct  121    ............................................................  180
```

```
Query  13370  IADFSVTNDTIYAIYERLPSDTEDYASYGYAIPLAKTAPGAMHELEVRLDQSGKRVTWFV  13191
Sbjct  181    ............................................................  240

Query  13190  DGRQKLQTDKIGTRAFDRKYMTLDHGGTEERVELDQITCGMGLGSlldgakpgaadggaL  13011
Sbjct  241    ............................................................  300

Query  13010  VRLKHKEGFYFDPRRGEPVEQKFFDPESKVENRLFGQGSEIRADWTKVIRRP  12855
Sbjct  301    ......D............A................................  352

Range 2: 22 to 342
Score:172 bits(436), Expect:4e-49,
Method:Compositional matrix adjust.,
Identities:124/331(37%), Positives:172/331(51%), Gaps:14/331(4%)

Query  15851  TVMTTPGTSTAKPGSRGYRMVWDDFAEGFRTEGEGARWFHVAGGPYRADDGIVTTRPGEL  16030
Sbjct  22     .AQSA.TA.Q.GS.G-.TVLFQ.TYGT..-.T..NGN.LLQGDAEWPTG.AV...PG.V.  79

Query  16031  SVRARGSHPATGEPAFTQTIPAENPVGMPGSGDHAKFIAYTSHTASSGHPGFDAHEGYEL  16210
Sbjct  80     N.VPT.VN.K..D..YARST---G.D.GAD.D..I.W..FPNRFTAENV...EVPDTGSI  136

Query  16211  LFETRLSGRTYGTADHPFGDAVRDPEDDLRLASAMMLTTDPETSVSFDFVVTNKRIYAWY  16390
Sbjct  137    SCVHKVA...F..-EN...ST.K..AS.I....IALI.A.F.SRAIA..S...DT...I.  195

Query  16391  GRPTFLRGQLGDYASFAHTVPLVARRPGDSHDFGIAYDRAAGVVRWLIDGEEHFRVDRIG  16570
Sbjct  196    E.---.PSDTE....YGYAI..AKTA..AM.ELEVRL.QSGKR.T.FV..RQKLQT.K..  252

Query  16571  HR-LDRSTATLDEGGEETLVRPRQlnaglglltllDASWP--TDKG-LVRLSARKHTTYY  16738
Sbjct  253    T.AF..KYM...H..T.ER.ELD.ITC.M..GS...GAK.GAA.G.A....K-H.DGF.F  311

Query  16739  RPSVGAPQEQTFADEDSTDAGRLFGQGAAVR  16831
Sbjct  312    D.RR.E.VA.K.F.PE.KVEN......SEI.  342

>AlnR3 SARP family regulator [Streptomyces sp. CM020]
Sequence ID: ACI88877.1 Length: 271

Range 1: 1 to 261
Score:422 bits(1085), Expect:4e-137,
Method:Compositional matrix adjust.,
Identities:260/261(99%), Positives:260/261(99%), Gaps:0/261(0%)

Query  11913  MEIEVLGPLDIRLDGTSIVPSAGKPRQIlallalraGRIVPVPVLMEEIWGDRIPRSAQT  12092
Sbjct  1      ............................................................  60

Query  12093  TLQTYILQLRRRISAARPDVRRPTAKDVLSTRFGGYLLSEPVLSSDVGTFQRLTaegsaa  12272
Sbjct  61     ............................................................  120


Query  12273  lergeaglaaDVLGRALSLWHGSALIDVPTGHVLDTEILGieeararalelrieadlrlG  12452
Sbjct  121    .................G..........................................  180
Query  12453  RHAELLGELRMLVAQHPMHESFHAQLMIALCRSGHTWRALDVYQQLRSALVGELGVEPSD  12632
Sbjct  181    ............................................................  240

Query  12633  RIQRLHQQVLGGGLDKPRSTY  12695
Sbjct  241    .....................  261

>Aln8 [Streptomyces sp. CM020]
Sequence ID: ACI88878.1 Length: 185

Range 1: 29 to 185
Score:325 bits(834), Expect:1e-104,
Method:Compositional matrix adjust.,
Identities:154/157(98%), Positives:155/157(98%), Gaps:0/157(0%)

Query  11090  APAGAQQDARGSAKAVAMTELDFLLGDYTCAYTDLTLEEPTTVTLNWDTKKTLEGKFYEM  11269
Sbjct  29     ................S...........................................  88

Query  11270  HLKSPAFEGRWVFGLNTVDNRYTSFYWDTWGNTGTASSVGWKRDMLRFQGPYITPGGHAD  11449
Sbjct  89     ......................................P....G................  148
```

```
Query  11450  SKDEFRVVNSDRFTDDAFIRFEGQPWKQISHVDCRRS  11560
Sbjct  149    .....................................  185
```

```
>AlnT1 transporter [Streptomyces sp. CM020]
Sequence ID: ACI88879.1 Length: 513

Range 1: 1 to 512
Score:781 bits(2017), Expect:0.0,
Method:Compositional matrix adjust.,
Identities:498/512(97%), Positives:501/512(97%), Gaps:1/512(0%)

Query  9258   MTVEEQADTAPPAQYSPKRWATLGVTLFAVFMDMVDNTVLNVALPAVQQDLDASSAQLEW  9437
Sbjct  1      .......................A....................................  60

Query  9438   SVAGYTLAFAAAMITGARLGDQLGRRRIYLIGLGAFVVTSALAGAAVNPEMLIASRILQG  9617
Sbjct  61     ............................................................  120

Query  9618   AAAALMVPQVLAMLQVDFPKSERPKAMSMYGMSlavggiggpllggvllEADLFGLGWRP  9797
Sbjct  121    G...........................................................  180

Query  9798   VFYVNVPVGlaalvaaailTRESRVETRESFDIRGTLIATVGLISLLFPLVQGRELDWPW  9977
Sbjct  181    ............................................................  240

Query  9978   WTFALMIACPVILWLFVRYEHRVIARGESPIIDPAllhhrsslggllVAILFFCGMAYQL  10157
Sbjct  241    ............................................................  300

Query  10158  VLTVHLQTGEGYSPLRTAVALVTFTVGVGIGSAVAPQLMPLGRRVVLLGCAVMAVGMGVI  10337
Sbjct  301    ..............I.............................................  360

Query  10338  TWTVDHYSGSLEWWHLAPGMIVSGIGLAMVAGTLLTIVLAQMPKSASGAASSLINTaiqi  10517
Sbjct  361    ......W.....................................................  420

Query  10518  gvatgvaivgtvYFTLLEDRHTPTDSavvglltvvglytlagllaFVLPPGRvdvsdvda  10697
Sbjct  421    ............................................L..............S  480

Query  10698  dtdtdadfd-hhhataaGTALPPEAGTKARVA  10790
Sbjct  481    .A.....S.H..ATA.V.........A.....  512

>AlnR8 MarR family regulator [Streptomyces sp. CM020]
Sequence ID: ACI88880.1 Length: 166

Range 1: 1 to 166
Score:299 bits(766), Expect:6e-96,
Method:Compositional matrix adjust.,
Identities:164/166(99%), Positives:166/166(100%), Gaps:0/166(0%)

Query  9166  MSSDGRQGAGLSVDNGVRTLLLLMPRVVARTKRTPVPQQLEEYNLAPRHLSLLAYLFFDG  8987
Sbjct  1     ............................................................  60
Query  8986  PLAVSELATRLELVPATVSLLVGELNRYGVVdrkedeadrrrKIVSIAEPYREAVRAWLE  8807
Sbjct  61    ........................................................S...  120

Query  8806  NGVNAWRVALEPLSPAERQTFIDTLRVYERELMGAQERQSVAATAD  8669
Sbjct  121   .......................................A......  166

Range 2: 4 to 149
Score:128 bits(322), Expect:3e-36,
Method:Compositional matrix adjust.,
Identities:77/148(52%), Positives:101/148(68%), Gaps:2/148(1%)

Query  30759  EAEAGSRLSAEEAVRAMLVTLPRLVSRAKRTPVPEQLRSLRLAPRHLSLLSCLIFDGPTS  30580
Sbjct  4      DGRQ.AG..VDNG..TL.LLM..V.A.T......Q..EEYN.........AY.F....LA  63

Query  30579  VKDLAARLEVAPTTVSLMVSDLQREGvverrsdpddrrrsivSLTEDPTTRAAVDAWLAS  30400
Sbjct  64     .SE..T...LV.A....L.GE.N.Y...D.KE.EA....K...IA.--PY.E..RS..EN  121

Query  30399  GARAWRKVFDDLSPQERATFVHAIQRYE  30316
Sbjct  122    .VN...VALEP...A..Q..IDTLRV..  149

>AlnR4 two component sensory kinase [Streptomyces sp. CM020]
Sequence ID: ACI88881.1 Length: 430
```

```
Range 1: 1 to 429
Score:698 bits(1802), Expect:0.0,
Method:Compositional matrix adjust.,
Identities:406/433(94%), Positives:413/433(95%), Gaps:4/433(0%)

Query  8563  VNDCGGRSMYAQGESYVVVYAENRLhdydhhddnhydfdsDGSESGIGVELGATGLRLALF  8384
Sbjct  1     M......................I......Y.YDS.----.G.P.................  56

Query  8383  ITGAVQLGYALMAILNLLKWHHESLSLVGAVLILIAFFGLQLFHCNPYAVHLRARIGPWT  8204
Sbjct  57    ..C.....................M...................................  116

Query  8203  LVPQAALAYAPFPMfgvlwggfggflsgavlvvLRGSALAWVLFVLNAAAVFGLALGSFA  8024
Sbjct  117   .....................................V.............C....A...  176

Query  8023  LFPSLYLTLATVVIGLMVYGLTRLSDIVVEQYRLRHRTALAAVSGERLRVARDLHDLLGY  7844
Sbjct  177   .A..V.......................................................  236

Query  7843  SLSAITLKSELTLRKVGVDDDRARKELVETLNIARQALADVRAVARGCRNLSVTAELRSV  7664
Sbjct  237   ............................................................  296

Query  7663  SGVLDAADVETTIEGEPGDLDPGTGSILAIVLREAVTNLLRHSAARHCRIEFGETDGQRW  7484
Sbjct  297   ..........................V...........................A.....  356

Query  7483  LAISNDGVQDGMSRTEagrrggglgnladrlATVGGELNVTTEWGWFHLRAVLPARPAVP  7304
Sbjct  357   ........E..V...................G........................A...  416

Query  7303  QPRPGRQPTPHHA  7265
Sbjct  417   P........H...  429

>AlnR5 two component response regulator [Streptomyces sp. CM020]
Sequence ID: ACI88882.1 Length: 201

Range 1: 1 to 201
Score:388 bits(996), Expect:3e-126,
Method:Compositional matrix adjust.,
Identities:198/201(99%), Positives:201/201(100%), Gaps:0/201(0%)

Query  6641  VIRLIIAEDVPMLRGALVALMELEQDLSVVAEVGNGNDILPTALEHRPDIAVIDIDLPGT  6820
Sbjct  1     M...........................................................  60

Query  6821  DGLTAAAKLRSCLPSCRVLIITSLGNPAALRRALAAQVDGYVLKDALPSELAQAIRKVAA  7000
Sbjct  61    ............................................................  120

Query  7001  GQRVIDPQLALLAWDGPAQQLTPREVDVLRLAAAGEDVRVIAKELHLSVGTVRNYLTTIV  7180
Sbjct  121   ...........M....................................I...........  180


Query  7181  HKLGARNRVDAVRIARDNGVI  7243
Sbjct  181   .....................  201

Range 2: 1 to 201
Score:108 bits(269), Expect:2e-28,
Method:Compositional matrix adjust.,
Identities:76/215(35%), Positives:111/215(51%), Gaps:14/215(6%)

Query  3814  MTTVLIVDDQALQRLGFSMLLEQHSDLTVVGEATHGAEAVRLTAELRPDVVLMDVRMPGM  3993
Sbjct  1     .IRLI.AE.VPML.GALVA.M.LEQ..S..A.VGN.NDILPTAL.H...IAVI.IDL..T  60

Query  3994  DGIEATRRIVESGGRSRVLVLTTFDLDEYAYAALRAGASGFLLKDALPDELTAGIRAVAS  4173
Sbjct  61    ..LT.AAKLRSCLPSC...II.SLGNPAALRR..A.QVD.YV......S..AQA..K..A  120

Query  4174  GDAVIAPGLTRKLIDAFSAHLPGTTPAQDRQLTALTTREREVLTAIATGWSNAEIATRFS  4353
Sbjct  121   .QR..D.Q.ALMAW.G---------...Q-----..P..VD..RLA.A.EDVRV..KELH  166

Query  4354  LAESTVKSHVSHILAKIGARDRVQAVIFAYDMGLV  4458
Sbjct  167   .SIG..RNYLTT.VH.L...N..D..RI.R.N.VI  201

>AlnI starter unit ketoacyl synthase [Streptomyces sp. CM020]
Sequence ID: ACI88883.1 Length: 332
```

```
Range 1: 1 to 331
Score:530 bits(1364), Expect:1e-173,
Method:Compositional matrix adjust.,
Identities:323/331(98%), Positives:324/331(97%), Gaps:0/331(0%)

Query  6023  MTRTVLTAGTRHSALLGIGGYRPRRVVGNAEICRLIESTEEWIETRSGIAERRFADDDet  5844
Sbjct  1     .............V......................D............V......H...  60

Query  5843  llmmaataaekalaqAGTTPAEVDLVLVASMSNLVQTPPLAVRVAHELgagaaagvdlsa  5664
Sbjct  61    ...................................................V........  120

Query  5663  acaGFCHALAMASDAVRAGSARRVLVVGAERMTDIVEPTDRTISFLFADGAGAVVVGDSD  5484
Sbjct  121   ............................................................  180

Query  5483  TPGIGPVVRRAYGAHSEALRMTAPWATADGAAPERPWMRMDGRRVFRWAMDEVAPALART  5304
Sbjct  181   ............................G.P.............................  240

Query  5303  VGEAGLTPAELGAFVPHQANLRMIELMTERLGLTGATAVSRDVVRSGNTSAASIPlalea  5124
Sbjct  241   .R..........................................................  300

Query  5123  llasqeaSTGDTALLVGFGAGLNFASQVVVL  5031
Sbjct  301   ...............................  331

>AlnH flavin reductase [Streptomyces sp. CM020]
Sequence ID: ACI88884.1 Length: 180

Range 1: 1 to 169
Score:293 bits(751), Expect:1e-93,
Method:Compositional matrix adjust.,
Identities:162/169(96%), Positives:165/169(97%), Gaps:0/169(0%)

Query  5028  VNRTDRRAVADVEPSQVRERFRAAMAQLAggvvvvttedtdGRPYGFTATSFCSVSMDPA  4849
Sbjct  1     M............R...G.....................A....................  60

Query  4848  LVLVCLAETSSSYEAFMDCRGFAVSLLGQEQRALATRFATTGADKFRAEDTVTTPRLLPA  4669
Sbjct  61    ............................................................  120

Query  4668  VEGALAVLDCDVHARHPAGDHMILVGAVRHVLPGRGEPLVYHDRAFQQL  4522
Sbjct  121   .............Y.......T............C..............  169

>AlnR6 two component response regulator [Streptomyces sp. CM020]
Sequence ID: ACI88885.1 Length: 218

Range 1: 1 to 218
Score:424 bits(1090), Expect:1e-138,
Method:Compositional matrix adjust.,
Identities:215/218(99%), Positives:215/218(98%), Gaps:0/218(0%)
Query  3814  MTTVLIVDDQALQRLGFSMLLEQHSDLTVVGEATHGAEAVRLTAELRPDVVLMDVRMPGM  3993
Sbjct  1     ........................P...................................  60

Query  3994  DGIEATRRIVESGGRSRVLVLTTFDLDEYAYAALRAGASGFLLKDALPDELTAGIRAVAS  4173
Sbjct  61    ............................................................  120

Query  4174  GDAVIAPGLTRKLIDAFSAHLPGTTPAQDRQLTALTTREREVLTAIATGWSNAEIATRFS  4353
Sbjct  121   ....................................................T...N...  180

Query  4354  LAESTVKSHVSHILAKIGARDRVQAVIFAYDMGLVRPA  4467
Sbjct  181   ......................................  218

Range 2: 4 to 217
Score:110 bits(274), Expect:5e-29,
Method:Compositional matrix adjust.,
Identities:77/214(36%), Positives:112/214(52%), Gaps:14/214(6%)

Query  6650  LIIAEDVPMLRGALVALMELEQDLSVVAEVGNGNDILPTALEHRPDIAVIDIDLPGTDGL  6829
Sbjct  4     VL.VD.QALQ.LGFSM.L.QHP..T..G.ATH.AEAVRLTA.L...VVLM.VRM..M..I  63

Query  6830  TAAAKLRSCLPSCRVLIITSLGNPAALRRALAAQVDGYVLKDALPSELAQAIRKVAAGQR  7009
Sbjct  64    E.TRRIVESGGRS...VL.TFDLDEYAYA..R.GAS.FL......D..TAG..A..S.DA  123
```

```
Query  7010  VIDPQL---------ALLAWDGPAQQ-----LTPREVDVLRLAAAGEDVRVIAKELHLSV  7147
Sbjct  124   ..A.G.TRKLIDAFS.H.PGTT...DRQLTA..T..RE..TAI.T.WSNTE..NRFS.AE  183

Query  7148  GTVRNYLTTIVHKLGARNRVDAVRIARDNGVI*P  7249
Sbjct  184   S..KSHVSH.LA.I...D..Q..IF.Y.M.LVR.  217
```

>AlnR7 two component sensory kinase [Streptomyces sp. CM020]
Sequence ID: ACI88886.1 Length: 381

Range 1: 1 to 381
Score:457 bits(1176), Expect:1e-147,
Method:Compositional matrix adjust.,
Identities:347/381(91%), Positives:360/381(94%), Gaps:0/381(0%)

```
Query  2672  VQNSAKGIGAPVPTLLALIVAFCVPLLWRQQRPVLVFaltsavsavalaadadtgaeaaR  2851
Sbjct  1     MRDA.N...P..Q............................V..S....L...S......  60

Query  2852  IVALLNVGRSVRPAQLAVCLGIAIAQTTVGVVVRGAEQPNEQFLQTPvlaivqsaliaav  3031
Sbjct  61    .............................A......G.......................  120

Query  3032  aaaglvgRVMNAYIRALHERAVRLEVerdqrarlaaaaerarvareMHDILGHTlavivg  3211
Sbjct  121   .........V..................................................  180

Query  3212  laggaaglTEAKPKRGAETLRIIADSgrgalaelrrllaVIGEERDTEDGRPLAPQPGLA  3391
Sbjct  181   ..........T.......................................S.........  240

Query  3392  DLDPLLERVRGAGPTVTLHTRGALTGLAPGLQLAVYRVVQESLTNTLKHAASDTRIHVAL  3571
Sbjct  241   ..............A..........D............................TVR.T.  300

Query  3572  TTDDTSVHATVEDAGPSRTPRSSAPREEGRGLVGMRERAALYGGSVTAGPNAQGGWTVEA  3751
Sbjct  301   ..E......R......PS..H.P..QG........................S......D.  360

Query  3752  HFQtattpppphtaptEKRPA  3814
Sbjct  361   ..R.T................  381
```

>AlnT2 transporter [Streptomyces sp. CM020]
Sequence ID: ACI88887.1 Length: 741

Range 1: 41 to 737
Score:1127 bits(2915), Expect:0.0,
Method:Compositional matrix adjust.,
Identities:654/697(94%), Positives:672/697(96%), Gaps:0/697(0%)

```
Query  122   TSDTFSIPGTQSQKALDLLEKEFPQASADGATARVVFEAPDGQKLTSAAHKAEVESLVDD  301
Sbjct  41    .....A..................................A.................G.  100


Query  302   LKSASQVASVADPYTGGTVSKDGSVAYAQVTYKVVPDEITDATRAGLEHVAEQGERAGLA  481
Sbjct  101   ........N.V.....................E.AQADV...A..D..............  160

Query  482   VSMGGSAVAEEAHQSTAELIGIVIAALVMVITFGSLVAAGLPLLTALFGVVAAICGITVA  661
Sbjct  161   .....T..N..S...A..................................L.........  220

Query  662   SSVIglssststlalmlglaVAIDYALFIVSRYRGELKEGHDPEEAAGRALGTAGSAVVF  841
Sbjct  221   T.F.D.......................................................  280

Query  842   AGLTVVIALAGLSVIGIKILTDiglgaafavviavvialtllpaMLGFAGTRISAGKLKT  1021
Sbjct  281   ....................S...........V.................G.........  340

Query  1022  RRMRAVERGEREPMGVRWSQFVLRNPVKvlgasvaglllavpalslqlgMAGDEMAAPG  1201
Sbjct  341   .....I.Q..........AR...........V.........I.........P..ST.T..  400

Query  1202  STQRIAYDTVTDGFGAGYNGPLTVVVDARGSDDPKAAAQDAVTLLEDLPDVASVSPASFN  1381
Sbjct  401   .............................................D..........V...  460

Query  1382  GTGDVALIRAVPGSSPTSEDTVALVSDIRDRGPALHDDTGAELMVTGTTALNIDISGKLN  1561
Sbjct  461   E.....................G.........A...........................  520

Query  1562  DALIPYLCVVVGLALILLMLVFRSILVPLKAAAGFLLSVLATLGVVVAVFQWGWFADVFG  1741
Sbjct  521   ......................................................L.....  580
```

```
Query  1742  VDQTGPIVSVLPIFMVGVVFGLAMDYQVFLVTRMREEYVHGAEPKEAVIAGFRHGARVVT  1921
Sbjct  581   ............................................................  640

Query  1922  AAAVIMISVFAGFLFSDTMLIKSIglglaaavfldafvvRMTIVPAVMALLGRRAWALPG  2101
Sbjct  641   .................A.............F..........................  700

Query  2102  RLDRILPNVDVEGEKLRHLLEEDTERTEGEGIPEPVH  2212
Sbjct  701   ............................D.......  737
```

Figure S2. BLASTX analysis of the alnumycin BGCs of CS 39 and *Streptomyces sp.* CM020. Query refers to the amino acid sequence of the BGC predicted for CS 39 (NCBI accession number OQ633075) and subject (sbjct) to the alnumycin BGC of *Streptomyces sp.* CM020 (NCBI accession number EU852062). Dots represent identities. Only differences are shown.

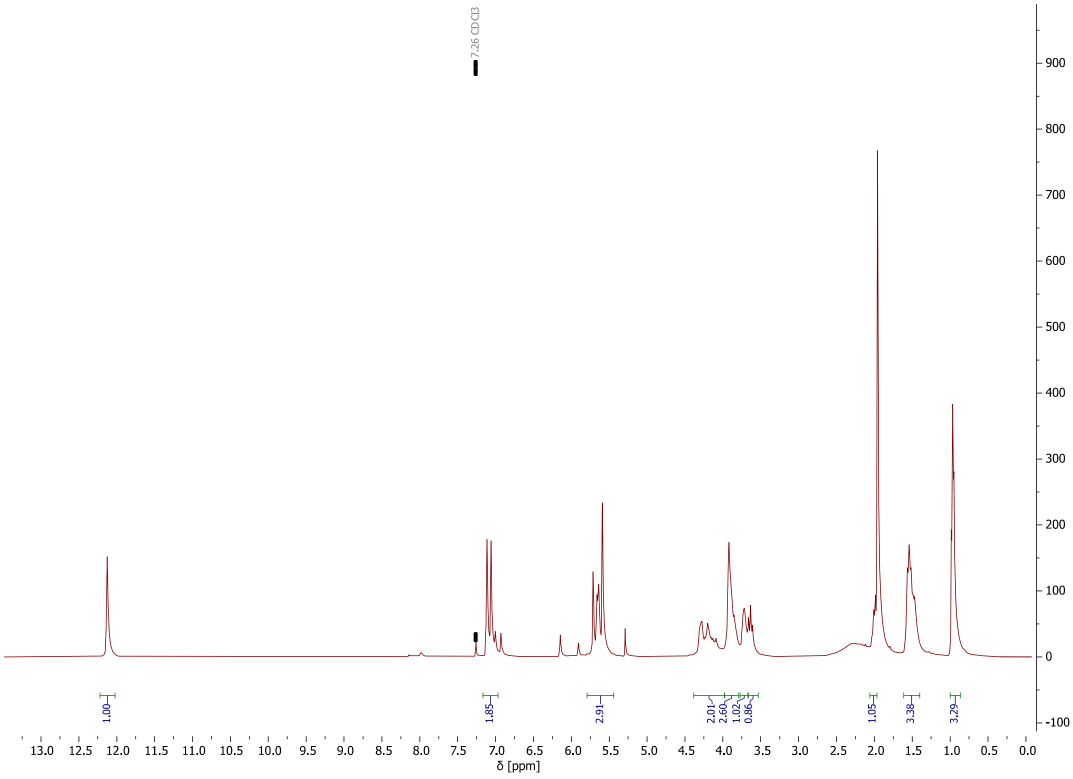

Figure S3. ^{1}H NMR spectrum of alnumycin in $CDCl_3$ (400 MHz).

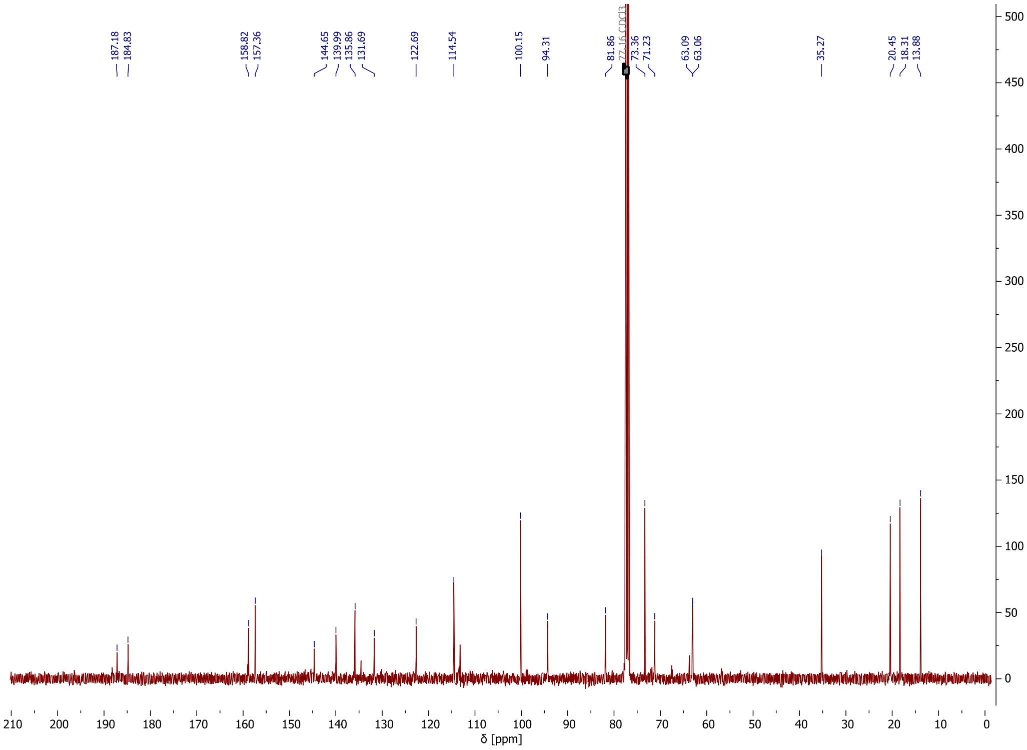

Figure S4. ^{13}C NMR spectrum of alnumycin in $CDCl_3$ (400 MHz).

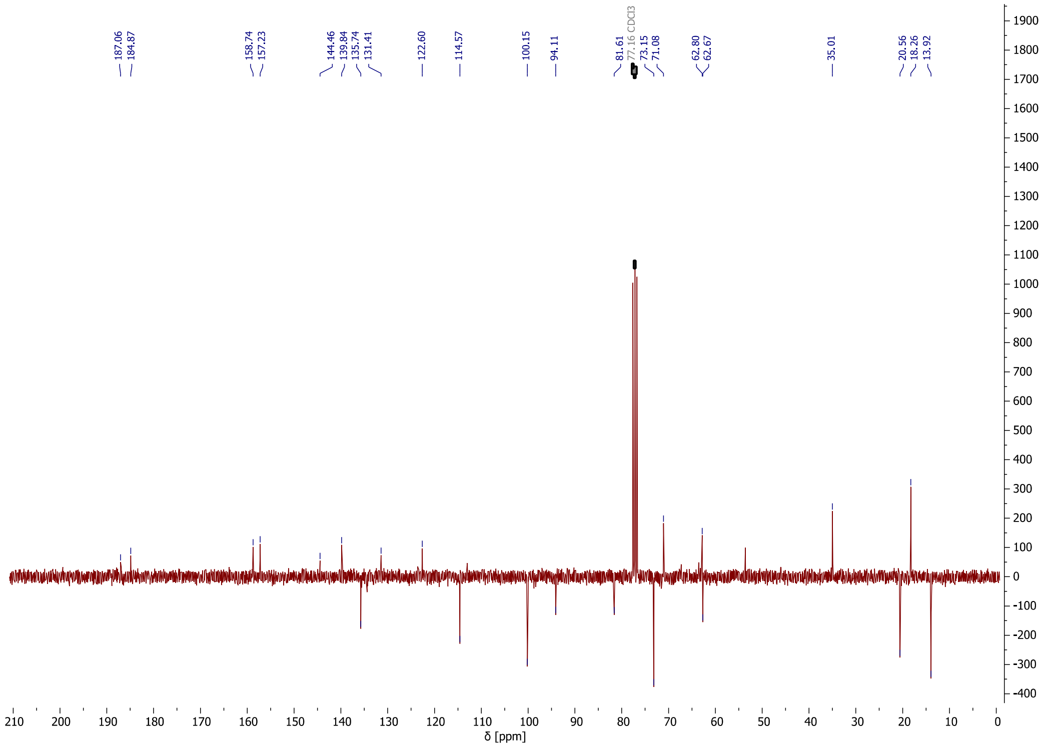

Figure S5. ^{13}C-DEPT NMR spectrum of alnumycin in $CDCl_3$ (400 MHz).

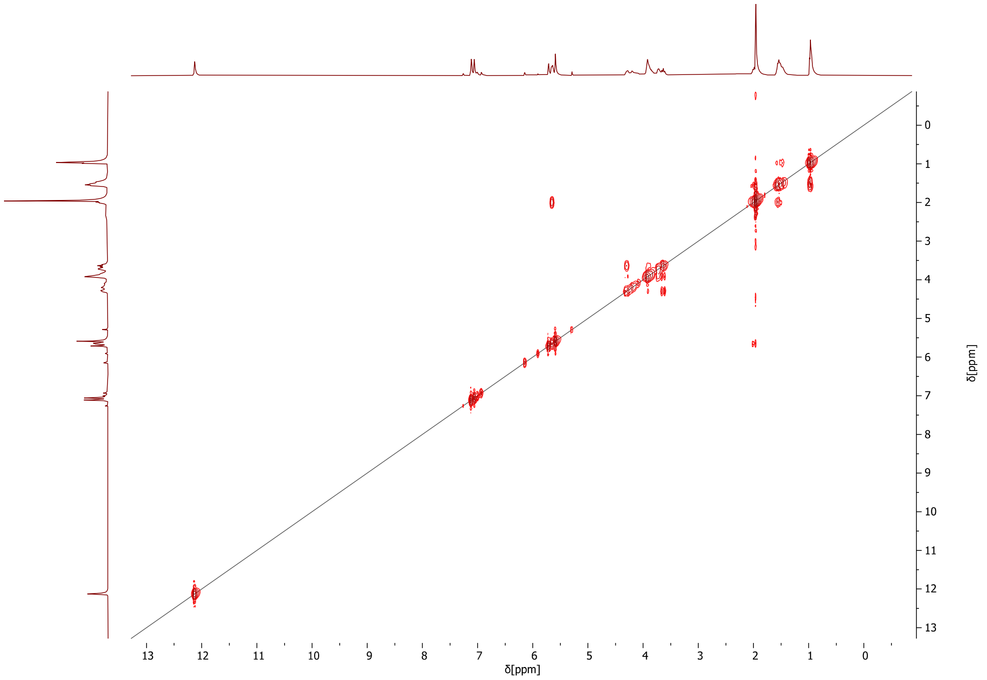

Figure S6. COSY of alnumycin in $CDCl_3$ (400 MHz).

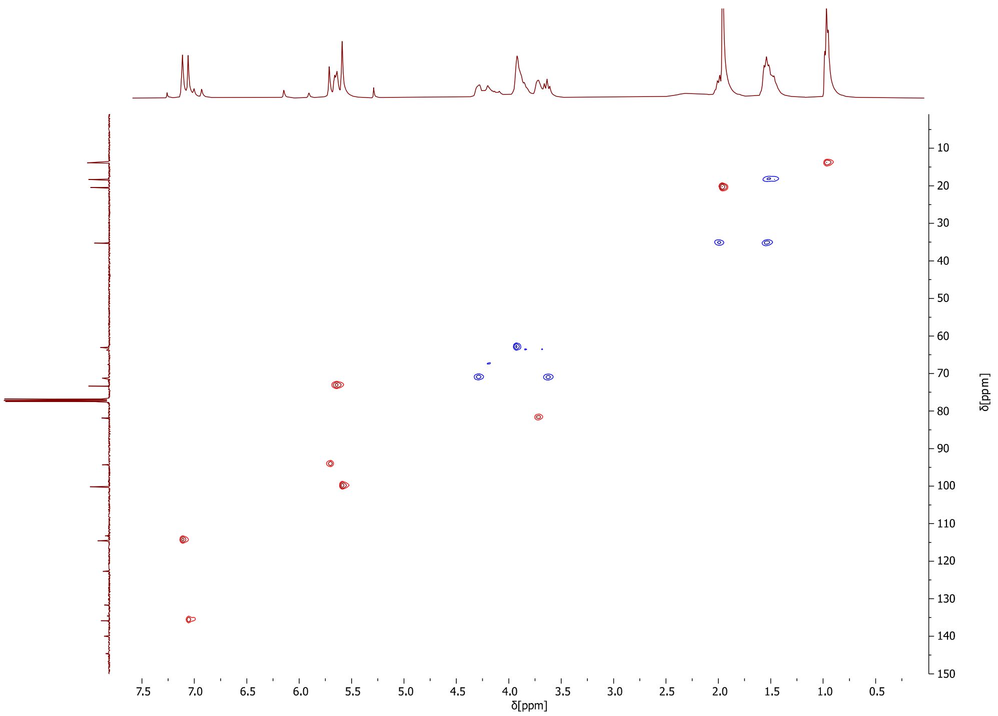

Figure S7. HSQC of alnumycin in $CDCl_3$ (400 MHz).

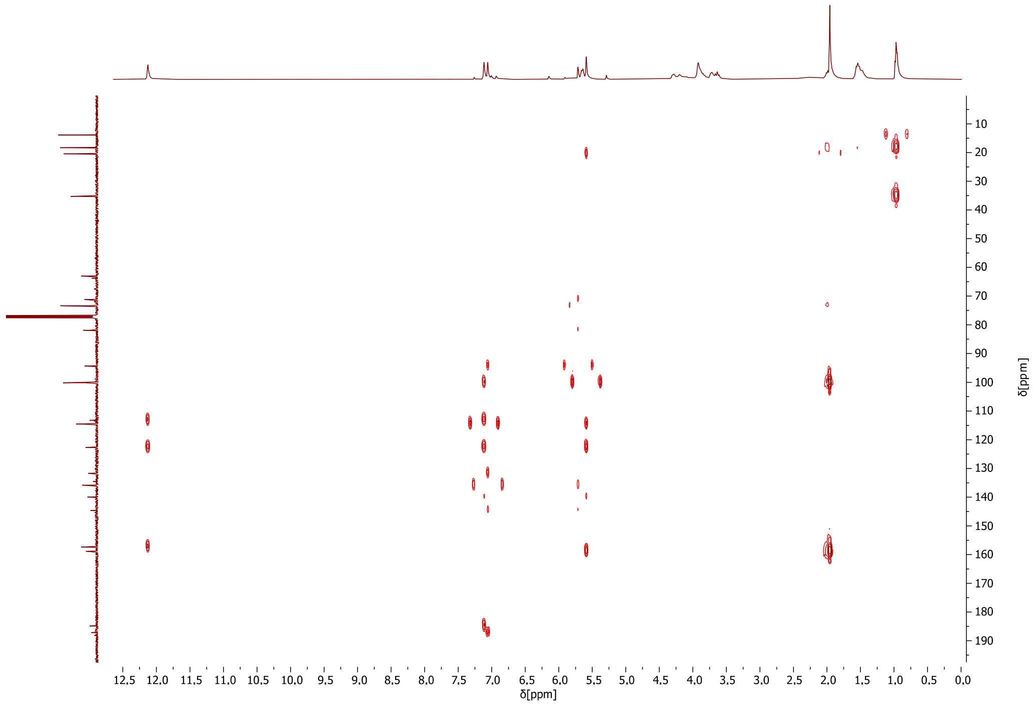

Figure S8. HMBC of alnumycin in $CDCl_3$ (400 MHz).

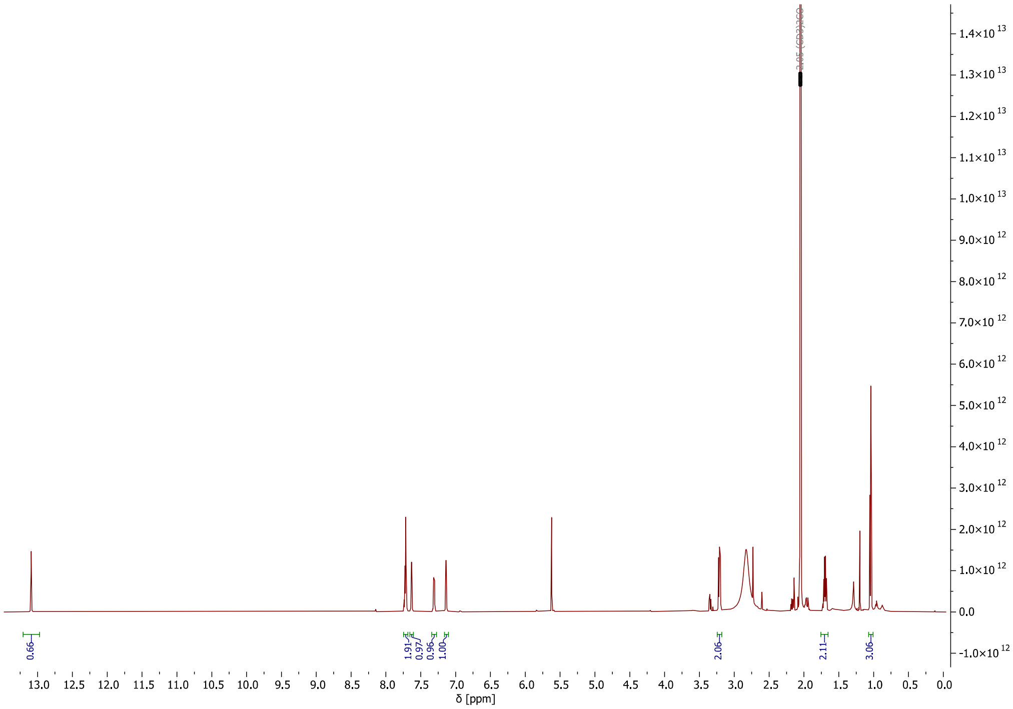

Figure S9. ^{1}H NMR spectrum of 1,6-dihydro 8-propylanthraquinone in acetone-d6 (600 MHz).

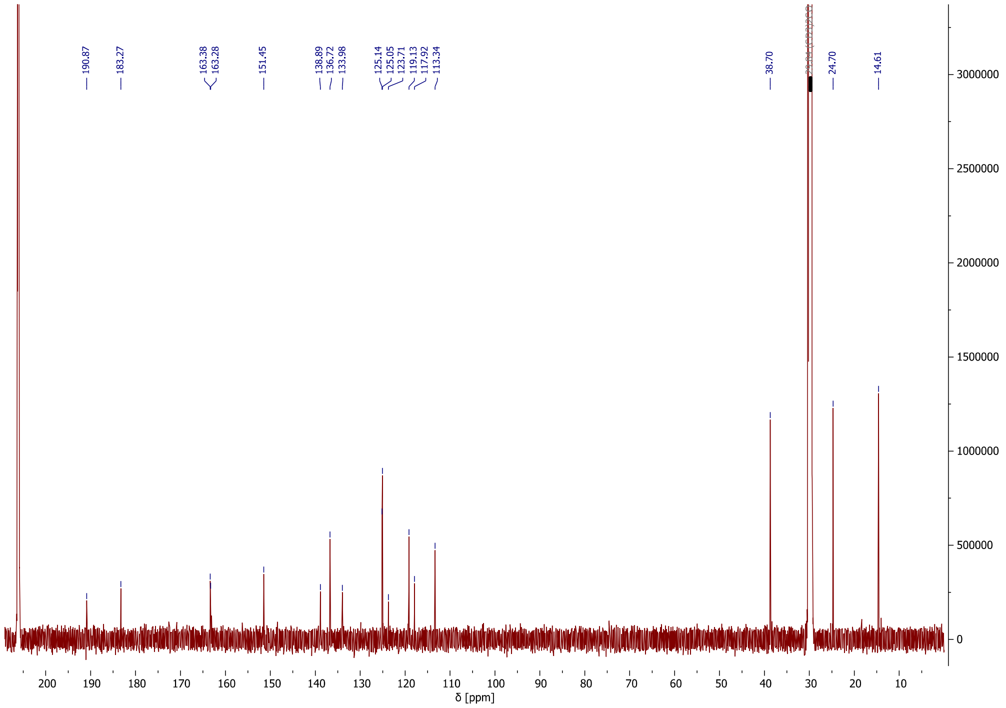

Figure S10. ^{13}C NMR spectrum of 1,6-dihydro 8-propylanthraquinone in acetone-d6 (600 MHz).

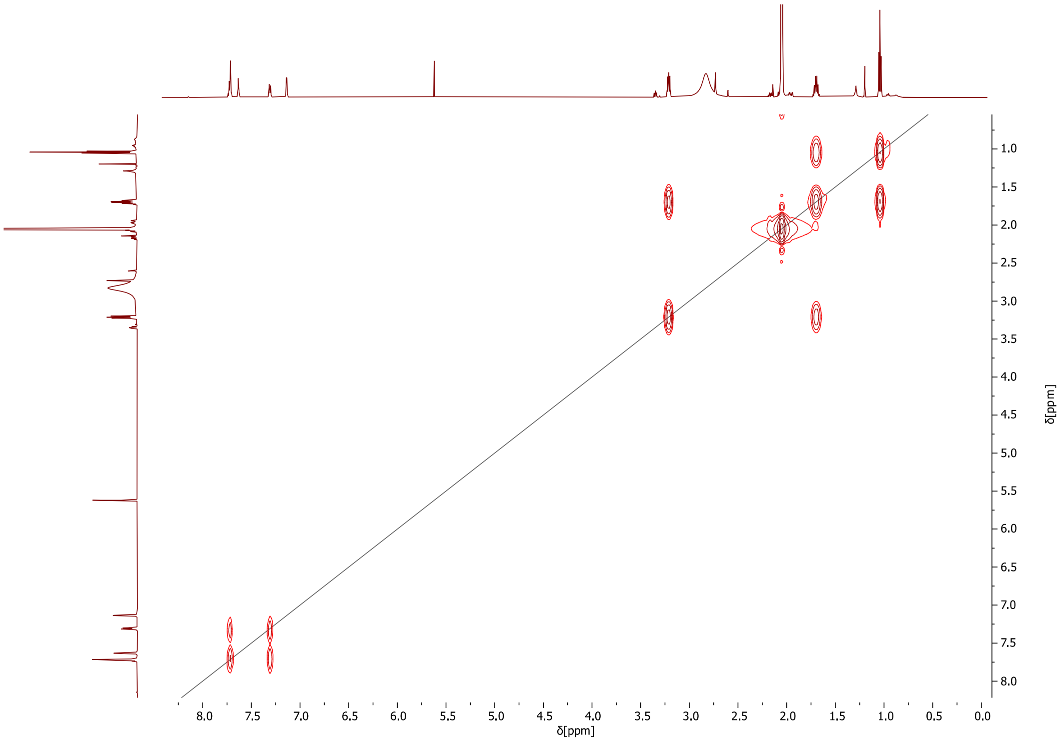

Figure S11. COSY of 1,6-dihydro 8-propylanthraquinone in acetone-d6 (600 MHz).

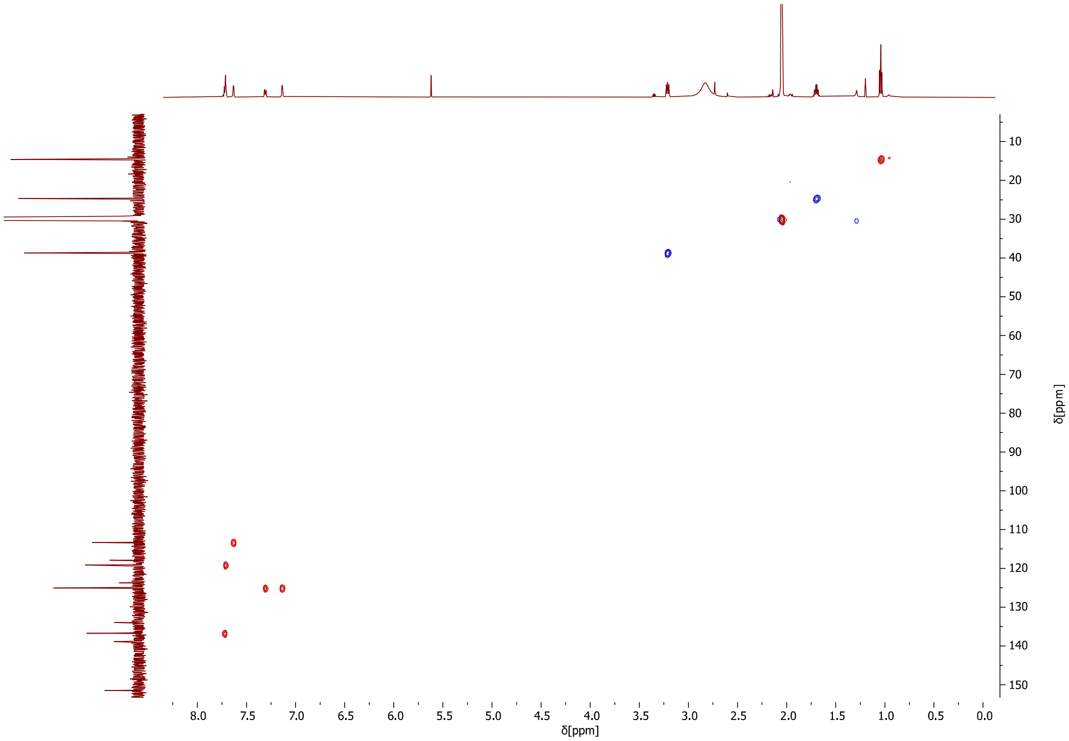

Figure S12. HSQC of 1,6-dihydro 8-propylanthraquinone in acetone-d6 (600 MHz).

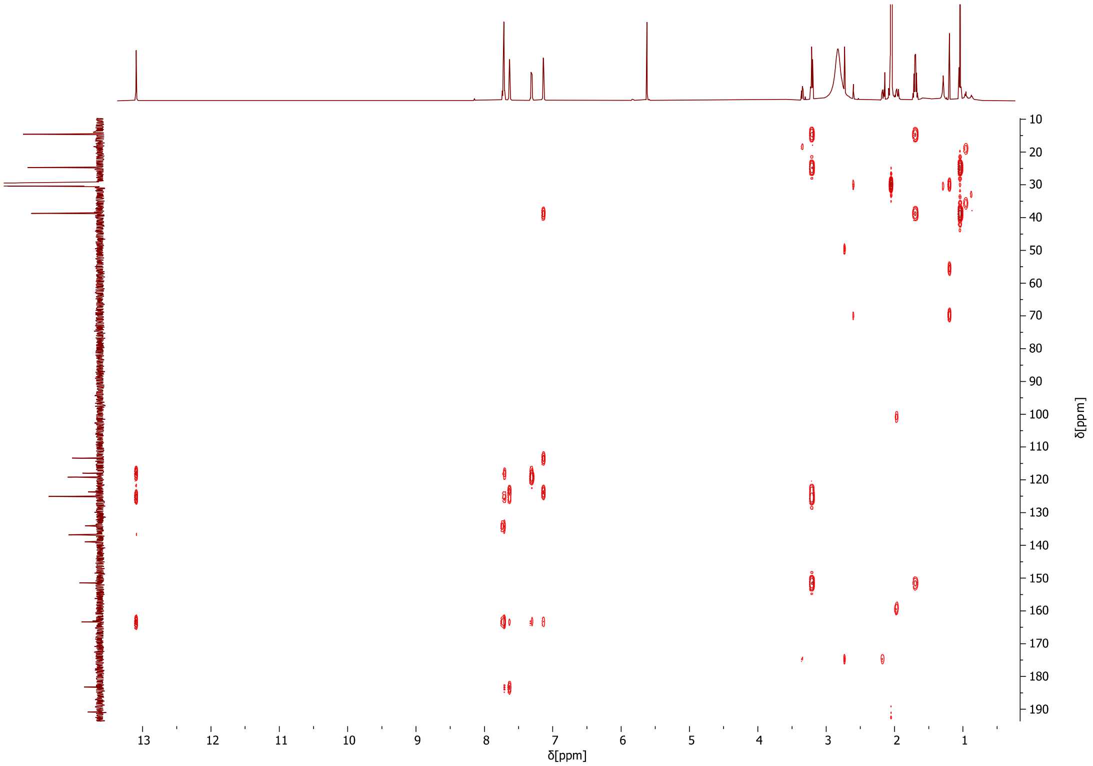

Figure S13. HMBC of 1,6-dihydro 8-propylanthraquinone in acetone-d6 (600 MHz).

(a)

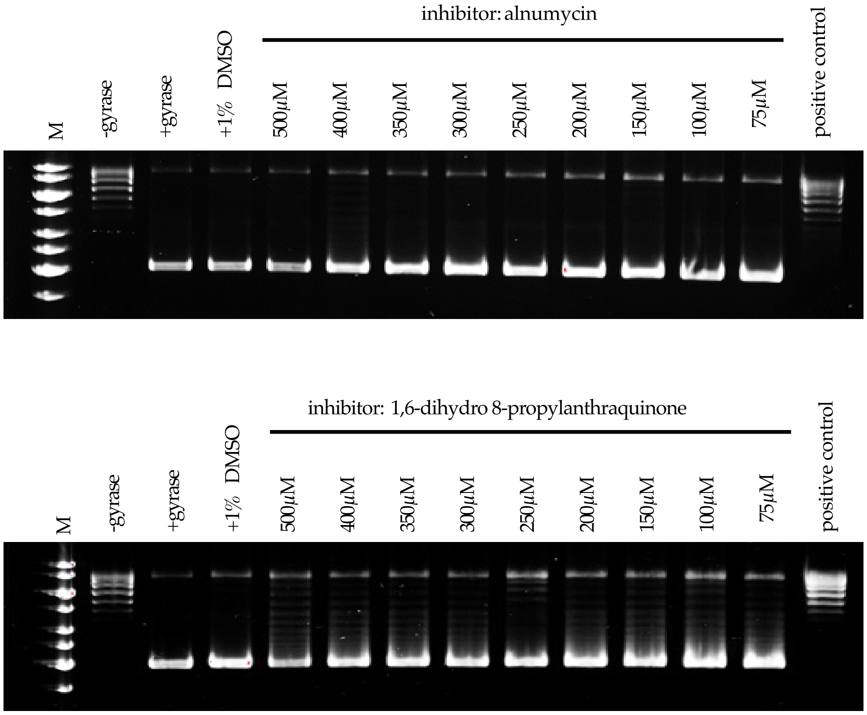

(b)

	IC_{50}[μM] (1)	IC_{50}[μM] (2)	IC_{50}[μM] (3)
**alnumycin**	>10	>10	>10
**1,6-dihydro 8-propylanthraquinone**	>10	>10	>10

Figure S14. Gyrase inhibition assays. The compounds were tested in **(a)** a gel-based and **(b)** a luciferase-based assay. **(a)** M = marker, - gyrase = reaction without gyrase, + gyrase = reaction with gyrase and without DMSO, + 1% DMSO = reaction with 1% DMSO and gyrase, 500 – 75 µM reactions with gyrase, 1% DMSO and indicated test compounds, positive control = reaction with gyrase and DMSO and 1 µM novobiocin. Agarose gels were run at 85 V. (b) The IC_{50} of tested compounds (N = 3).

Table S1. 16s rDNA similarity values d5 [%] for CS39 in comparison to other genomes of streptomycetal origin. 16s rDNA (NCBI accession number OQ632519) was extracted using RNAmmer 1.2 and evolutionary distances were calculated using the single-gene trees calculator provided by the DSMZ [1]. First described species names are given in bracket.

strains	NCBI accession number	similarity scores d5 [%]
Streptomyces umbrinus (*Streptomyces ederensis* NRRL B-8146)	EU594481	99.93
Streptomyces umbrinus EG1	OP363682	99.74
Streptomyces liliifuscus ZYC-3	MT767368	99.22
Streptomyces tauricus JCM 4837	MT760623	99.17
Streptomyces dioscori S32-77	LC551881	99.02
Streptomyces liliiviolaceus BH-SS-21	MW680653	98.81
Streptomyces aurantiacus (*Streptomyces glomeroaurantiacus* MD12-408-1-A)	KT446121	98.33
Streptomyces albicerus TRM68295	MK795696	97.99
Bacillus subtilis IAM 12118	NR_112116	85.6

Table S2. Alnumycin and 1,6-dihydro 8-propylanthraquinone levels in various production media. Normalized mean intensities are given in counts/mL culture volume. 10 mL culture volume were extract with equal amounts of organic solvents. The organic extracts were evaporated and redissolved in 500 ng/µL for mass spectrometry. The respective dilution factor was calculated and used to normalize intensities. Intensities of alnumycin were detected in positive, those of 1,6-dihydro 8-propylanthraquinone in negative ionization mode in biological triplicates (n = 3). n.d.: mass was not detected.

sample	alnumycin	1,6-dihydro 8-propylanthraquinone
**SFM supernatant**	n.d.	82,666
**SFM mycelium**	1,006	3,706
**PG3 supernatant**	313	751
**ISP2 Agar**	6,066	568
**GN supernatant**	3,465,777	241,762
**GN mycelium**	787,110	279,224
**GN + heat-killed *E. coli* cells mycelium**	240,822	59,692

Table S3. Growth inhibition assay for flash chromatography fractions. Mean final optical densities at 600 nm were determined after a 16-18 h incubation of test strains with flash chromatography fractions of crude extracts of CS 39 cultures. Extracts were dried and dissolved at 10 mg/mL in MeOH and tested at 100 µg/mL. The experiments were performed in biological triplicates. Standard deviations are shown below the mean values. Fractions showing inhibition of bacterial test strains are highlighted in green (cut-off of OD_{600} = 0.35).

fraction	*E. coli* Δ*tolC*	*B. subtilis* 168	*S. aureus* DSM 20231	*S. aureus* Mu50
**growth control**	0.819 +/- 0.018	0.578 +/- 0.005	0.707 +/- 0.012	0.751 +/- 0.023
**growth control MeOH**	0.810 +/- 0.022	0.543 +/- 0.073	0.714 +/- 0.068	0.730 +/- 0.027
**sterile control**	0.041 +/- 0.001	0.038 +/- 0.001	0.039 +/- 0.000	0.038 +/- 0.001
**1**	0.991 +/- 0.122	0.825 +/- 0.008	1.037 +/- 0.076	1.030 +/- 0.094
**2**	0.958 +/- 0.152	0.717 +/- 0.034	1.042 +/- 0.078	1.011 +/- 0.123
**3**	0.892 +/- 0.155	0.633 +/- 0.031	0.977 +/- 0.095	0.961 +/- 0.100
**4**	0.833 +/- 0.064	0.529 +/- 0.030	0.769 +/- 0.069	0.770 +/- 0.069
**5**	0.835 +/- 0.069	0.572 +/-0.003	0.862 +/- 0.066	0.885 +/- 0.062
**6**	0.774 +/- 0.121	0.454 +/- 0.014	0.587 +/- 0.071	0.703 +/- 0.093
**7**	0.677 +/- 0.083	0.059 +/- 0.006	0.057 +/- 0.001	0.059 +/- 0.006
**8**	0.708 +/- 0.073	0.530 +/- 0.017	0.107 +/- 0.068	0.142 +/- 0.104
**9**	0.697 +/- 0.159	0.528 +/- 0.018	0.307 +/- 0.245	0.217 +/- 0.183
**10**	0.058 +/- 0.004	0.064 +/- 0.001	0.062 +/- 0.001	0.061 +/- 0.003
**11**	0.474 +/- 0.048	0.482 +/- 0.123	0.071 +/- 0.006	0.074 +/- 0.003
**12**	0.911 +/- 0.032	0.657 +/- 0.025	0.222 +/- 0.161	0.109 +/- 0.042
**13**	0.981 +/- 0.068	0.712 +/- 0.015	0.211 +/- 0.117	0.177 +/- 0.028
**14**	0.890 +/- 0.063	0.585 +/- 0.020	0.519 +/- 0.045	0.422 +/- 0.048
**15**	0.827 +/- 0.021	0.401 +/- 0.039	0.373 +/- 0.009	0.336 +/- 0.017
**16**	0.776 +/- 0.041	0.417 +/- 0.035	0.685 +/- 0.201	0.695 +/- 0.120

fraction	*E. coli* **Δ*tolC***	***B. subtilis*** **168**	***S. aureus*** **DSM 20231**	***S. aureus*** **Mu50**
**growth control**	0.819 +/- 0.018	0.578 +/- 0.005	0.707 +/- 0.012	0.751 +/- 0.023
**growth control MeOH**	0.810 +/- 0.022	0.543 +/- 0.073	0.714 +/- 0.068	0.730 +/- 0.027
**sterile control**	0.041 +/- 0.001	0.038 +/- 0.001	0.039 +/- 0.000	0.038 +/- 0.001
**17**	0.775 +/- 0.032	0.512 +/- 0.016	0.884 +/- 0.048	0.818 +/- 0.070
**18**	0.777 +/- 0.041	0.481 +/- 0.039	0.833 +/- 0.083	0.815 +/- 0.056
**19**	0.788 +/- 0.003	0.565 +/- 0.024	0.429 +/- 0.081	0.415 +/- 0.061
**20**	0.809 +/- 0.025	0.589 +/- 0.150	0.453 +/- 0.020	0.548 +/- 0.177

Table S4. Alnumycin and 1,6-dihydro 8-propylanthraquinone in flash chromatography fractions. Extracts were dissolved at 10 mg/mL and diluted to 500 ng/µL for LC-MS. Detected intensities of both compounds are shown in counts. Intensities of alnumycin were detected in positive, those of 1,6-dihydro 8-propylanthraquinone in negative ionization mode. n.d.: the mass was not detected.

fraction	alnumycin	1,6-dihydro 8-propylanthraquinone
**1**	n.d.	n.d.
**2**	1.30E+03	
**3**	n.d.	n.d.
**4**	n.d.	n.d.
**5**	1.43E+04	n.d.
**6**	3.24E+05	n.d.
**7**	4.56E+05	n.d.
**8**	1.35E+05	n.d.
**9**	1.32E+04	8.91E+03
**10**	2.61E+03	1.59E+05
**11**	2.01E+03	4.03E+04
**12**	1.84E+03	8.73E+03
**13**	1.67E+03	n.d.
**14**	1.46E+03	n.d.
**15**	1.37E+03	n.d.
**16**	n.d.	n.d.
**17**	n.d.	n.d.
**18**	n.d.	n.d.
**19**	n.d.	n.d.
**20**	n.d.	n.d.

Table S5. Growth inhibition for preparative HPLC fractions of flash chromatography fraction 7. Mean final optical densities at 600 nm were determined after a 16-18 h incubation of test strains with preparative HPLC fractions of the flash chromatography fraction 7. Extracts were dried and dissolved at 10 mg/mL in MeOH and tested at 100 µg/mL. The experiments were performed in biological triplicates. Standard deviations are shown below the mean values. Fractions showing inhibition of bacterial test strains are highlighted in green (cut-off of OD_{600} = 0.35).

**fraction**	***E. coli* Δ*tolC***	***B. subtilis* 168**	***S. aureus* DSM 20231**	***S. aureus* Mu50**
**growth control**	0.884<br>+/- 0.001	0.662<br>+/- 0.052	0.917<br>+/- 0.085	0.893<br>+/- 0.062
**growth control MeOH**	0.917<br>+/- 0.023	0.775<br>+/- 0.034	0.921<br>+/- 0.045	0.923<br>+/- 0.024
**sterile control**	0.041<br>+/- 0.003	0.038<br>+/- 0.001	0.042<br>+/- 0.003	0.039<br>+/- 0.000
**7.1**	1.069<br>+/- 0.001	1.029<br>+/- 0.020	1.105<br>+/- 0.048	1.061<br>+/- 0.066
**7.2**	1.080<br>+/- 0.029	0.982<br>+/- 0.020	1.136<br>+/- 0.045	1.126<br>+/- 0.002
**7.3**	1.067<br>+/- 0.053	0.937<br>+/- 0.033	1.115<br>+/- 0.074	1.137<br>+/-0.008
**7.4**	1.000<br>+/- 0.055	0.835<br>+/- 0.102	1.044<br>+/- 0.090	1.070<br>+/- 0.015
**7.5**	0.954<br>+/- 0.040	0.775<br>+/- 0.064	1.034<br>+/- 0.119	1.045<br>+/- 0.002
**7.6**	1.037<br>+/- 0.001	0.804<br>+/- 0.081	1.078<br>+/- 0.053	1.097<br>+/- 0.010
**7.7**	1.052<br>+/- 0.029	0.879<br>+/- 0.069	1.082<br>+/- 0.014	1.068<br>+/- 0.015
**7.8**	0.928<br>+/- 0.057	0.693<br>+/- 0.060	0.997<br>+/- 0.075	0.980<br>+/- 0.090
**7.9**	0.615<br>+/- 0.021	0.300<br>+/- 0.073	0.319<br>+/- 0.041	0.317<br>+/- 0.083
**7.10**	0.717<br>+/- 0.088	0.552<br>+/- 0.046	0.718<br>+/- 0.061	0.828<br>+/- 0.168
**7.11**	0.879<br>+/- 0.003	0.674<br>+/- 0.095	0.913<br>+/- 0.083	0.965<br>+/- 0.000
**7.12**	0.911<br>+/- 0.017	0.704<br>+/- 0.082	0.983<br>+/- 0.076	1.032<br>+/- 0.015
**7.13**	0.828<br>+/- 0.000	0.728<br>+/- 0.013	1.064<br>+/- 0.039	1.024<br>+/- 0.001
**7.14**	0.999<br>+/- 0.018	0.836<br>+/- 0.054	1.063<br>+/- 0.032	1.051<br>+/- 0.056
**7.15**	0.980<br>+/- 0.027	0.784<br>+/- 0.052	1.003<br>+/- 0.064	1.001<br>+/- 0.064
**7.16**	0.922<br>+/- 0.023	0.766<br>+/- 0.086	0.944<br>+/- 0.017	0.925<br>+/- 0.017

fraction	*E. coli* **Δ*tolC***	***B. subtilis*** **168**	***S. aureus*** **DSM 20231**	***S. aureus*** **Mu50**
**growth control**	0.884 +/- 0.001	0.662 +/- 0.052	0.917 +/- 0.085	0.893 +/- 0.062
**growth control MeOH**	0.917 +/- 0.023	0.775 +/- 0.034	0.921 +/- 0.045	0.923 +/- 0.024
**sterile control**	0.041 +/- 0.003	0.038 +/- 0.001	0.042 +/- 0.003	0.039 +/- 0.000
**7.17**	0.908 +/- 0.004	0.721 +/- 0.049	0.925 +/- 0.012	0.902 +/- 0.012
**7.18**	0.906 +/- 0.018	0.725 +/- 0.057	0.926 +/- 0.133	0.876 +/- 0.011

Table S6. Growth inhibition for preparative HPLC fractions of flash chromatography fraction 10. Mean final optical densities at 600 nm were determined after a 16-18 h incubation of test strains with preparative HPLC fractions of the flash chromatography fraction 10. Extracts were dried and dissolved at 10 mg/mL in MeOH and tested at 100 µg/mL. The experiments were performed in biological triplicates. The calculated standard deviations are shown below the mean values. Fractions showing inhibition of bacterial test strains are highlighted in green (cut-off of OD_{600} = 0.35).

fraction	*E. coli* Δ*tolC*	*B. subtilis* 168	*S. aureus* DSM 20231	*S. aureus* Mu50
**growth control**	0.884<br>+/- 0.001	0.662<br>+/- 0.052	0.917<br>+/- 0.085	0.893<br>+/- 0.062
**growth control MeOH**	0.917<br>+/- 0.023	0.775<br>+/- 0.034	0.921<br>+/- 0.045	0.923<br>+/- 0.024
**sterile control**	0.042<br>+/- 0.004	0.043<br>+/- 0.006	0.047<br>+/- 0.000	0.047<br>+/- 0.001
**10.1**	0.651<br>+/- 0.040	0.587<br>+/- 0.151	0.897<br>+/- 0.213	0.773<br>+/- 0.242
**10.2**	0.818<br>+/- 0.197	0.643<br>+/- 0.102	1.080<br>+/- 0.005	0.861<br>+/- 0.094
**10.3**	0.927<br>+/- 0.026	0.716<br>+/- 0.040	1.052<br>+/- 0.059	0.993<br>+/- 0.014
**10.4**	0.942<br>+/- 0.031	0.796<br>+/- 0.155	0.944<br>+/- 0.050	0.931<br>+/- 0.038
**10.5**	0.917<br>+/- 0.024	0.768<br>+/- 0.153	0.881<br>+/- 0.075	0.886<br>+/- 0.026
**10.6**	1.000<br>+/- 0.144	0.691<br>+/- 0.153	0.909<br>+/- 0.032	0.843<br>+/- 0.038
**10.7**	0.871<br>+/- 0.003	0.675<br>+/- 0.105	0.846<br>+/- 0.042	0.818<br>+/- 0.008
**10.8**	0.840<br>+/- 0.012	0.656<br>+/- 0.073	0.827<br>+/- 0.064	0.774<br>+/- 0.040
**10.9**	0.349<br>+/- 0.042	0.438<br>+/- 0.016	0.348<br>+/- 0.193	0.112<br>+/- 0.052
**10.10**	0.359<br>+/- 0.035	0.434<br>+/- 0.027	0.167<br>+/- 0.128	0.073<br>+/- 0.005
**10.11**	0.794<br>+/- 0.046	0.548<br>+/- 0.054	0.780<br>+/- 0.143	0.754<br>+/- 0.027
**10.12**	0.864<br>+/- 0.014	0.598<br>+/- 0.112	0.912<br>+/- 0.031	0.925<br>+/- 0.033
**10.13**	0.694<br>+/- 0.027	0.582<br>+/- 0.069	0.822<br>+/- 0.043	0.682<br>+/- 0.066
**10.14**	0.980<br>+/- 0.052	0.618<br>+/- 0.131	1.109<br>+/- 0.055	1.056<br>+/- 0.061
**10.15**	0.932<br>+/- 0.021	0.773<br>+/- 0.065	0.955<br>+/- 0.079	0.976<br>+/- 0.036

**fraction**	***E. coli* Δ*tolC***	***B. subtilis* 168**	***S. aureus* DSM 20231**	***S. aureus* Mu50**
**10.16**	0.882 +/- 0.019	0.710 +/- 0.054	0.884 +/- 0.085	0.844 +/- 0.044

Table S7. ^{1}H and ^{13}C shifts of alnumycin. The sample was measured in $CDCl_3$ on a 400 MHz NMR spectrometer (Bruker).

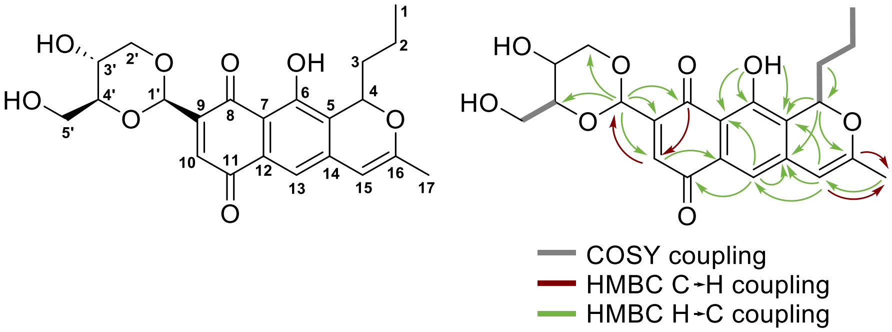

position	δH [ppm] (J in Hz)	δC [ppm]
**1**	0.97 (t, J = 6.8 Hz)	13.9
**2**	1.63 – 1.39 (m)	18.3
**3**	1.63 – 1.39 (m) and 2.05 – 1.87 (m)	35.3
**4**	5.76 – 5.46 (m)	73.4
**5**	---	122.7
**6**	---	157.4
**6-OH**	12.12 (s)	---
**7**	---	113.2
**8**	---	187.2
**9**	---	144.7
**10**	7.06 (s)	135.9
**11**	---	184.8
**12**	---	131.7
**13**	7.11 (s)	114.5
**14**	---	140.0
**15**	5.76 – 5.46 (m)	100.2
**16**	---	158.8
**17**	2.05 – 1.87 (m)	20.5
**1´**	5.76 – 5.46 (m)	94.3
**2´**	3.76 – 3.60 (m) and 4.38 – 4.06 (m)	71.2
**3´**	3.98 – 3.80 (m)	63.1
**3´-OH**	2.25 (bs)	---
**4´**	3.76 – 3.60 (m)	81.9
**5´**	3.98 – 3.80 (m)	63.1
**5´-OH**	2.25 (bs)	---

Table S8. ^{1}H and ^{13}C shifts of 1,6-dihydro 8-propylanthraquinone. The sample was measured in acetone-d6 on a 600 MHz NMR spectrometer (Bruker).

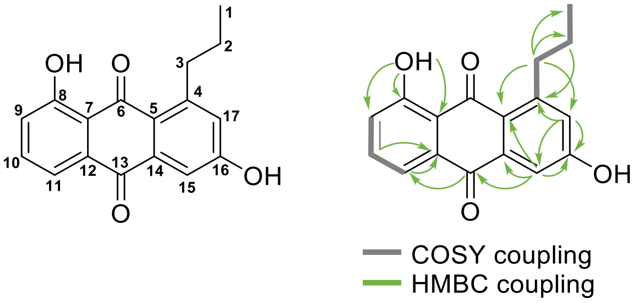

position	δ_H [ppm] (J in Hz)	δ_C [ppm]
**1**	1.04 (t, 7.3)	14.6
**2**	1.70 (h, 7.4)	24.7
**3**	3.23 – 3.20 (m)	38.7
**4**	---	151.5
**5**	---	123.7
**6**	---	190.9
**7**	---	117.9
**8**	---	163.4 o. 163.3
**8-OH**	13.09 (s)	---
**9**	7.31 (dd, 7.2, 2.3)	125.1
**10**	7.74 – 7.69 (m)	136.7
**11**	7.74 – 7.69 (m)	119.1
**12**	---	134.0
**13**	---	183.3
**14**	---	138.9
**15**	7.63 (d, 2.7)	113.3
**16**	---	163.4 o. 164.3
**17**	7.14 (d, 2.7)	125.1

Table S9. MICs of alnumycin. OD_{600} values are displayed in the table as well as the respective standard deviations of biological triplicates. MICs are highlighted in green. * Solution appeared clear (no bacterial growth) but coloration matched the 'extract only' sample.

concentration [µg/ml]	extract only	*E. coli* Δ*tolC*	*E. coli* DSM 30083	*A. baumannii* DSM 30007	*B. subtilis* 168	*S. aureus* DSM 20231	*S. aureus* Mu50
**growth control**	-	0.500 +/- 0.001	0.529 +/- 0.123	0.291 +/- 0.041	0.263 +/- 0.003	0.322 +/- 0.012	0.332 +/- 0.018
**growth control MeOH**	-	0.458 +/- 0.032	0.513 +/- 0.116	0.260 +/- 0.044	0.259 +/- 0.006	0.317 +/- 0.017	0.339 +/- 0.014
**sterile control**	-	0.042 +/- 0.000	0.042 +/- 0.000	0.040 +/- 0.000	0.041 +/- 0.000	0.041 +/- 0.000	0.041 +/- 0.001
**100**	0.173 +/- 0.007	* 0.164 +/- 0.012	0.535 +/- 0.022	0.460 +/- 0.007	* 0.171 +/- 0.006	* 0.170 +/- 0.004	* 0.179 +/- 0.005
**80**	0.183 +/- 0.003	0.564 +/- 0.046	0.540 +/- 0.007	0.435 +/- 0.021	* 0.178 +/- 0.005	* 0.251 +/- 0.063	* 0.176 +/- 0.012
**40**	0.103 +/- 0.005	0.759 +/- 0.012	0.527 +/- 0.011	0.437 +/- 0.034	* 0.102 +/- 0.008	* 0.201 +/- 0.019	* 0.111 +/- 0.008
**20**	-	0.797 +/- 0.010	0.536 +/- 0.008	0.447 0.036	0.080 +/- 0.005	0.095 +/- 0.004	0.088 +/- 0.003
**10**	-	0.375 +/- 0.012	0.516 +/- 0.031	0.417 +/- 0.061	0.053 +/- 0.001	0.054 +/-0.001	0.067 +/- 0.016
**8**	-	0.868 +/- 0.016	0.535 +/- 0.041	0.398 +/- 0.012	0.056 +/- 0.003	0.090 +/- 0.007	0.056 +/- 0.003
**4**	-	0.883 +/- 0.022	0.543 +/- 0.038	0.386 +/- 0.015	0.458 +/- 0.074	0.216 +/- 0.043	0.427 +/- 0.040
**2**	-	0.883 +/- 0.013	0.584 +/- 0.028	0.355 +/- 0.025	0.502 +/- 0.047	0.237 +/- 0.053	0.485 +/- 0.023
**1**	-	0.482 +/- 0.039	0.574 +/- 0.019	0.347 +/- 0.064	0.256 +/- .0011	0.371 +/- 0.041	0.395 +/- 0.022
**0.1**	-	0.486 +/- 0.073	0.534 +/- 0.093	0.304 +/- 0.058	0.266 +/- 0.010	0.339 +/- 0.012	0.378 +/- 0.012

Table S10. MICs of 1,6-dihydro 8-propylanthraquinone. Detected OD_{600} values are displayed in the table as well as the respective standard deviation of biological triplicates. Highlighted in green are the OD_{600} values recorded as MIC. *Solution appeared clear (no bacterial growth) but coloration matched the 'extract only' sample.

concentration [µg/ml]	extract only	*E. coli* Δ*tolC*	*E. coli* DSM 30083	*A. baumannii* DSM 30007	*B. subtilis* 168	*S. aureus* DSM 20231	*S. aureus* Mu50
**growth control**	-	0.491 +/- 0.003	0.602 +/- 0.016	0.291 +/- 0.025	0.263 +/- 0.005	0.332 +/- 0.011	0.361 +/- 0.035
**growth control MeOH**	-	0.476 +/- 0.016	0.585 +/- 0.015	0.247 +/- 0.025	0.264 +/- 0.006	0.301 +/- 0.010	0.353 +/- 0.041
**sterile control**	-	0.042 +/- 0.000	0.042 +/- 0.000	0.042 +/- 0.000	0.041 +/- 0.001	0.041 +/- 0.001	0.041 +/- 0.000
**100**	0.292 +/- 0.058	* 0.289 +/- 0.040	0.516 +/- 0.016	0.557 +/- 0.009	* 0.307 +/- 0.003	* 0.302 +/- 0.021	* 0.268 +/- 0.014
**80**	0.243 +/- 0.004	* 0.221 +/- 0.010	0.523 +/- 0.014	0.523 +/- 0.005	* 0.232 +/- 0.006	* 0.251 +/- 0.063	* 0.211 +/- 0.037
**40**	0.203 +/- 0.003	* 0.234 +/- 0.033	0.537 +/- 0.012	0.427 +/- 0.017	* 0.180 +/- 0.019	* 0.201 +/- 0.019	* 0.218 +/- 0.018
**20**	-	0.170 +/- 0.022	0.565 +/- 0.025	0.381 +/- 0.022	0.142 +/- 0.018	0.095 +/- 0.004	0.103 +/- 0.011
**10**	-	0.089 +/- 0.013	0.487 +/- 0.010	0.359 +/- 0.023	0.101 +/- 0.017	0.066 +/-0.014	0.067 +/- 0.016
**8**	-	0.149 +/- 0.011	0.553 +/- 0.018	0.371 +/- 0.035	0.178 +/- 0.010	0.090 +/- 0.007	0.072 +/- 0.015
**4**	-	0.173 +/- 0.002	0.497 +/- 0.021	0.352 +/- 0.027	0.322 +/- 0.105	0.216 +/- 0.043	0.162 +/- 0.007
**2**	-	0.286 +/- 0.012	0.547 +/- 0.025	0.321 +/- 0.035	0.343 +/- 0.031	0.237 +/- 0.053	0.212 +/- 0.030
**1**	-	0.456 +/- 0.059	0.568 +/- 0.036	0.309 +/- 0.008	0.250 +/- .0012	0.201 +/- 0.021	0.229 +/- 0.009
**0.1**	-	0.508 +/- 0.054	0.591 +/- 0.036	0.287 +/- 0.000	0.266 +/- 0.001	0.337 +/- 0.038	0.372 +/- 0.009

References

1. Meier-Kolthoff,J.P.; Carbasse,J.S.; Peinado-Olarte,R.L.; Göker,M. TYGS and LPSN: a database tandem for fast and reliable genome-based classification and nomenclature of prokaryotes *Nucleic. Acids. Res.* **2022**, *50*, D801–D807. https://doi.org/10.1093/nar/gkab902.
